# Synthesis of acenaphthylene-fused heteroarenes and polyoxygenated benzo[*j*]fluoranthenes via a Pd-catalyzed Suzuki–Miyaura/C–H arylation cascade

**DOI:** 10.3762/bjoc.20.273

**Published:** 2024-12-23

**Authors:** Merve Yence, Dilgam Ahmadli, Damla Surmeli, Umut Mert Karacaoğlu, Sujit Pal, Yunus Emre Türkmen

**Affiliations:** 1 Department of Chemistry, Faculty of Science, Bilkent University, Ankara 06800, Türkiye; 2 UNAM – National Nanotechnology Research Center, Institute of Materials Science and Nanotechnology, Bilkent University, Ankara 06800, Türkiye

**Keywords:** acenaphthylene-fused heteroarenes, benzo[*j*]fluoranthenes, C–H arylation, fluoranthenes, heterocycles

## Abstract

Acenaphthylene-fused heteroarenes with a variety of five- and six-membered heterocycles such as thiophene, furan, benzofuran, pyrazole, pyridine and pyrimidine were synthesized via an efficient Pd-catalyzed reaction cascade in good to high yields (45–90%). This cascade involves an initial Suzuki–Miyaura cross-coupling reaction between 1,8-dihalonaphthalenes and heteroarylboronic acids or esters, followed by an intramolecular C–H arylation under the same conditions to yield the final heterocyclic fluoranthene analogues. The method was further employed to access polyoxygenated benzo[*j*]fluoranthenes, which are all structurally relevant to benzo[*j*]fluoranthene-based fungal natural products. The effectiveness of our strategy was demonstrated via a concise, four-step synthesis of the tetramethoxybenzo[*j*]fluoranthene derivative **18**, which represents a formal total synthesis of the fungal natural product bulgarein.

## Introduction

**Figure 1:**
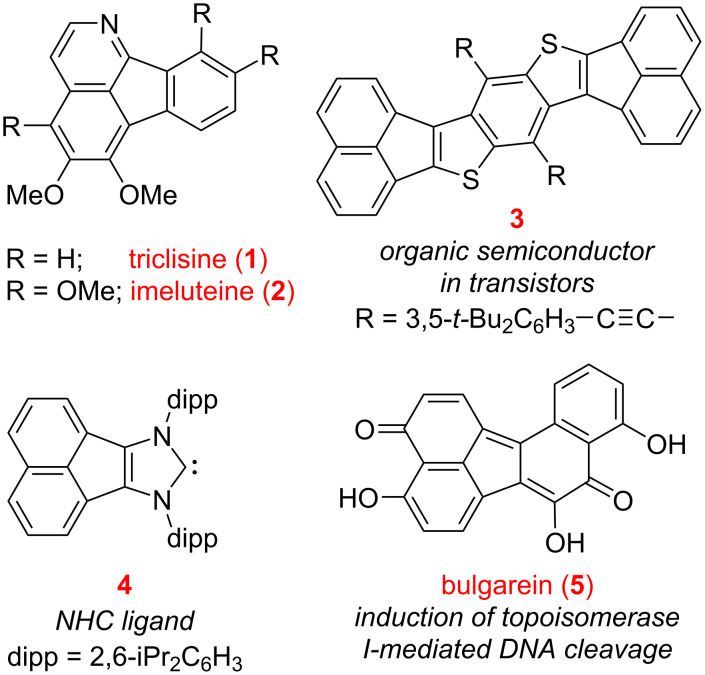
Examples of important azafluoranthene and benzo[*j*]fluoranthene natural products, and acenaphthylene-fused heterocycles.

An important subclass of polycyclic aromatic hydrocarbons (PAHs) [1] is comprised of fluoranthenes, which have been the focus of extensive research within the past two decades due to their interesting structural features, attractive photophysical properties, and diverse applications [2]. Indeed, fluoranthenes are particularly useful in supramolecular chemistry, organic electronics, and materials science [3] as fluorescent chemosensors and probes [4–6], as well as materials for applications in organic light-emitting diodes (OLEDs) [7–9], organic field-effect transistors (OFETs) [10], and perovskite solar cells [11]. In this respect, replacing either one or more of the carbons or rings of a fluoranthene with heteroatoms or other heterocycles to obtain heterocyclic fluoranthene analogues offers numerous opportunities for structural and functional diversifications. For instance, azafluoranthenes such as triclisine (**1**) or imeluteine (**2**) constitute a common structural motif encountered in natural products isolated from certain plant species (Figure 1) [12]. The acenaphthylene-fused thiophene-based heteroarene **3** is another heterocyclic fluoranthene analogue, which was used as an organic semiconductor in transistors [13]. The synthesis and coordination complexes of the acenaphthylene-fused *N*-heterocyclic (NHC) ligand **4** were reported by Cowley and co-workers in 2010 [14]. In addition to heterocyclic fluoranthene analogues, highly oxygenated benzo[*j*]fluoranthenes are commonly encountered fungal natural products with important biological activities [15]. Bulgarein (**5**) is an example of such a benzo[*j*]fluoranthene-based natural product, which was discovered to induce topoisomerase I-mediated DNA cleavage [16–17].

**Scheme 1:**
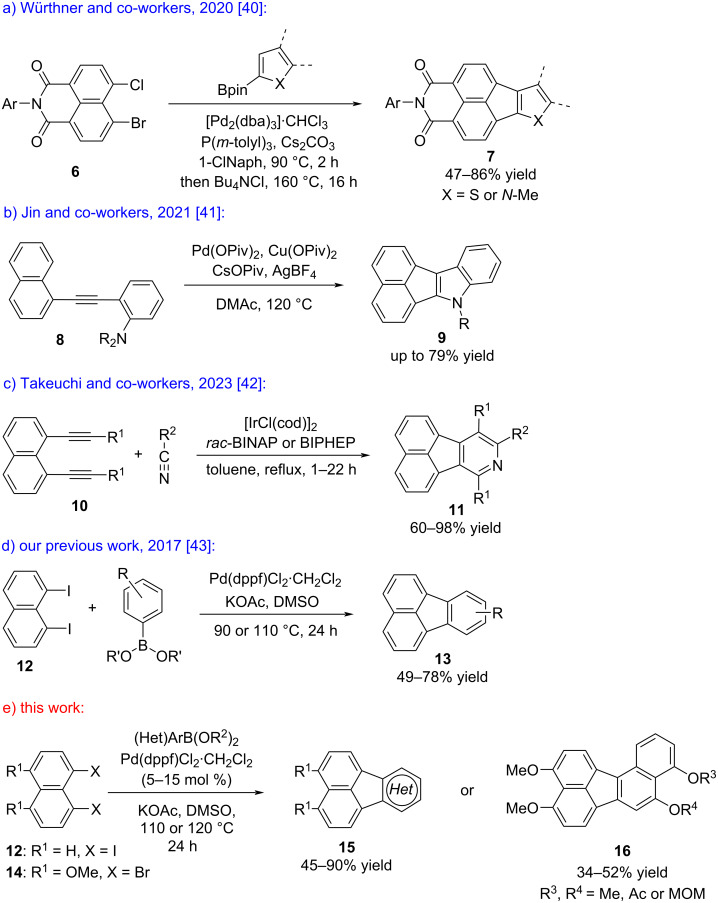
Selected synthetic strategies towards heterocyclic fluoranthene analogues, and our approach.

For the construction of the fluoranthene skeleton, a broad range of synthetic strategies including C–H arylation [18–22], Diels–Alder [7–8,23–25] and [2 + 2 + 2] cycloadditions [26–27], as well as Friedel–Crafts [28] and Prins-type [29] reactions have been developed to date [2]. However, methods that enable access to the analogous acenaphthylene-fused heteroarenes are less common [30–39]. In one such study, Würthner and co-workers reported a Pd-catalyzed annulation reaction between bromo-chloronaphthalene dicarboximides **6** and heteroarylboronic esters that enabled the syntheses of acenaphthylene-fused thiophene and indole derivatives **7** having donor-acceptor units (Scheme 1a) [40]. In 2021, Jin and co-workers developed an elegant Pd-catalyzed reaction cascade starting from diarylalkynes **8**, which involves indole formation/*peri*-C–H annulation and *N*-dealkylation reactions to afford acenaphthylene-fused indole products **9** (Scheme 1b) [41]. Recently, Takeuchi and co-workers reported an effective Ir-catalyzed [2 + 2 + 2] cycloaddition between 1,8-dialkynylnaphthalenes **10** and nitriles that gives rise to the formation of multi-substituted azafluoranthenes **11** in high yields (Scheme 1c) [42]. Contemporaneously, in their work on the synthesis of fluoranthenes, Nagashima, Tanaka and co-workers demonstrated the use of methyl cyanoformate and isocyanates in Rh(I)-catalyzed [2 + 2 + 2] cycloaddition reactions with 1,8-dialkynylnaphthalenes to access azafluoranthenes and 2-pyridone-fused naphthalenes [27]. In 2017, we reported a Pd-catalyzed cascade reaction that involves a sequential Suzuki–Miyaura cross-coupling and a subsequent intramolecular C–H arylation between 1,8-diiodonaphthalene (**12**) and a broad range of arylboronic acids and esters to afford substituted fluoranthenes **13** in good to high yields (Scheme 1d) [43]. In that work, we had only one example of a heterocyclic fluoranthene analogue where the use of 4-pyridylboronic acid provided the corresponding azafluoranthene product [43]. In the current work, we report a full account of our studies on the application of the fluoranthene synthesis methodology to a wide range of acenaphthylene-fused heteroarenes (Scheme 1e). Moreover, our method enables the modular syntheses of various differentially protected, polyoxygenated benzo[*j*]fluoranthenes **16** in an efficient manner, which all have structural relevance to benzo[*j*]fluoranthene-based fungal natural products.

## Results and Discussion

We started our studies on the synthesis of heterocyclic fluoranthene analogues by investigating the reaction between 1,8-diiodonaphthalene (**12**) and thiophene-3-ylboronic acid and ester derivatives **17** under the optimized conditions reported in our previous work (Table 1) [43]. Gratifyingly, the reaction worked smoothly with the use of thiophene-3-ylboronic acid (**17a**) to give acenaphtho[1,2-*b*]thiophene (**15a**) in 76% yield when Pd(dppf)Cl_2_·CH_2_Cl_2_ was used with 5 mol % catalyst loading (Table 1, entry 1). Next, we examined whether different thiophene-3-ylboronic esters could also be used under the same reaction conditions. A variety of borylation methods are capable of providing different boronic esters, such as pinacol [44–46] or catechol [47] boronic esters as the final products. Therefore, it is important that they can be directly utilized with our methodology without further boronic ester interconversions. To this end, we first tested the reaction of thiophene-3-ylboronic acid pinacol ester (**17b**), and we were pleased to see that the desired product **15a** was isolated with a similar reaction yield (78%, Table 1, entry 2). Thiophene-3-ylboronic acid catechol ester (**17c**) was also found to be a competent reaction partner affording the final product **15a** successfully, albeit in a slightly lower yield (69%, Table 1, entry 3). Finally, due to our interest in the structural features and chemistry of 1,8-dihydroxynaphthalene (1,8-DHN) [48–49], we were curious to check the reactivity of the previously unknown boronic ester **17d**, which was prepared in one step from thiophene-3-ylboronic acid (**17a**) and 1,8-DHN [50]. Note that boronic esters of 1,8-DHN have recently been investigated and reported by Krempner and co-workers [51]. To our delight, the Suzuki–Miyaura coupling/intramolecular C–H arylation sequence between **12** and boronic ester **17d** proceeded smoothly affording product **15a** in 84% yield. The results summarized in Table 1 demonstrate that a variety of thiopheneboronic acid and esters are competent substrates in our fluoranthene synthesis methodology.

**Table 1:** Investigation of different thiophene-3-ylboronic acid and esters in the synthesis of **15a**.

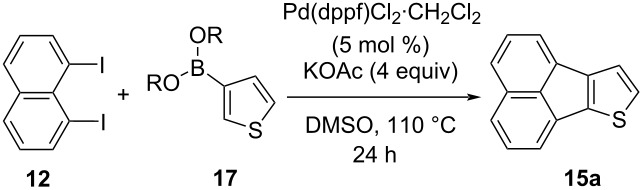		

entry	boronic acid/ester	yield (%)

1	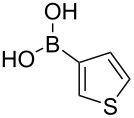 **17a**	76
2	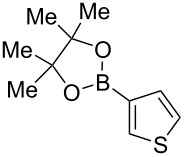 **17b**	78
3	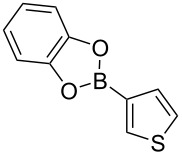 **17c**	69
4	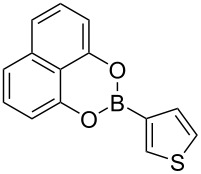 **17d**	84

Following the successful results with the use of thiophene boronic acid and esters, we next investigated the scope of our methodology to prepare a variety of other heterocyclic frameworks (Table 2). The acenaphthylene-fused furan and benzofuran products **15b** and **15c** were obtained in 54% and 86% yield, respectively, via the reactions of 1,8-diiodonaphthalene (**12**) with 3-furanylboronic acid and 2-benzofuranylboronic acid (Table 2, entries 1 and 2). It is important to note that benzofuran adduct **15c** was previously reported by Dyker to be obtained in only 10% yield when **12** was reacted with 10 equivalents of unsubstituted benzofuran, in the presence of 5 mol % of Pd(OAc)_2_ at 100 °C for 3 days [52]. The compatibility of five-membered nitrogen heterocycles with our methodology was further examined with (1-methyl-1*H*-pyrazole-5-yl)boronic acid pinacol ester as the pyrazole ring is one of the most frequently occurring aromatic nitrogen heterocycles among the pharmaceuticals approved by the U.S. FDA between 2013 and 2023 according to a recently-reported analysis [53]. With the use of this boronic ester, acenaphthylene-fused pyrazole product **15d** was isolated in 80% yield (Table 2, entry 3). Importantly, the use of 1,8-dibromo-4,5-dimethoxynaphthalene (**14**) with the same boronic ester afforded dimethoxy-substituted acenaphthylene-fused pyrazole **15e** in good yield (73%, Table 2, entry 4). Afterwards, we sought to examine the reactivity of six-membered aromatic nitrogen heterocycles. The reactions of (2-methoxypyridin-3-yl)boronic acid with the dihalonaphthalenes **12** and **14** afforded substituted azafluoranthenes **15f** and **15g** in 90 and 51% yields, respectively (Table 2, entries 5 and 6). As aforementioned, in our previous work, we had reported the use of 4-pyridylboronic acid as the only heterocycle in our fluoranthene synthesis methodology, and azafluoranthene **15h** was obtained in that study as the only possible regioisomer [43]. In the current work, we opted to check the regioselectivity of the reaction when 3-pyridylboronic acid is used as substrate as there are two different positions (C2 and C4) on the pyridine ring available for the C–H arylation step. Interestingly, azafluoranthene **15h** was obtained as the only product in this reaction, albeit in a lower yield (45%, Table 2, entry 7). The reaction with the symmetrical pyrimidine-5-ylboronic acid was observed to proceed smoothly to give acenaphthylene-fused pyrimidine **15i** in 74% yield (Table 2, entry 8). After the completion of our studies on heterocyclic fluoranthene analogues, our next target was the synthesis of highly electron-rich benzo[*j*]fluoranthenes. However, before moving to this compound class, we wanted to confirm the success of our methodology on a model electron-rich substrate. For this purpose, we checked the reaction between 1,8-diiodonaphthalene (**12**) and 2,3-dimethoxyphenylboronic acid, and we were happy to see that electron-rich fluoranthene **15j** was isolated in 76% yield (Table 2, entry 9).

**Table 2:** Investigation of various (hetero)arylboronic acids and esters in the Suzuki–Miyaura/C–H arylation sequence.

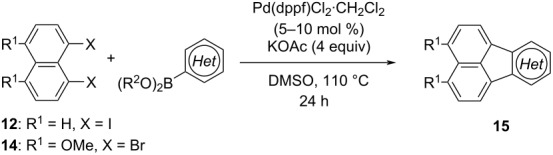				

entry	dihalonaphthalene	ArB(OR^2^)_2_	product	yield (%)

1^a^	**12**	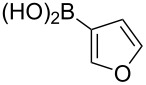	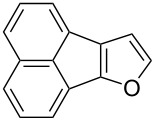 **15b**	54
2^b^	**12**	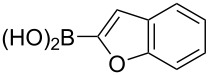	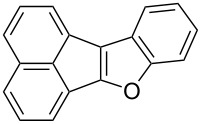 **15c**	86
3^a^	**12**	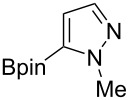	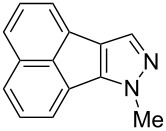 **15d**	80
4^b^	**14**	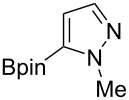	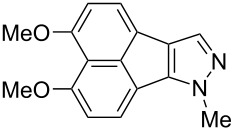 **15e**	73
5^a^	**12**	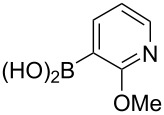	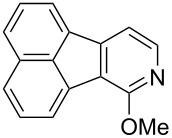 **15f**	90
6^b^	**14**	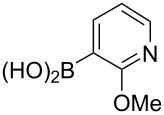	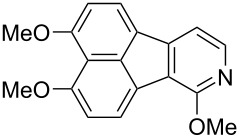 **15g**	51
7^a^	**12**	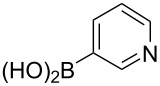	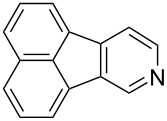 **15h**	45
8^a^	**12**	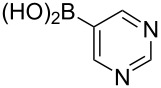	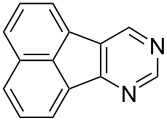 **15i**	74
9^a^	**12**	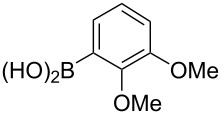	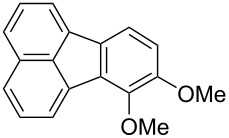 **15j**	76

^a^5 mol % of Pd(dppf)Cl_2_·CH_2_Cl_2_ was used. ^b^10 mol % of Pd(dppf)Cl_2_·CH_2_Cl_2_ was used.

**Scheme 2:**
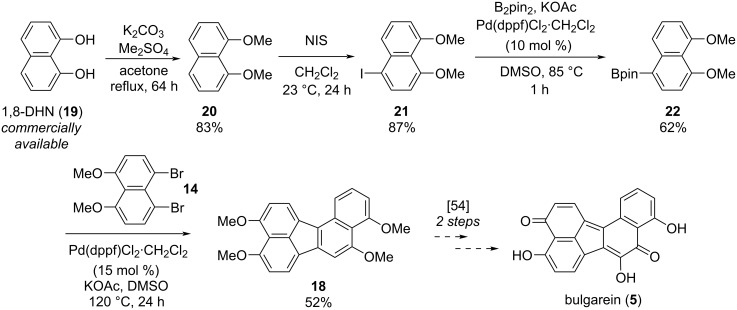
Synthesis of benzo[*j*]fluoranthene **18**.

In the next phase of our studies, we turned our attention to the construction of polyoxygenated benzo[*j*]fluoranthenes, which represent the core skeleton of many fungal natural products [15]. For this purpose, we first concentrated our efforts on the synthesis of highly electron-rich benzo[*j*]fluoranthene **18** possessing four methoxy groups (Scheme 2). This compound was previously synthesized by Swieca and Spiteller in six steps (longest linear sequence, LLS) starting from 1,5-dihydroxynaphthalene [54]. It was also shown by the authors that compound **18** could be efficiently converted to the fungal natural product bulgarein (**5**) in only two steps. Our synthesis started with the mono-iodination of 1,8-dimethoxynaphthalene (**20**), which was prepared in single step from the commercially available 1,8-dihydroxynaphthalene (1,8-DHN, **19**) [48,55], by *N*-iodosuccinimide (NIS) in 87% yield (Scheme 2). Afterwards, naphthylboronic ester **22** was obtained via the Miyaura borylation [44] of iodonaphthalene **21** in 62% yield. Whereas this compound acts as the boronic ester coupling partner of our fluoranthene synthesis methodology, the previously known naphthalene dibromide **14** was prepared in two steps from 1,8-DHN (**19**) [56] serving as the second coupling partner. The Pd-catalyzed cascade reaction between **14** and **22** afforded the targeted benzo[*j*]fluoranthene **18** in 52% yield. Overall, the synthesis of **18**, which was used as the key precursor in the synthesis of bulgarein (**5**) [54], has been synthesized efficiently via a four-step sequence (LLS).

**Scheme 3:**
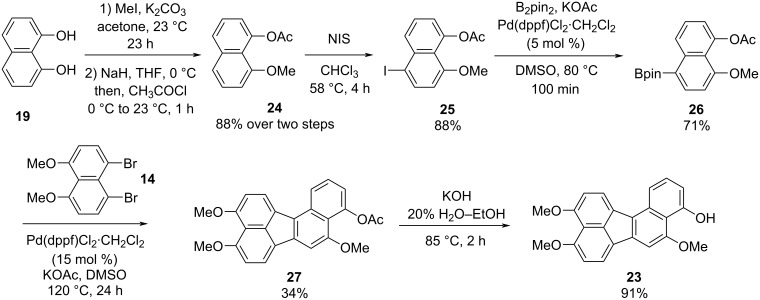
Synthesis of benzo[*j*]fluoranthene **23**.

The synthesis of benzo[*j*]fluoranthene **18** described above underscores that our fluoranthene synthesis methodology could successfully be applied to construct highly oxygenated and multi-substituted benzo[*j*]fluoranthene derivatives, which have the potential to be useful in natural product synthesis. However, all oxygens in compound **18** are protected as methyl ethers, and therefore, demethylating one or some of these methoxy groups selectively is anticipated to be extremely challenging, if not impossible. In order to overcome this problem, we opted to synthesize benzo[*j*]fluoranthene analogues with differentially protected oxygens so that specific positions can be functionalized further if needed after selective deprotection. To this end, first we accomplished an efficient synthesis of benzo[*j*]fluoranthene **23** in six steps (Scheme 3). In this sequence, 1,8-DHN (**19**) was first converted to 1-acetoxy-8-methoxynaphthalene (**24**) via a selective mono-methylation of **19** followed by acetylation by acetyl chloride in 88% yield over two steps. The more electron-rich ring of naphthalene **24** was selectively iodinated from the *para*-position with respect to the -OMe group with the use of NIS to afford iodonaphthalene **25** in 88% yield. A subsequent Miyaura borylation of **25** using B_2_pin_2_ under Pd catalysis gave boronic ester **26** in 71% yield, which set the stage for the key fluoranthene formation reaction. Pleasingly, benzo[*j*]fluoranthene **27** was obtained successfully via the Pd-catalyzed reaction of dibromonaphthalene **14** and naphthylboronic ester **26** in DMSO at 120 °C, albeit in a modest yield of 34%. A final basic hydrolysis of the acetoxy group furnished benzo[*j*]fluoranthene **23** bearing a free naphthol group in high yield (91%).

**Scheme 4:**
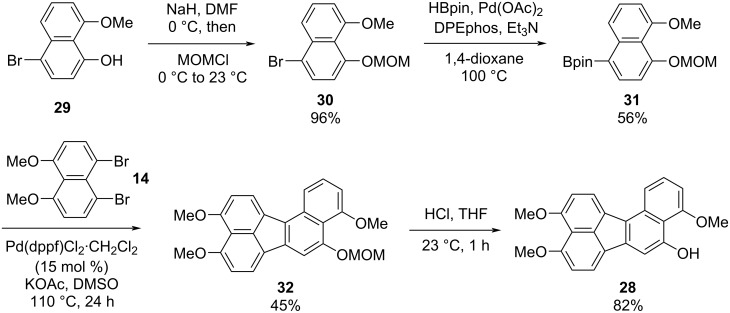
Synthesis of benzo[*j*]fluoranthene **28**.

We next turned our attention to the synthesis of benzo[*j*]fluoranthene **28**, which is structurally related to **23**, but with a free -OH group at a different position (Scheme 4). Our synthetic sequence commenced with the preparation of the known bromonaphthol **29** in two steps from 1,8-DHN (**19**) [57]. Methoxymethyl (MOM) protection of free -OH group of **29** using NaH and MOMCl afforded MOM-protected naphthol **30** in excellent yield (96%) [58–59]. It is worth highlighting the structural differences of naphthalenes **25** and **30**, where the halogen (iodine) is on the same ring with the -OMe group on **25**, while the halogen (bromine) is on the opposite ring with the -OMe group on naphthalene **30**. This small yet important difference allowed us to synthesize benzo[*j*]fluoranthenes **23** and **28** selectively with free -OH groups at different positions. Bromonaphthalene **30** was subsequently subjected to Pd-catalyzed borylation conditions reported by Colobert and co-workers [60] with HBpin as the borylating agent to give naphthylboronic ester **31** in 56% yield (Scheme 4). The fluoranthene formation protocol was employed for the reaction between ArBpin **31** and dibromonaphthalene **14**, and the targeted benzo[*j*]fluoranthene product **32** was isolated in 45% yield. It should be noted that this reaction was not successful when an analogue of compound **31** with -OTIPS instead of the -OMOM group was tested as the boronic ester reaction partner, due to the decomposition of this compound presumably via the deprotection of the -OTIPS silyl ether under the reaction conditions. A final MOM-deprotection under acidic conditions led to the formation of the desired benzo[*j*]fluoranthene **28** in 82% yield.

## Conclusion

In conclusion, we have demonstrated the successful synthesis of heterocyclic fluoranthene analogues via a Pd-catalyzed reaction cascade that consists of a Suzuki–Miyaura cross-coupling reaction followed by an intramolecular C–H arylation. These heterocyclic fluoranthene analogues include a variety of acenaphthylene-fused heteroarenes such as thiophene, furan, benzofuran, pyrazole, pyridine and pyrimidine derivatives, which were obtained in good to high yields (45–90%). Notably, we have shown that both boronic acid and a range of boronic esters could be utilized as reaction partners with comparable effectiveness in our methodology. The fluoranthene formation method was also applied to construct highly electron-rich, polyoxygenated benzo[*j*]fluoranthenes, which are all structurally relevant to benzo[*j*]fluoranthene-based fungal natural products. In this respect, it is important to mention that the synthesis of the tetramethoxy-substituted benzo[*j*]fluoranthene **18** in four steps represents a concise formal total synthesis of bulgarein (**5**) [54]. A careful design of the synthetic sequences enabled the syntheses of benzo[*j*]fluoranthenes **27** and **32** with differentially protected naphthol groups at different positions, which provides potential opportunities for further structural diversification.

## Supporting Information

File 1 Experimental procedures, characterization data, and copies of ^1^H and ^13^C{^1^H} NMR spectra.

## Data Availability

All data that supports the findings of this study is available in the published article and/or the supporting information of this article.
