# Supplementary material for: Synthesis of acenaphthylene-fused heteroarenes and polyoxygenated benzo[j]fluoranthenes via a Pd-catalyzed Suzuki–Miyaura/C–H arylation cascade
Source: Beilstein J Org Chem. 2024 Dec 23;20:3290–8. doi: 10.3762/bjoc.20.273 (PMC11693970; doi:10.3762/bjoc.20.273)
Supplement: File 1 — Experimental procedures, characterization data, and copies of 1H and 13C{1H} NMR spectra. [file Beilstein_J_Org_Chem-20-3290-s001.pdf]

BEILSTEIN JOURNAL OF ORGANIC CHEMISTRY

Supporting Information

for

Synthesis of acenaphthylene-fused heteroarenes and polyoxygenated benzo[*j*]fluoranthenes via a Pd-catalyzed Suzuki–Miyaura/C–H arylation cascade

Merve Yence, Dilgam Ahmadli, Damla Surmeli, Umut Mert Karacaoğlu, Sujit Pal and Yunus Emre Türkmen

Beilstein J. Org. Chem. **2024,** *20,* 3290–3298. doi:10.3762/bjoc.20.273

Experimental procedures, characterization data, and copies of ^{1}H and $^{13}C\{^{1}H\}$ NMR spectra

License and Terms: This is a supporting information file under the terms of the Creative Commons Attribution License (https://creativecommons.org/licenses/by/4.0). Please note that the reuse, redistribution and reproduction in particular requires that the author(s) and source are credited and that individual graphics may be subject to special legal provisions.
The license is subject to the *Beilstein Journal of Organic Chemistry* terms and conditions: (https://www.beilstein-journals.org/bjoc/terms)

Table of Contents

S2 General information and materials

S3 Synthesis of acenaphthylene-fused heteroarenes

S11 Synthesis of benzo[*j*]fluoranthene derivatives

S26 ^{1}H and $^{13}C\{^{1}H\}$ NMR spectra

General information: All air sensitive reactions were performed using oven-dried glassware under an inert atmosphere of nitrogen. Solvents for Pd-catalyzed reactions were deoxygenated prior to the reactions by purging with nitrogen for five minutes. Aluminum-backed plates pre-coated with silica gel (Merck, Silica Gel 60 F_{254}) were used for monitoring reactions by thin-layer chromatography (TLC). UV light and $KMnO_4$ staining solutions were used for TLC visualization. Flash column chromatography was carried out using Silicycle 40–63 μm (230–400 mesh) silica gel. NMR spectra were measured using a Bruker spectrometer at 400 MHz for ^{1}H NMR spectra and 100 MHz for ^{13}C{^{1}H} NMR spectra. The spectra were calibrated with the use of an internal standard (TMS, 0 ppm) or residual solvent signals (chloroform at 7.26 ppm for ^{1}H NMR spectra, and at 77.16 ppm for ^{13}C{^{1}H} NMR spectra). ^{1}H NMR data are reported as follows: chemical shift (parts per million, ppm), integration, multiplicity (s = singlet, d = doublet, t = triplet, dd = doublet of doublets, m = multiplet, br = broad), coupling constant (Hz). Infrared (FTIR) spectra were recorded on a Bruker Alpha-Platinum-ATR spectrometer, and only selected peaks were reported. An Agilent Technologies 6224 TOF LC/MS instrument was utilized for high resolution mass spectrometry (HRMS) analyses at UNAM-National Nanotechnology Research Center, Institute of Materials Science and Nanotechnology, Bilkent University.

Materials: Anhydrous THF, DMF and CH_2Cl_2 were purchased from *Acros Organics* (*AcroSeal*®). 1,8-Dihydroxynaphthalene (**19**) was purchased from abcr and used as received. *N*-Bromosuccinimide (NBS) was recrystallized from H_2O, dried thoroughly, and stored in refrigerator. All other commercially available reagents were used as received unless stated otherwise.

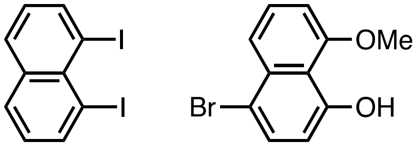

12 **29**

Compounds **12**[1] and **29**[2] were prepared following previously reported procedures.

Synthesis of acenaphthylene-fused heteroarenes:

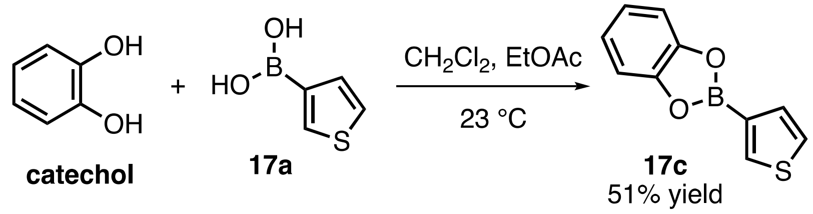

Catechol (86 mg, 0.78 mmol) was added to a solution of thiophene-3-boronic acid (**17a**) (100 mg, 0.78 mmol) in anhydrous CH_2Cl_2 (2.6 mL). Ethyl acetate was added dropwise until a homogeneous solution was obtained. The resulting solution was stirred overnight at 23 °C. Afterwards, anhydrous Na_2SO_4 was added, and the resulting mixture was filtered and washed with EtOAc. The resulting solution was concentrated under vacuum to give a pale yellow solid. Recrystallization by slow evaporation from a CH_2Cl_2 solution afforded pure boronic ester **17c** as white crystals (81 mg, 51% yield).

^{1}H NMR (400 MHz; CDCl$_3$) δ: 8.26 (1H, dd, *J* = 2.7, 1.0 Hz), 7.68 (1H, dd, *J* = 4.9, 1.1 Hz), 7.48 (1H, dd, *J* = 4.8, 2.7 Hz), 7.36-7.29 (2H, m), 7.16-7.12 (2H, m).
^{13}C{^{1}H} NMR (100 MHz; CDCl$_3$) δ: 148.5, 138.3, 131.9, 126.4, 122.9, 112.7
FTIR ν_{max} (ATR, solid)/cm^{-1} 3091, 2923, 2853, 1521, 1470, 1413, 1300, 1233
HRMS (-APCI) Calcd for $C_{11}H_{10}BO_3S$ [M+OCH$_3$]$^-$ 233.0449, found: 233.0403

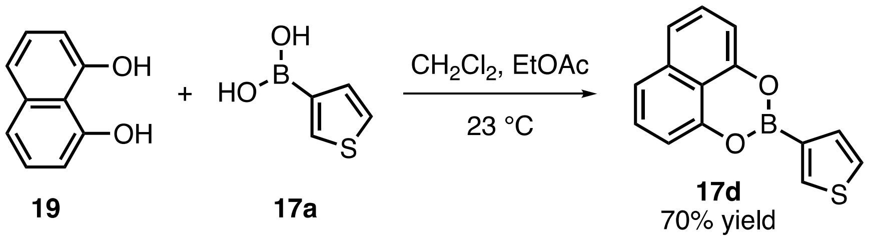

1,8-DHN (**19**) (126 mg, 0.78 mmol) was added to a solution of thiophene-3-boronic acid (**17a**) (100 mg, 0.78 mmol) in anhydrous CH_2Cl_2 (2.6 mL). Ethyl acetate was added dropwise until a homogeneous solution was obtained. The resulting solution was stirred overnight at 23 °C. Afterwards, anhydrous Na_2SO_4 was added, and the resulting mixture was filtered and washed with EtOAc. The resulting solution was concentrated

under vacuum to give a pale yellow solid. Recrystallization by slow evaporation from a CH_2Cl_2 solution afforded pure boronic ester **17d** as white crystals (138 mg, 70% yield).

^{1}H NMR (400 MHz; $CDCl_3$) δ: 8.25 (1H, dd, *J* = 2.7, 1.0 Hz), 7.67 (1H, dd, *J* = 4.9, 1.1 Hz), 7.45-7.42 (3H, m), 7.38 (1H, d, *J* = 8.4 Hz), 7.36 (1H, d, *J* = 8.4 Hz), 6.97-6.95 (2H, m).

^{13}C{^{1}H} NMR (100 MHz; $CDCl_3$) δ: 147.9, 138.1, 135.3, 132.0, 127.9, 125.8, 121.1, 117.7, 109.6.

FTIR ν_{max} (ATR, solid)/cm^{-1} 1633, 1609, 1517, 1409, 1371, 1296, 1277.

HRMS (+APCI) Calcd for $C_{14}H_{10}BO_2S$ $[M+H]^+$ 253.0490, found: 253.0491.

General procedure for the synthesis of acenaphthylene-fused heteroarenes:

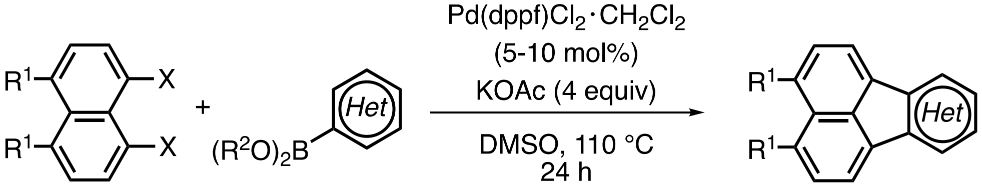

12: R^1 = H, X = I
14: R^1 = OMe, X = Br
15

A 25-mL, oven-dried, round-bottomed flask was charged with DMSO (2 mL) under nitrogen atmosphere. The solution was deoxygenated by bubbling nitrogen gas through the solution for 5 min. Dihalonaphthalene (1.0 equiv), arylboronic acid or ester (1.1 equiv), $Pd(dppf)Cl_2 \cdot CH_2Cl_2$ (5 or 10 mol %) and KOAc (4 equiv) were added sequentially to the solution. The flask was then sealed with a glass stopper, and the reaction mixture was stirred at 110 ºC for 24 h. The progress of the reaction was checked by TLC. After cooling to room temperature, brine (ca. 10 mL) was added to the reaction mixture, and the aqueous phase was extracted with EtOAc (2 × 10 mL). The combined organic phase was dried over Na_2SO_4, filtered, and concentrated under vacuum. The viscous crude product was purified by flash column chromatography (SiO_2) to give the desired product.

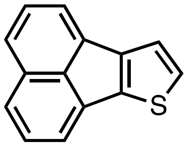

15a

Compound **15a** was prepared according to the General Procedure using 1,8-diiodonaphthalene (**12**) (50 mg, 0.13 mmol), thiophene-3-boronic acid (18.5 mg, 0.145 mmol), Pd(dppf)Cl_2·CH_2Cl_2 (5.4 mg, 0.006 mmol) and KOAc (52 mg, 0.53 mmol). Purification by flash column chromatography (only hexanes) gave **15a** as a yellow solid (20.9 mg, 76% yield).

Mp = 69.7-70.5 °C
***R*$_f$** = 0.50 (only hexanes)
^{1}H NMR (400 MHz; $CDCl_3$) δ: 7.76-7.71 (4H, m), 7.56-7.52 (2H, m), 7.41 (1H, d, *J* = 4.8 Hz), 7.37 (1H, d, *J* = 4.9 Hz).
^{13}C{^{1}H} NMR (100 MHz; $CDCl_3$) δ: 145.9, 141.6, 134.0, 133.8, 133.6, 129.6, 128.4, 127.80, 127.78, 126.6, 126.3, 120.9, 120.5, 120.2.
FTIR ν_{max} (ATR, solid)/cm^{-1} 2922, 2851, 1475, 1431, 1408, 1370, 1185, 1119, 1083.
HRMS (+APCI) Calcd for $C_{14}H_9S$ $[M+H]^+$ 209.0420, found: 209.0430.

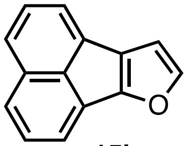

15b

Compound **15b** was prepared according to the General Procedure using 1,8-diiodonaphthalene (**12**) (50 mg, 0.13 mmol), 3-furanylboronic acid (16 mg, 0.15 mmol), Pd(dppf)Cl_2·CH_2Cl_2 (5.4 mg, 0.006 mmol) and KOAc (52 mg, 0.53 mmol). Purification by flash column chromatography (only hexanes) gave **15b** as a dark brown oil (13.6 mg, 54% yield).

***R*$_f$** = 0.76 (only hexanes)

^{1}H NMR (400 MHz; CDCl$_3$) δ: 7.72 (1H, dd, *J* = 4.1, 0.5 Hz), 7.71-7.68 (2H, m), 7.64 (1H, dd, *J* = 6.8, 0.6 Hz), 7.54-7.48 (3H, m), 6.78 (1H, d, *J* = 2.0 Hz).

^{13}C{^{1}H} NMR (100 MHz; CDCl$_3$) δ: 147.0, 143.0, 131.5, 130.7, 129.5, 127.7, 127.6, 127.4, 127.02, 126.97, 126.6, 121.9, 119.2, 106.9.

FTIR ν_{max} (ATR, film)/cm^{-1} 3049, 2921, 2851, 1708, 1664, 1478, 1466, 1434.

HRMS (+APCI) Calcd for $C_{14}H_9O$ [M+H]$^+$ 193.0648, found: 193.0639.

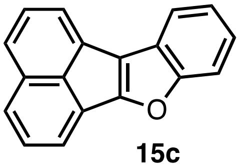

15c

Compound **15c** was prepared according to the General Procedure using 1,8-diiodonaphthalene (**12**) (50 mg, 0.13 mmol), 2-benzofuranylboronic acid (24 mg, 0.15 mmol), Pd(dppf)Cl_2·CH_2Cl_2 (10.8 mg, 0.013 mmol) and KOAc (52 mg, 0.53 mmol). Purification by flash column chromatography (only hexanes) gave **15c** as an orange solid (27.5 mg, 86% yield). The ^{1}H NMR spectral data of compound **15c** match the reported data in the literature.[3]

Mp = 78.7-80.0 °C

R_f = 0.55 (only hexanes)

^{1}H NMR (400 MHz; CDCl$_3$) δ: 7.85-7.80 (4H, m), 7.74 (1H, d, *J* = 8.3 Hz), 7.62-7.54 (3H, m), 7.37-7.29 (2H, m).

FTIR ν_{max} (ATR, solid)/cm^{-1} 3051, 2923, 2852, 1708, 1665, 1604, 1571, 1478, 1467, 1435.

HRMS (+APCI) Calcd for $C_{18}H_{11}O$ [M+H]$^+$ 243.0805, found: 243.0812.

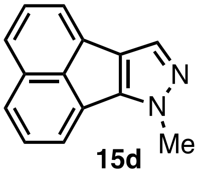

15d

Compound **15d** was prepared according to the General Procedure using 1,8-diiodonaphthalene (**12**) (50 mg, 0.13 mmol), 1-Methyl-1*H*-pyrazole-5-boronic acid

pinacol ester (30 mg, 0.15 mmol), Pd(dppf)$Cl_2 \cdot CH_2Cl_2$ (5.4 mg, 0.006 mmol) and KOAc (52 mg, 0.53 mmol). Purification by flash column chromatography (EtOAc:hexanes = 1:1) gave **15d** as a yellow solid (22 mg, 80% yield).

Mp = 113.2-114.3 °C
R

R_f = 0.68 (EtOAc:hexanes = 1:1)
^{1}H NMR (400 MHz; $CDCl_3$) δ: 7.79 (1H, dd, *J* = 8.2, 0.4 Hz), 7.68-7.66 (2H, m), 7.65 (1H, s), 7.62 (1H, dd, *J* = 6.8, 0.6 Hz), 7.54 (1H, dd, *J* = 8.2, 7.0 Hz), 7.51 (1H, dd, *J* = 8.2, 6.9 Hz), 4.14 (3H, s).
^{13}C{^{1}H} NMR (100 MHz; $CDCl_3$) δ: 148.3, 134.8, 131.8, 131.0, 130.5, 128.2, 127.7, 127.10, 127.05, 126.7, 125.1, 120.9, 119.7, 38.1.
FTIR ν_{max} (ATR, solid)/cm^{-1} 2925, 2851, 1616, 1549, 1478, 1411.
HRMS (+APCI) Calcd for $C_{14}H_{11}N_2$ $[M+H]^+$ 207.0917, found: 207.0928.

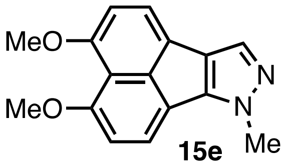

15e

Compound **15e** was prepared according to the General Procedure using 1,8-dibromo-4,5-dimethoxynaphthalene (**14**) (50 mg, 0.145 mmol), 1-Methyl-1*H*-pyrazole-5-boronic acid pinacol ester (33 mg, 0.16 mmol), Pd(dppf)$Cl_2 \cdot CH_2Cl_2$ (11.8 mg, 0.014 mmol) and KOAc (57 mg, 0.58 mmol). Purification by flash column chromatography (EtOAc:hexanes = 1:1 → 2:1 → only EtOAc) gave **15e** as a yellow solid (28 mg, 73% yield).

Mp = 241-244°C (decomposition)
R_f = 0.42 (only EtOAc)
^{1}H NMR (400 MHz; $CDCl_3$) δ: 7.64 (1H, d, *J* = 7.8 Hz), 7.58 (1H, s), 7.56 (1H, d, *J* = 7.8 Hz), 6.87 (1H, d, *J* = 7.9 Hz), 6.83 (1H, d, *J* = 7.8 Hz), 4.13 (3H, s), 4.05 (3H, s), 4.02 (3H, s).

$^{13}C\{^1H\}$ NMR (100 MHz; $CDCl_3$) δ: 158.6, 156.5, 147.2, 137.9, 130.6, 125.9, 123.2, 122.1, 121.2, 119.2, 115.7, 106.7, 106.0, 56.6, 56.5, 37.9

FTIR ν_{max} (ATR, solid)/cm^{-1} 2921, 2838, 1592, 1552, 1454, 1417, 1262, 1244.

HRMS (+APCI) Calcd for $C_{16}H_{15}N_2O_2$ $[M+H]^+$ 267.1129, found: 267.1138.

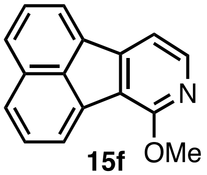

15f

Compound **15f** was prepared according to the General Procedure using 1,8-diiodonaphthalene (**12**) (50 mg, 0.13 mmol), 2-methoxypyridine-3-boronic acid (22 mg, 0.15 mmol), $Pd(dppf)Cl_2 \cdot CH_2Cl_2$ (5.4 mg, 0.006 mmol) and KOAc (52 mg, 0.53 mmol). Purification by flash column chromatography (EtOAc:hexanes = 1:9) gave **15f** as a yellow solid in 90% yield (28 mg).

M.P. = 123.3-123.9 °C

$\boldsymbol{R_f}$ = 0.61 (EtOAc:hexanes = 1:9)

^1H NMR (400 MHz; $CDCl_3$) δ: 8.24 (1H, d, *J* = 5.2 Hz), 8.14 (1H, d, *J* = 6.9 Hz), 8.02 (1H, d, *J* = 7.0 Hz), 7.96 (1H, d, *J* = 8.2 Hz), 7.84 (1H, d, *J* = 8.3 Hz), 7.68-7.64 (2H, m), 7.47 (1H, d, *J* = 5.2 Hz), 4.22 (3H, s).

$^{13}C\{^1H\}$ NMR (100 MHz; $CDCl_3$) δ: 160.5, 149.0, 146.3, 135.2, 134.6, 131.7, 129.9, 129.3, 128.7, 127.8, 126.7, 124.2, 122.8, 120.3, 111.1, 53.6.

FTIR ν_{max} (ATR, solid)/cm^{-1} 2941, 2890, 2851, 1602, 1555, 1422, 1410, 1355.

HRMS (+APCI) Calcd for $C_{16}H_{12}NO$ $[M+H]^+$ 234.0914, found: 234.0928.

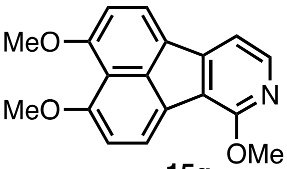

15g

Compound **15g** was prepared according to the General Procedure using 1,8-dibromo-4,5-dimethoxynaphthalene (**14**) (50 mg, 0.145 mmol), 2-methoxypyridine-3-boronic

acid (24 mg, 0.16 mmol), Pd(dppf)Cl_2·CH_2Cl_2 (11.8 mg, 0.014 mmol) and KOAc (57 mg, 0.58 mmol). Purification by flash column chromatography (EtOAc:hexanes = 1:4 → 1:2 →1:1) gave **15g** as a yellow solid (24 mg, 51% (56% yield with 5% impurity)).

R_f = 0.77 (EtOAc:hexanes = 1:1)

^{1}H NMR (400 MHz; $CDCl_3$) δ: 8.14 (1H, d, *J* = 5.2 Hz), 8.11 (1H, d, *J* = 7.8 Hz), 8.00 (1H, d, *J* = 7.8 Hz), 7.42 (1H, d, *J* = 5.2 Hz), 6.98 (1H, d, *J* = 8.0 Hz), 6.97 (1H, d, *J* = 7.6 Hz), 4.20 (3H, s), 4.09 (3H, s), 4.08 (3H, s).

^{13}C{^{1}H} NMR (100 MHz; $CDCl_3$) δ: 160.1, 159.9, 158.1, 147.6, 144.3, 134.8, 127.1, 126.5, 125.9, 124.7, 119.6, 114.3, 110.3, 107.2, 106.8, 56.63, 56.57, 53.5.

FTIR ν_{max} (ATR, solid)/cm^{-1} 2940, 2835, 1597, 1556, 1500, 1450, 1423.

HRMS (+APCI) Calcd for $C_{18}H_{16}NO_3$ $[M+H]^+$ 294.1125, found: 294.1140.

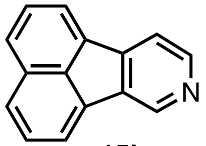

15h

Compound **15h** was prepared according to the General Procedure using 1,8-diiodonaphthalene (**12**) (50 mg, 0.13 mmol), pyridine-3-boronic acid (18 mg, 0.15 mmol), Pd(dppf)Cl_2·CH_2Cl_2 (5.4 mg, 0.006 mmol) and KOAc (52 mg, 0.53 mmol). Purification by flash column chromatography (EtOAc:hexanes = 1:3 → 1:2 → 1:1) gave azafluoranthene **15h** as a green solid (11.9 mg, 45% yield). The spectral data of compound **15h** match the reported data in the literature.[4]

R_f = 0.35 (only EtOAc)

^{1}H NMR (400 MHz; $CDCl_3$) δ: 9.19 (1H, s), 8.66 (1H, d, *J* = 5.0 Hz), 8.09 (1H, d, *J* = 6.9 Hz), 8.06 (1H, d, *J* = 6.9 Hz), 8.01 (1H, d, *J* = 8.2 Hz), 7.93 (1H, d, *J* = 8.2 Hz), 7.83 (1H, dd, *J* = 5.0, 0.9 Hz), 7.76-7.66 (2H, m).

FTIR ν_{max} (ATR, solid)/cm^{-1} 3040, 2923, 2852, 1602, 1556, 1454, 1425.

HRMS (+APCI) Calcd for $C_{15}H_{10}N$ $[M+H]^+$ 204.0808, found: 204.0816.

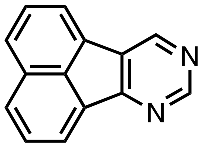

15i

Compound **15i** was prepared according to the General Procedure using 1,8-diiodonaphthalene (**12**) (30 mg, 0.079 mmol), pyrimidine-5-boronic acid (10.8 mg, 0.087 mmol), Pd(dppf)Cl_2·CH_2Cl_2 (3.2 mg, 0.0040 mmol) and KOAc (31 mg, 0.32 mmol). After all reactants were added at 23 °C, the mixture was gradually heated to 110 °C, and stirred at this temperature for 24 h. Purification by flash column chromatography (EtOAc:hexanes = 1:3 → 1:2 → 1:1 → only EtOAc) gave fluoranthene **15i** as a brown solid (11.9 mg, 74% yield).

R_f = 0.23 (EtOAc:hexanes = 1:3)

^{1}H NMR (400 MHz; CDCl$_3$) δ: 9.24 (1H, s), 9.21 (1H, s), 8.41 (1H, d, *J* = 6.9 Hz), 8.14 (1H, d, *J* = 8.2 Hz), 8.08 (1H, d, *J* = 6.9 Hz), 8.03 (1H, d, *J* = 8.3 Hz), 7.83 (1H, dd, *J* = 8.2, 6.9 Hz), 7.74 (1H, dd, *J* = 8.3, 7.0 Hz).

^{13}C{^{1}H} NMR (100 MHz; CDCl$_3$) δ: 165.7, 157.4, 148.6, 133.5, 132.3, 131.23, 131.17, 130.5, 130.1, 128.73, 128.67, 128.5, 124.1, 122.6.

FTIR ν_{max} (ATR, solid)/cm^{-1} 3043, 2960, 2920, 2850, 1581, 1539, 1421, 1370.

HRMS (+APCI) Calcd for $C_{14}H_9N_2$ $[M+H]^+$ 205.0761, found: 205.0772.

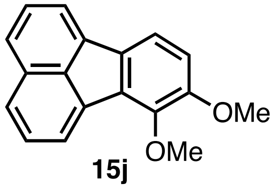

15j

Compound **15j** was prepared according to the General Procedure using 1,8-diiodonaphthalene (**12**) (50 mg, 0.13 mmol), 2,3-dimethoxyphenylboronic acid (26 mg, 0.15 mmol), Pd(dppf)Cl_2·CH_2Cl_2 (5.4 mg, 0.006 mmol) and KOAc (52 mg, 0.53

mmol). Purification by flash column chromatography (EtOAc:hexanes = 1:9) gave fluoranthene **15j** as a yellow solid (26.3 mg, 76% yield).

R_f = 0.45 (EtOAc:hexanes = 1:9)

^{1}H NMR (400 MHz; $CDCl_3$) δ: 8.18 (1H, d, *J* = 6.9 Hz), 7.84-7.82 (2H, m), 7.78 (1H, d, *J* = 8.2 Hz), 7.67-7.58 (3H, m), 6.92 (1H, d, *J* = 8.1 Hz), 4.10 (3H, s), 3.97 (3H, s).

^{13}C{^{1}H} NMR (100 MHz; $CDCl_3$) δ: 153.2, 146.2, 137.1, 135.6, 133.6, 133.0, 132.6, 130.1, 128.3, 127.9, 126.6, 125.7, 123.4, 119.2, 117.4, 111.6, 60.4, 56.5.

FTIR ν_{max} (ATR, solid)/cm^{-1} 2922, 2850, 1496, 1445, 1427, 1416.

HRMS (+APCI) Calcd for $C_{18}H_{15}O_2$ $[M+H]^+$ 263.1067, found: 263.1076.

Synthesis of benzo[*j*]fluoranthene derivatives:

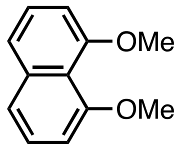

20

A 250 mL round-bottomed flask was charged with 1,8-dihydroxynaphthalene (1,8-DHN, **19**) (2.00 g, 12.5 mmol). 100 mL of acetone was added at 23 ºC to give a brown solution. K_2CO_3 (17.3 gram, 125 mmol) and Me_2SO_4 were added sequentially at 23 ºC. The resulting suspension was heated to 70 ºC and vigorously stirred under reflux at 70–80 ºC for 64 h at the end of which TLC indicated the full consumption of 1,8-DHN. The reaction mixture was then cooled down to 23 ºC, and acetone was removed under reduced pressure. In order to destroy and remove the unreacted excess Me_2SO_4, 100 mL of 4 M aqueous NaOH solution was added to a solution of the remaining solid in CH_2Cl_2 at 23 ºC, and the mixture was stirred at this temperature for 2 h. Layers were partitioned in a separatory funnel, and the aqueous phase was washed three times with CH_2Cl_2. The combined organic phase was dried over anhydrous Na_2SO_4, filtered and concentrated under reduced pressure. Purification by column chromatography (SiO_2; EtOAc:hexanes = 1:19 → 1:9 → 1:5→ 1:1) gave pure **20** (1.96 g, 83% yield) as brownish-yellow solid. The spectral data match the reported data in the literature.[5]

R_f = 0.46 (EtOAc:hexanes = 1:9).

¹H NMR (400 MHz; $CDCl_3$) δ: 7.43-7.33 (4H, m), 6.86 (2H, dd, *J* = 7.2, 1.5 Hz), 3.98 (6H, s).

¹³C{¹H} NMR (100 MHz; $CDCl_3$) δ: 157.2, 137.5, 126.5, 121.0, 119.0, 106.4, 56.6.

FTIR ν_{max} (ATR, solid)/cm^{-1} 1580, 1510, 1460, 1427, 1386, 1348, 1273, 1237, 1180.

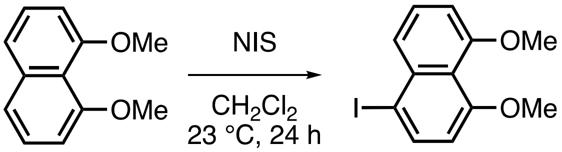

20 **21**

A solution of 1,8-dimethoxynaphthalene (**20**) (400 mg, 2.13 mmol) in 7.0 mL of CH_2Cl_2 was cooled to 0 °C using an ice bath. *N*-iodosuccinimide (986 mg, 4.25 mmol) was added in portions, and after 5 min, ice bath was removed. The resulting solution was stirred at 23 °C for 24 h. The reaction mixture was quenched with saturated aqueous $Na_2S_2O_3$ solution and the biphasic mixture was partitioned in a separatory funnel. The organic phase was washed once with water, and then, the combined aqueous phase was extracted once with EtOAc. The combined organic phase was dried over Na_2SO_4, filtered and concentrated under reduced pressure. Purification by column chromatography (EtOAc:hexanes = 1:9) afforded pure iodonaphthalene **21** (584 mg, 87%) as a white solid.

¹H NMR (400 MHz; $CDCl_3$) δ: 7.96 (1H, d, *J* = 8.3 Hz), 7.72 (1H, dd, *J* = 8.5, 1.0 Hz), 7.46 (1H, t, *J* = 8.2 Hz), 6.94 (1H, d, *J* = 7.8 Hz), 6.61 (1H, d, *J* = 8.4 Hz), 3.98 (3H, s), 3.96 (3H, s).

¹³C{¹H} NMR (100 MHz; $CDCl_3$) δ: 158.4, 157.5, 138.0, 137.0, 128.1, 125.6, 118.7, 107.8, 107.3, 88.8, 56.9, 56.7.

FTIR ν_{max} (ATR, solid)/cm^{-1} 3001, 2935, 2836, 1583, 1567, 1454, 1396, 1372, 1310, 1264, 1232.

HRMS (APCI+) Calcd for $C_{12}H_{12}IO_2$ [M+ H]$^+$: 314.9877, found: 314.9864.

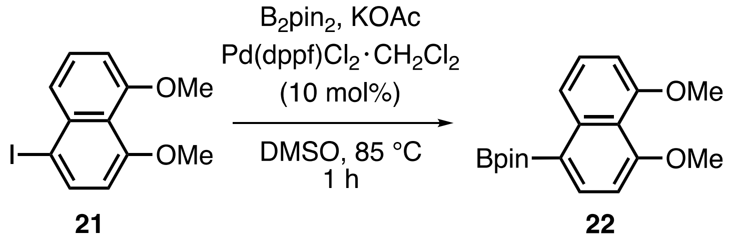

DMSO (5 mL) was degassed with N_2 gas for 5 min in a 25 mL-round bottomed flask prior to the reaction. Iodonaphthalene **21** (500 mg, 1.59 mmol), B_2pin_2 (806 mg, 3.18 mmol), $Pd(dppf)Cl_2{\cdot}CH_2Cl_2$ (130 mg, 0.159 mmol) and KOAc (625 mg, 6.37 mmol) were added sequentially. The flask was sealed with a glass stopper, and the reaction mixture was stirred at 85 °C for 1 h. Afterwards, the mixture was cooled down to ambient temperature and diluted with EtOAc. The organic phase was washed once with brine and once with water. The organic phase was then dried over Na_2SO_4, filtered and concentrated under reduced pressure. Purification by column chromatography (EtOAc:hexanes = 1:9) afforded the desired product **22** along with some unreacted B_2pin_2. By taking advantage of the lower solubility of compound **22** in hexanes than B_2pin_2, pure product **22** could be obtained by careful washing of the mixture by cold hexane (311 mg, 62%).

^{1}H NMR (400 MHz; $CDCl_3$) δ: 8.41 (1H, dd, *J* = 8.5, 0.9 Hz), 8.01 (1H, d, *J* = 7.9 Hz), 7.42 (1H, t, *J* = 8.2 Hz), 6.88 (1H, d, *J* = 7.4 Hz), 6.84 (1H, d, *J* = 8.0 Hz), 3.99 (3H, s), 3.96 (3H, s), 1.40 (12H, s).

^{13}C{^{1}H} NMR (100 MHz; $CDCl_3$) δ: 160.1, 157.5, 141.3, 137.4, 127.0, 121.5, 117.6, 106.5, 105.3, 83.6, 56.7, 56.3, 25.1.

FTIR ν_{max} (ATR, solid)/cm^{-1} 2985, 2930, 2832, 1580, 1513, 1466, 1319, 1269.

HRMS (APCI+) Calcd for $C_{18}H_{24}BO_4$ $[M+H]^+$: 315.1763, found: 315.1764.

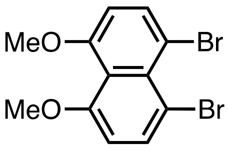

14

Compound **14** was adapted from a procedure reported in the literature.[6] In a 50-mL round-bottom flask, 1,8-dimethoxynaphthalene (**20**) (1.00 g, 5.31 mmol) was dissolved in 17 mL of anhydrous CH_2Cl_2. To the resulting yellow solution, *N*-bromosuccinimide (NBS) (945 mg, 5.31 mmol) was slowly added at 23 ºC. The dark-gray reaction mixture was stirred at 23 ºC for 40 min. CH_2Cl_2 was removed under reduced pressure. The remaining solid was dissolved in 17 mL of anhydrous DMF. To this solution, a second portion of NBS (993 mg, 5.58 mmol) was added slowly at 23 ºC. The resulting reddish-gray reaction mixture was stirred at 23 ºC for additional 40 min, and then the mixture was quenched with saturated aqueous $Na_2S_2O_3$ solution (20 mL). The aqueous phase was extracted with EtOAc (3 × 20 mL). The combined organic phase was dried over anhydrous Na_2SO_4, filtered and concentrated under reduced pressure. Purification by flash column chromatography (SiO_2; EtOAc:hexanes = 1:19 → 1:9 → 1:5→ 1:2→ 1:1) gave pure **14** (1.27 gram g, 69% yield) as goldish yellow solid.

^{1}H NMR (400 MHz; $CDCl_3$) δ: 7.82 (2H, d, *J* = 8.5 Hz), 6.72 (2H, d, *J* = 8.5 Hz), 3.93 (6H, s).
^{13}C{^{1}H} NMR (100 MHz; $CDCl_3$) δ: 157.8, 135.9, 131.6, 121.7, 110.3, 107.8, 56.9.
FTIR ν_{max} (ATR, solid)/cm^{-1} 3010, 2965, 2916, 2837, 1583, 1508, 1462, 1448, 1369, 1351, 1293, 1231.
HRMS (APCI+) calculated: $C_{12}H_{11}{}^{79}Br_2O_2$ [M+H]$^+$: 344.9121, found: 344.9122.

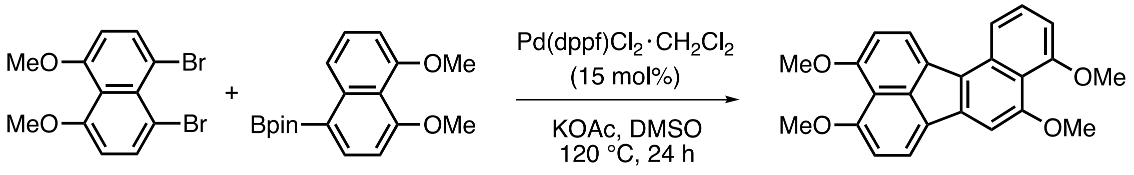

14 **22** **18**

DMSO (2.0 mL) was degassed by bubbling N_2 gas for 5 min in a 25 mL-round bottomed flask prior to the reaction. Dibromonaphthalene **14** (50.2 mg, 0.14 mmol), boronic ester **22** (50.0 mg, 0.16 mmol), Pd(dppf)Cl_2·CH_2Cl_2 (17.7 mg, 0.022 mmol)

and KOAc (57 mg, 0.58 mmol) were added sequentially. The flask was sealed with a glass stopper, and the reaction mixture was stirred at 120 °C for 24 h. Afterwards, the mixture was cooled down to ambient temperature and washed once with brine and once with water. The combined aqueous phase was extracted once with EtOAc. The combined organic phase was dried over Na_2SO_4, filtered and concentrated under reduced pressure. Purification was achieved by two sequential column chromatography separations: 1) 30% hexanes in CH_2Cl_2; 2) EtOAc:hexanes = 1:2. Pure benzo[*j*]fluoranthene product **18** (28.0 mg, 52% yield) was obtained as a yellow solid. The spectral data of compound **18** match the reported data in the literature.[7]

Note: When EtOAc was used as a component of the mobile phase in column chromatography, significant diffusion (trailing) of the product was observed. Therefore, using CH_2Cl_2 as the polar component of the mobile phase appears to be better.

^{1}H NMR (400 MHz; $CDCl_3$) δ: 8.25 (1H, d, *J* = 8.0 Hz), 8.24 (1H, dd, *J* = 8.5, 0.8 Hz), 7.92 (1H, d, *J* = 7.8 Hz), 7.50 (1H, t, *J* = 8.1 Hz), 7.43 (1H, s), 6.95 (1H, d, *J* = 8.0 Hz), 6.94 (1H, d, *J* = 7.8 Hz), 6.86 (1H, d, *J* = 7.8 Hz), 4.11 (3H, s), 4.084 (3H, s), 4.082 (3H, s), 4.02 (3H, s).

^{13}C{^{1}H} NMR (100 MHz; $CDCl_3$) δ: 158.9, 158.3, 157.2, 157.1, 137.7, 135.6, 133.7, 130.5, 129.3, 127.5, 126.3, 124.4, 122.2, 117.1, 117.0, 114.5, 107.0, 106.7, 105.8, 100.9, 57.1, 56.7, 56.62. 56.57.

FTIR ν_{max} (ATR, solid)/cm^{-1} 2992, 2960, 2933, 2833, 1584, 1460, 1424, 1402, 1249

HRMS (APCI+) Calcd for $C_{24}H_{21}O_4$ $[M+H]^+$ 373.1435, found: 373.1432.

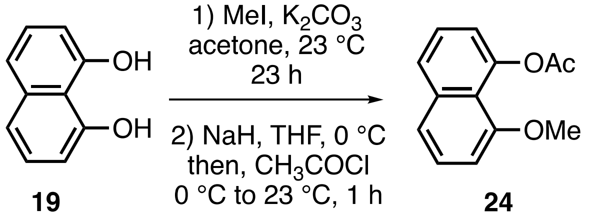

19 **24**

1,8-Dihydroxynaphthalene (1,8-DHN, **19**) (1.00 g, 6.24 mmol) was dissolved in 50 mL of acetone in a 100-mL round-bottomed flask at 23 °C under air. After the sequential

addition of K_2CO_3 (1.035 g, 7.49 mmol) and CH_3I (583 μL, 9.37 mmol), the resulting heterogeneous mixture was stirred at 23 °C for 23 h. TLC analysis indicated the full consumption of 1,8-DHN. The reaction mixture was quenched with saturated NH_4Cl solution (50 mL) and H_2O (50 mL). The aqueous phase was extracted with EtOAc (3 × 50 mL). The combined organic phase was washed once with brine (70 mL), dried over anhydrous Na_2SO_4, filtered and concentrated under reduced pressure to give a brown solid (1.052 g). This material was used directly in the next step without further purification.

8-Methoxy-1-naphthol (1.052 g, obtained via the above procedure) was dissolved in 30 mL of anhydrous THF at rt under nitrogen. The clear solution was cooled to 0 °C and stirred for 10 min. NaH (290 mg, 7.25 mmol, 60% dispersion in mineral oil) was added carefully in portions to the reaction mixture, and vigorous gas evolution was observed. After 10 min, acetyl chloride (CH_3COCl) (647 μL, 9.06 mmol) was slowly added. After an additional stirring of 10 min at 0 °C, the ice bath was removed, and the reaction mixture was stirred at 23 °C for 1 h. TLC analysis indicated the full consumption of 8-methoxy-1-naphthol. The reaction mixture was quenched with 20 mL of H_2O, and the aqueous phase was extracted with EtOAc (3 × 50 mL). The combined organic phase was dried over Na_2SO_4, filtered and concentrated under reduced pressure. Purification by column chromatography (EtOAc:hexanes = 1:9 → 1:4) afforded pure **24** (1.183 g, 88% over two steps) as a white solid. The ^{1}H-NMR spectral data match the reported data in the literature.[8]

Mp = 82-83 °C

***R*f** = 0.28 (EtOAc:hexanes = 1:9)

^{1}H NMR (400 MHz; $CDCl_3$) δ: 7.70 (1H, dd, *J* = 8.3, 1.1 Hz), 7.47-7.41 (2H, m), 7.38 (1H, t, *J* = 7.8 Hz), 7.08 (1H, dd, *J* = 7.4, 1.2 Hz), 6.85 (1H, d, *J* = 7.7 Hz), 3.93 (3H, s), 2.38 (3H, s).

^{13}C{^{1}H} NMR (100 MHz; $CDCl_3$) δ: 170.4, 155.3, 146.7, 137.1, 126.53, 126.52, 126.2, 121.2, 119.4, 119.3, 106.3, 56.2, 21.1.

FTIR ν_{max} (ATR, solid)/cm^{-1} 2968, 2935, 2842, 1742, 1599, 1579, 1462, 1368, 1267, 1206.

HRMS (APCI+) Calcd for $C_{13}H_{13}O_3$ $[M+H]^+$ 217.0860, found: 217.0866.

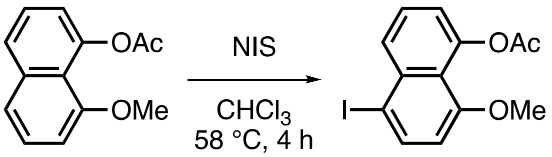

24 **25**

8-Methoxy-1-acetoxynaphthalene (**24**) (200 mg, 0.93 mmol) was dissolved in 2 mL of chloroform ($CHCl_3$) in a 25 mL round-bottomed flask at room temperature. After the addition of *N*-iodosuccinimide (916 mg, 4.07 mmol) and additional $CHCl_3$ (5 mL), the resulting mixture was stirred at 58 °C for 4 h with air-condenser. Afterwards, the reaction mixture was cooled to ambient temperature and quenched with saturated aqueous $Na_2S_2O_3$ solution (5 mL). The aqueous phase was extracted with EtOAc (2 × 10 mL). The combined organic phase was washed once with brine (10 mL), dried over anhydrous Na_2SO_4, filtered and concentrated under reduced pressure. Purification by column chromatography (EtOAc:hexanes = 1:9) afforded pure **25** (279 mg, 88%) as a white solid.

Mp = 135.1-136.2 °C

$\boldsymbol{R_f}$ = 0.40 (EtOAc:hexanes = 1:5)

^{1}H NMR (400 MHz; $CDCl_3$) δ: 8.02 (1H, dd, *J* = 8.6, 1.2 Hz), 7.98 (1H, d, *J* = 8.3 Hz), 7.53 (1H, dd, *J* = 8.6, 7.5 Hz), 7.15 (1H, dd, *J* = 7.5, 1.2 Hz), 6.62 (1H, d, *J* = 8.3 Hz), 3.92 (3H, s), 2.37 (3H, s).

^{13}C{^{1}H} NMR (100 MHz; $CDCl_3$) δ: 170.3, 156.3, 146.7, 137.9, 136.7, 131.2, 127.7, 120.4, 120.2, 107.8, 88.8, 56.4, 21.1.

FTIR ν_{max} (ATR, solid)/cm^{-1} 2963, 2919, 2850, 1744, 1587, 1566, 1502, 1455, 1362, 1311, 1254, 1207.

HRMS (APCI+) Calcd for $C_{13}H_{12}IO_3$ $[M+H]^+$: 342.9826, found: 342.9837.

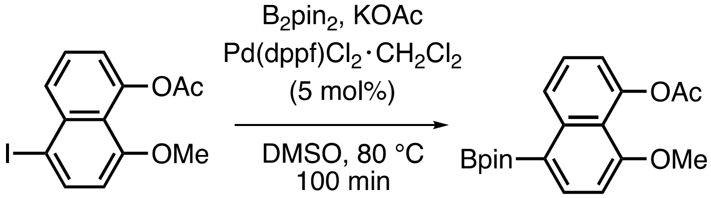

25 **26**

DMSO (5 mL) was degassed by bubbling N_2 gas for 5 min in a 25 mL, oven-dried round bottomed flask prior to the reaction. Iodonaphthalene **25** (400 mg, 1.17 mmol), B_2pin_2 (326 mg, 1.29 mmol), Pd(dppf)Cl_2·CH_2Cl_2 (48 mg, 0.058 mmol) and KOAc (344 mg, 3.51 mmol) were added sequentially to the solution. The flask was then sealed with a glass stopper, and the reaction mixture was stirred at 80 ºC for 100 min. TLC analysis at the end of this time indicated full consumption of the starting material. After cooling to room temperature, brine (ca. 10 mL) was added to the reaction mixture, and the aqueous phase was extracted with EtOAc (2 × 10 mL). The combined organic phase was dried over Na_2SO_4, filtered, and concentrated under vacuum. Purification by flash column chromatography (CH_2Cl_2:hexanes = 1:1 → 2:1 → only CH_2Cl_2) afforded boronic ester **26** (282 mg, 71% yield) as a white solid.

M.P. = 168.6-169.8 °C
***R*f** = 0.41 (EtOAc:hexanes = 1:5)
^{1}H NMR (400 MHz; $CDCl_3$) δ: 8.73 (1H, dd, *J* = 8.7, 1.1 Hz), 8.04 (1H, d, *J* = 8.0 Hz), 7.50 (1H, dd, *J* = 8.7, 7.4 Hz), 7.07 (1H, dd, *J* = 7.5, 1.3 Hz), 6.84 (1H, d, *J* = 8.0 Hz), 3.94 (3H, s), 2.37 (3H, s), 1.40 (12H, s).
^{13}C{^{1}H} NMR (100 MHz; $CDCl_3$) δ: 170.4, 158.1, 146.7, 140.8, 137.4, 127.2, 126.6, 119.3, 119.2, 105.5, 83.7, 56.2, 25.1, 21.2.
FTIR ν_{max} (ATR, solid)/cm^{-1} 2975, 1756, 1587, 1511, 1459, 1360, 1332, 1255, 1218.
HRMS (APCI+) Calcd for $C_{19}H_{24}BO_5$ [M+H]$^+$: 343.1712, found: 343.1737.

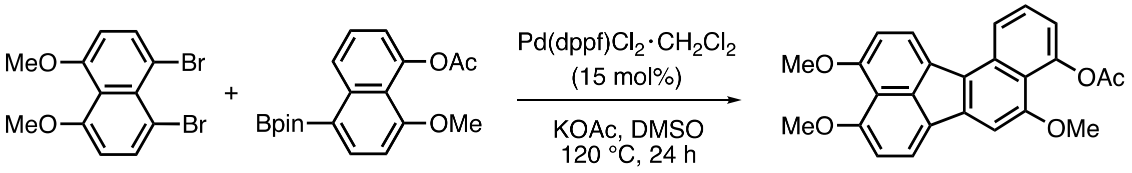

14 **26** **27**

DMSO (2.0 mL) was degassed by bubbling N_2 gas for 5 min in a 25 mL-round bottomed flask prior to the reaction. Dibromonaphthalene **14** (50.0 mg, 0.14 mmol), boronic ester **26** (54.4 mg, 0.16 mmol), Pd(dppf)Cl_2·CH_2Cl_2 (17.7 mg, 0.022 mmol)

and KOAc (57 mg, 0.58 mmol) were added sequentially. The flask was sealed with a glass stopper, and the reaction mixture was stirred at 120 °C for 24 h. The progress of the reaction was monitored by TLC. After cooling to room temperature, brine (ca. 10 mL) was added to the reaction mixture, and the aqueous phase was extracted with EtOAc (2 × 10 mL). The combined organic phase was dried over Na_2SO_4, filtered, and concentrated under vacuum. Purification by column chromatography (SiO_2; EtOAc:hexanes = 1:3 → 1:2 → 1:1→ 2:1 → only EtOAc) gave pure **27** (19.8 mg, 34% yield) as light yellow solid.

***R*$_f$** = 0.55 (EtOAc:hexanes = 1:1)

^{1}H NMR (400 MHz; CDCl$_3$) δ: 8.53 (1H, d, *J* = 8.5 Hz), 8.26 (1H, d, *J* = 8.0 Hz), 7.91 (1H, d, *J* = 7.8 Hz), 7.55 (1H, dd, *J* = 8.3, 7.5 Hz), 7.40 (1H, s), 7.04 (1H, dd, *J* = 7.4, 1.0 Hz), 6.97 (1H, d, *J* = 7.4 Hz), 6.95 (1H, d, *J* = 7.6 Hz), 4.09 (6H, s), 4.07 (3H, s), 2.41 (3H, s).

^{13}C NMR (100 MHz; CDCl$_3$) δ: 170.4, 159.1, 157.4, 155.0, 147.7, 137.6, 135.4, 133.3, 130.0, 129.1, 127.0, 126.4, 124.5, 122.6, 122.4, 118.6, 118.5, 114.5, 107.0, 106.7, 100.6, 56.64, 56.59, 56.57, 21.2

FTIR ν_{max} (ATR, solid)/cm^{-1} 2968, 2836, 1758, 1589, 1459, 1427, 1404, 1363.

HRMS (+APCI) Calcd for $C_{25}H_{21}O_5$ $[M+H]^+$: 401.1384, found: 401.1390.

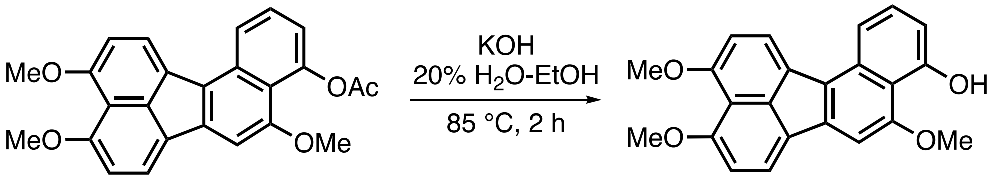

27 **23**

KOH (6.5 mg, 0.11 mmol) was added to a solution of benzo[*j*]fluoranthene **27** (15 mg, 0.038 mmol) in 20% H_2O-EtOH mixture (1.6 mL of EtOH and 0.4 mL of H_2O) at room temperature, and the mixture was heated at 85 °C for 2 h. It was then cooled down to ambient temperature and quenched with 1.0 M aqueous HCl solution, followed by the addition of 4 mL of saturated aqueous NH_4Cl solution. The aqueous phase was extracted with CH_2Cl_2 (3 × 4 mL). The combined organic phase was dried over

Na_2SO_4, filtered and concentrated under vacuum. Purification by flash column chromatography (EtOAc:hexanes = 1:1) gave hydrolysis product **23** as a dark yellow solid (12.2 mg, 91% yield).

R_f = 0.61 (EtOAc:hexanes = 1:1)

^{1}H NMR (400 MHz; $CDCl_3$) δ: 9.64 (1H, s), 8.28 (1H, d, *J* = 8.0 Hz), 8.11 (1H, d, *J* = 8.7 Hz), 7.91 (1H, d, *J* = 7.7 Hz), 7.48 (1H, t, *J* = 8.0 Hz), 7.35 (1H, s), 6.97 (1H, d, *J* = 8.3 Hz), 6.95 (1H, d, *J* = 8.2 Hz), 6.87 (1H, d, *J* = 7.6 Hz), 4.21 (3H, s), 4.09 (6H, s).

FTIR ν_{max} (ATR, solid)/cm^{-1} 3360, 2922, 2851, 1587, 1454, 1422, 1397, 1368.

HRMS (+APCI) Calcd for $C_{23}H_{19}O_4$ $[M+H]^+$: 359.1278, found: 359.1285.

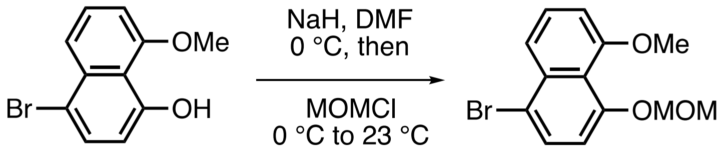

29 **30**

An oven-dried 100-mL round-bottomed flask was cooled under vacuum and refilled with N_2 (×3). Naphthalene derivative **29** (2.38 g, 9.40 mmol) was added and dissolved in 20 mL of anhydrous DMF under N_2 at 23 °C. The resulting clear pale yellow solution was cooled down to 0 °C in an ice bath for 10 min. Sodium hydride (NaH, 451 mg, 11.3 mmol, 60% dispersion in mineral oil) was slowly added resulted in gas evolution. After 10 min of stirring at 0 °C, MOMCl (1.07 mL, 14.1 mmol) was added carefully. The ice bath was removed after 5 min, and the reaction mixture stirred at 23 °C. TLC indicated the full consumption of **29** after 90 min. The reaction mixture was quenched with saturated aqueous NH_4Cl solution (20 mL). The aqueous phase was extracted with EtOAc (3 × 20 mL). The combined organic phase was dried over anhydrous Na_2SO_4, filtered and concentrated under reduced pressure. Purification by column chromatography (SiO_2; EtOAc:hexanes = 1:9 → 1:5) gave pure **30** (2.68 g, 96% yield) as white solid.

Note: In another experiment, reaction product **30** was isolated in quantitative yield when the reaction was conducted starting from 1.5 g (5.93 mmol) of **29**. The spectral data of compound **30** match the reported data in the literature.[9]

TLC Images after aqueous work-up:

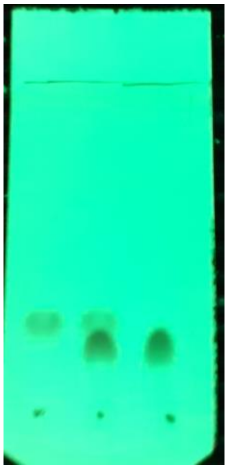

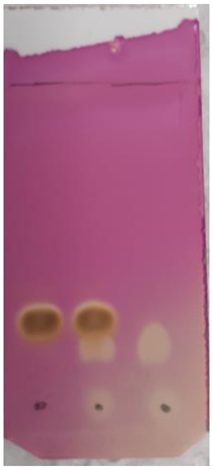

Left image: TLC under UV light (254 nm) **Right image:** TLC stained with $KMnO_4$ solution

Spots from left to right:

Left spot: Starting material (**29**);

Middle spot: Co-spot of **29** and reaction mixture;

Right spot: Reaction mixture.

Mobile phase: EtOAc:hexanes = 1:19 (developed twice)

R$_f$ = 0.50 (EtOAc:hexanes = 1:4)

TLC Visualization: UV active; stains with $KMnO_4$ solution to yellow upon heating.

^{1}H NMR (400 MHz; $CDCl_3$) δ: 7.85 (1H, d, J = 8.6 Hz), 7.67 (1H, d, J = 8.3 Hz), 7.48 (1H, t, J = 8.2 Hz), 6.94 (2H, t, J = 7.7 Hz), 5.25 (2 H, s), 3.97 (3H, s), 3.59 (3H, s).

^{13}C{^{1}H} NMR (100 MHz; $CDCl_3$) δ: 157.1, 154.1, 135.1, 130.5, 127.8, 120.4, 120.0, 116.0, 113.8, 107.3, 97.0, 56.7, 56.6.

FTIR ν_{max} (ATR, film)/cm^{-1} 1613, 1588, 1575, 1460, 1397, 1375, 1266, 1233, 1150, 1095, 994, 948, 815, 751.

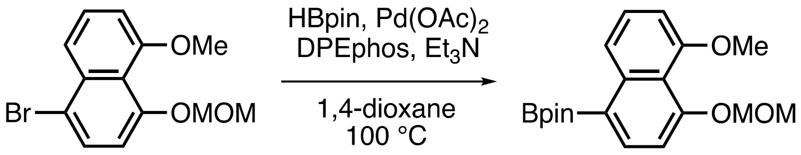

30 **31**

To a pale yellow solution of **30** (500 mg, 1.68 mmol) in 3 mL of 1,4-dioxane, Et_3N (0.94 mL, 6.72 mmol), $Pd(OAc)_2$ (37.7 mg, 0.168 mmol) and DPEphos (183 mg, 0.34

mmol) were added sequentially. To the resulting brown solution HBpin (730 µL, 5.04 mmol) was added dropwise resulting in a color change to dark brown and gas evolution. The reaction mixture was then heated to 100 ºC and stirred for 2 h. The reaction was stopped and the mixture was cooled to 23 ºC. Purification by flash column chromatography (SiO_2; only hexanes) gave pure **31** (324 mg, 56%) as green oil.

TLC Images after aqueous work-up:

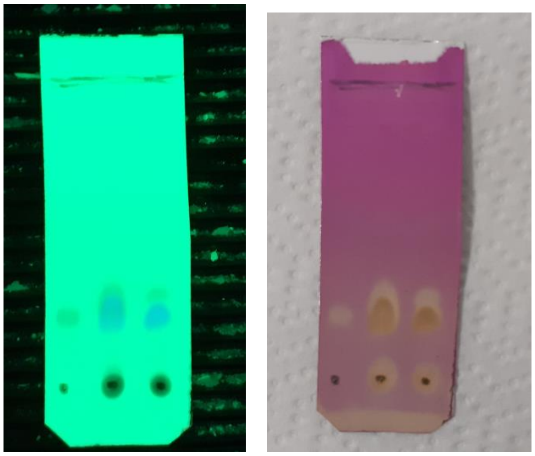

Left image: TLC under UV light (254 nm) **Right image:** TLC stained with $KMnO_4$ solution

Spots from left to right:
Left spot: starting material (**30**);
Middle spot: Co-spot of **30** and reaction mixture;
Right spot: Reaction mixture.
Mobile phase: EtOAc:hexanes = 1:19

$\boldsymbol{R_f}$ = 0.39 (EtOAc:hexanes = 1:9)
TLC Visualization: UV active; stains to dark yellow with $KMnO_4$ solution.
^{1}H NMR (400 MHz; $CDCl_3$) δ: 8.41 (1H, d, *J* = 8.5 Hz), 8.00 (1H, d, *J* = 7.8 Hz), 7.43 (1H, t, *J* = 8.0 Hz), 7.04 (1H, d, *J* = 7.8 Hz), 6.88 (1H, d, *J* = 7.7 Hz), 5.31 (2H, s), 3.96 (3H, s), 3.59 (3H, s), 1.40 (12H, s).
^{13}C{^{1}H} NMR (100 MHz; $CDCl_3$) δ: 157.1, 157.0, 141.4, 137.1, 126.8, 121.5, 118.4, 111.4, 106.5, 96.3, 83.6, 56.55, 56.54, 25.0
FTIR ν_{max} (ATR, film)/cm^{-1} 1613, 1578, 1515, 1464, 1326, 1326, 1262, 1142, 1099.

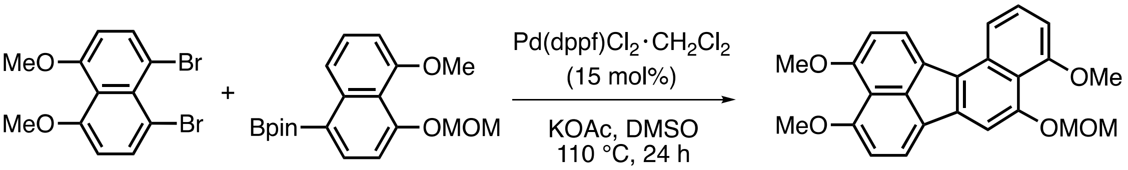

14 **31** **32**

To a green solution of boronic ester **31** (400 mg, 1.16 mmol) in DMSO (7 mL, purged with N_2 for 15 min) was added **14** (365 mg, 1.06 mmol). To this light brown suspension Pd(ddpf)Cl_2·CH_2Cl_2 (130 mg, 0.16 mmol) was added resulting in a color change to red. After the addition of KOAc (417 mg, 4.24 mmol), the flask was closed with a glass stopper and heated to 110 ºC. TLC indicated the full consumption of **14** after 24 h. After the reaction was stopped, the reaction mixture was cooled to 23 ºC and quenched with H_2O. The aqueous phase was extracted three times with EtOAc. The combined organic phase was dried over anhydrous Na_2SO_4, filtered and concentrated under reduced pressure. Purification by column chromatography (SiO_2; EtOAc:hexanes = 1:5 → 1:3 → 1:2 → 1:1 → only EtOAc) gave pure **32** (192 mg, 45% yield) as goldish yellow solid.

TLC Images:

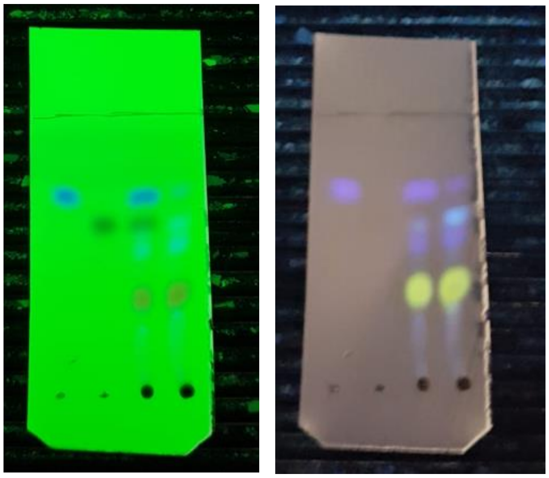

Left image: TLC under UV light (254 nm) **Right image:** TLC under UV light (366nm)

Spots from left to right:

1st spot: Naphthalene boronic ester **31**;

2nd spot: Dibromonaphthalene **14**

3rd spot: Co-spot of **31**, **14** and reaction mixture;

4th spot: Reaction mixture.

Mobile phase: EtOAc:hexanes = 1:2

M.P. = 215.2-216.4 ºC ($CHCl_3$).

$\boldsymbol{R_f}$ = 0.37 (EtOAc:hexanes = 1:2)

TLC Visualization: UV active; yellow under 366 nm.

^{1}H NMR (400 MHz; $CDCl_3$) δ: 8.29-8.25 (2H, m), 7.93 (1H, d, *J* = 7.8 Hz), 7.63 (1H, s), 7.49 (1H, t, *J* = 8.1 Hz), 6.95 (2H, t, *J* = 8.0 Hz), 6.86 (1H, d, *J* = 7.7 Hz), 5.36 (2H, s), 4.08 (6H, s), 4.01 (3H, s), 3.68 (3H, s).

^{13}C{^{1}H} NMR (100 MHz; $CDCl_3$) δ: 158.9, 157.9, 157.4, 153.7, 137.5, 135.6, 133.6, 130.2, 129.1, 128.1, 127.3, 124.8, 122.6, 118.0, 117.3, 114.4, 108.2, 106.9, 106.7, 105.7, 97.7, 56.7, 56.61, 56.56, 56.49.

FTIR ν_{max} (ATR, solid)/cm^{-1}1585, 1458, 1425, 1395, 1283, 1251, 1154, 1124, 1093, 1050.

HRMS (ESI+) Calcd for $C_{25}H_{22}O_5Na$ $[M+Na]^+$: 425.1360, found: 425.1362.

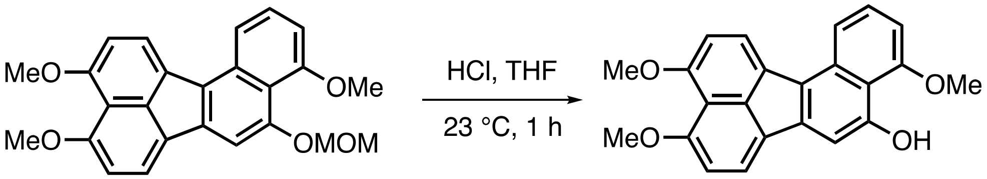

32 **28**

Fluoranthene derivative **32** (100 mg, 0.25 mmol) was dissolved in 10 mL of anhydrous THF with the aid of heating by a heat gun. To this yellowish-orange solution, concentrated HCl solution (0.83 mL, 10 mmol, ca. 12 M) was added dropwise at 23 ºC resulting in a color change to green and the formation of black insoluble particles. The reaction mixture was stirred at this temperature for 1 h. TLC indicated the full consumption of **32** after 1 h. The reaction mixture was then treated with H_2O (20 mL). The aqueous phase was extracted with EtOAc (3 × 20 mL). The combined organic phase was dried over anhydrous Na_2SO_4, filtered and concentrated under reduced pressure. Purification by column chromatography (SiO_2; EtOAc:hexanes = 1:6 → 1:4 → 1:1 → only EtOAc) gave pure **28** (72.6 mg, 82% yield) as yellowish orange solid.

TLC Images:

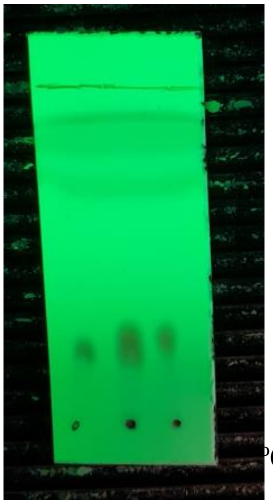

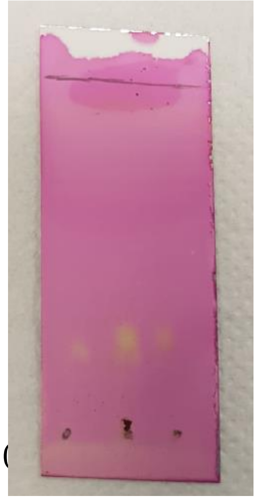

Left image: TLC under UV light (254 nm) **Right image:** TLC stained with $KMnO_4$ solution

Spots from left to right:

Left spot: Starting material (**32**);

Middle spot: Co-spot of **32** and reaction mixture;

Right spot: reaction mixture

R

TLC Visualization: UV active; yellow under 366 nm; stains to yellow with $KMnO_4$ solution.

^{1}H NMR (400 MHz; $CDCl_3$) δ: 9.64 (1H, s), 8.23 (1H, d, *J* = 8.6 Hz), 8.18 (1H, d, *J* = 7.9 Hz), 7.90 (1H, d, 7.7 Hz), 7.45 (1H, d, *J* = 7.9 Hz), 7.42 (1H, s), 6.95 (2H, d, *J* =7.9 Hz), 6.78 (1H, d, *J* = 7.7 Hz), 4.10 (3H, s), 4.08 (6H, s).

^{13}C{^{1}H} NMR (100 MHz; $CDCl_3$) δ: 158.9, 157.3, 156.9, 154.5, 139.2, 135.6, 132.8, 130.5, 129.2, 126.8, 124.8, 123.6, 122.6, 118.4, 114.5, 114.2, 106.9, 106.8, 104.6, 103.4, 56.62, 56.59, 56.3.

FTIR ν_{max} (ATR, solid)/cm^{-1} 3393, 1595, 1458, 1424, 1399, 1380, 1273, 1243, 1158, 1116, 1088, 811.

HRMS (ESI+) Calcd for $C_{23}H_{18}NaO_4$ $[M+Na]^+$: 381.1098, found: 381.1108.

^{1}H and ^{13}C{^{1}H} NMR spectra:

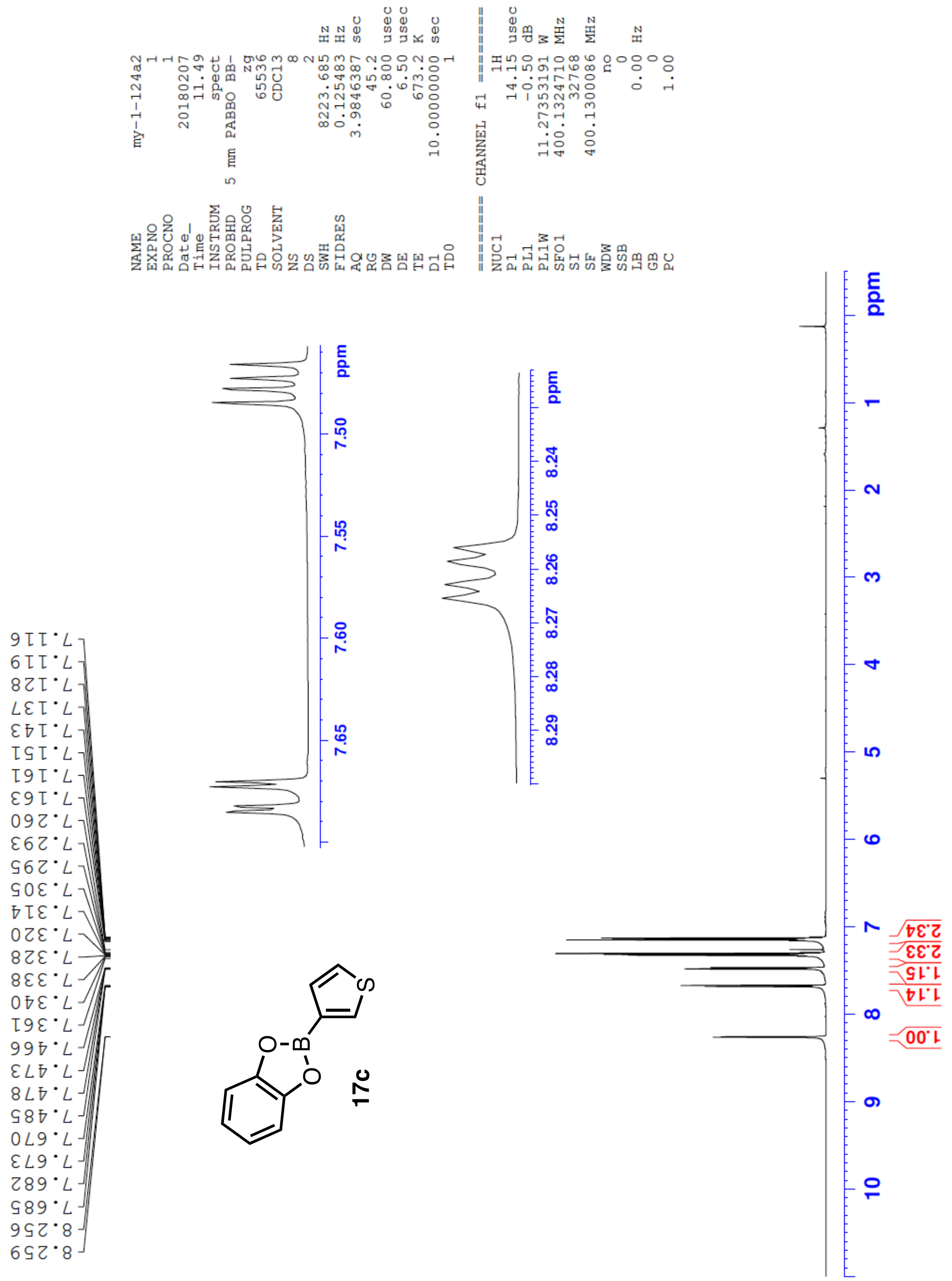

Figure S1. ^{1}H NMR spectrum of **17c** in $CDCl_3$.

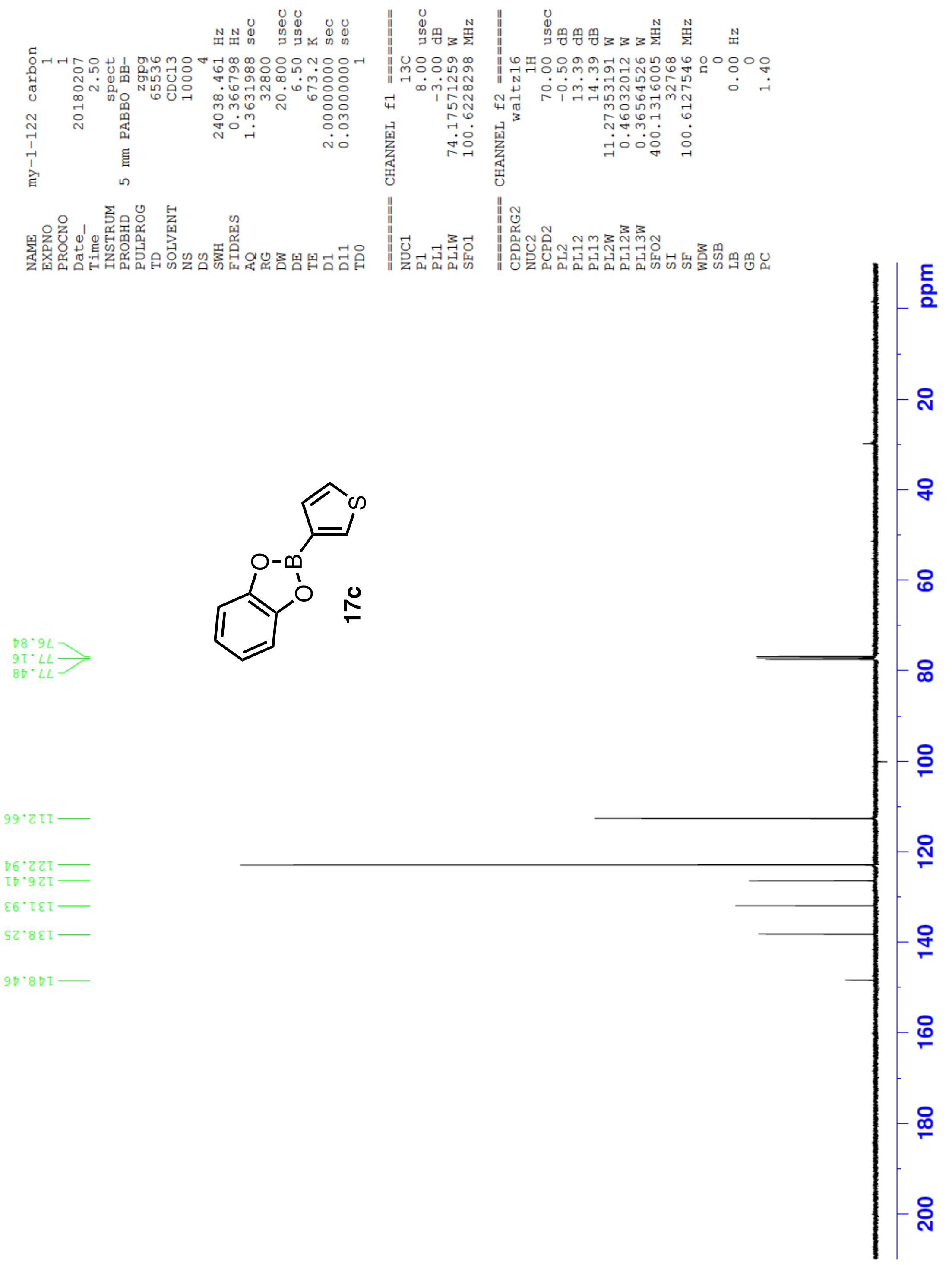

Figure S2. $^{13}C\{^1H\}$ NMR spectrum of **17c** in $CDCl_3$.

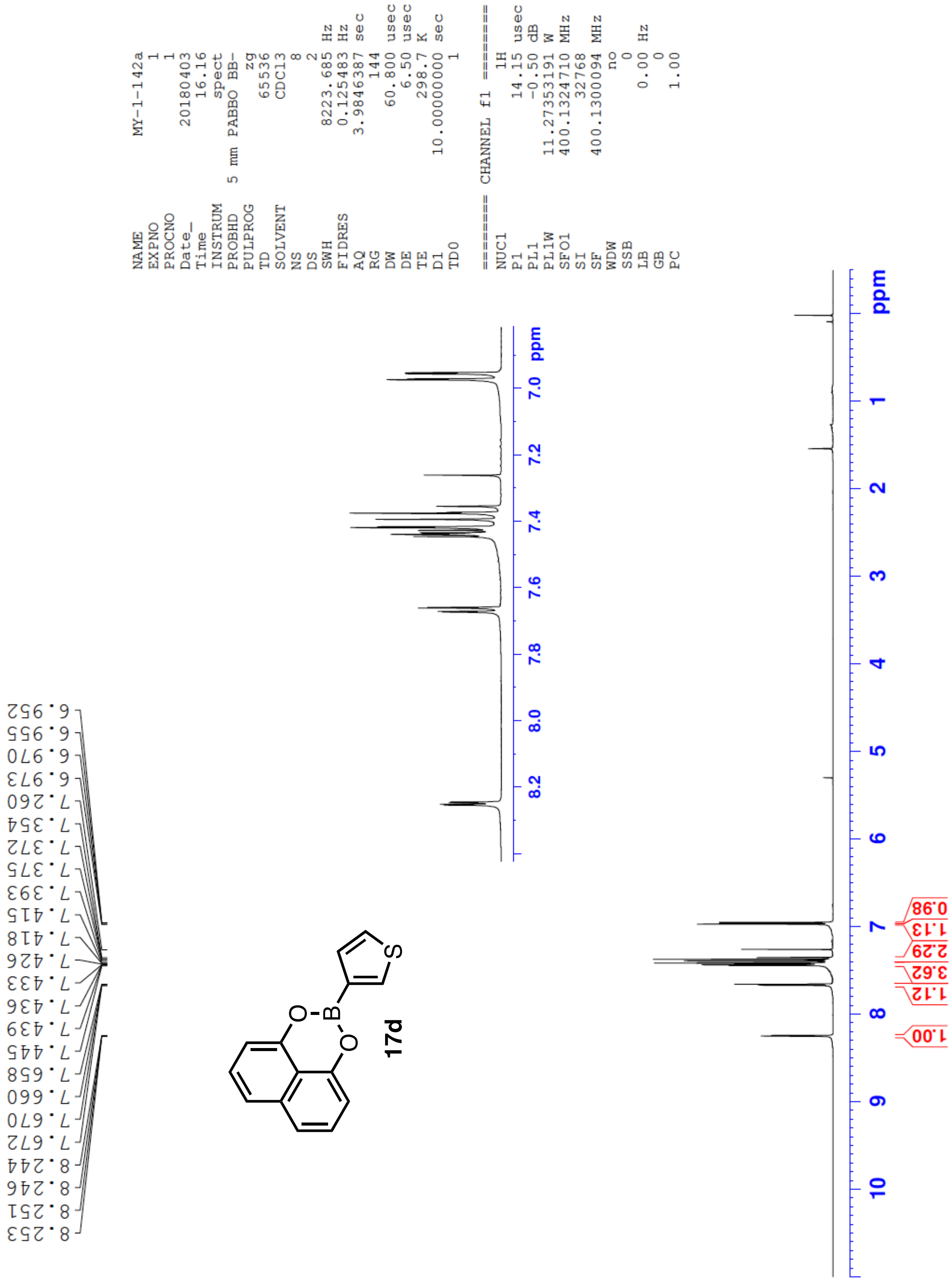

Figure S3. ^{1}H NMR spectrum of **17d** in $CDCl_3$.

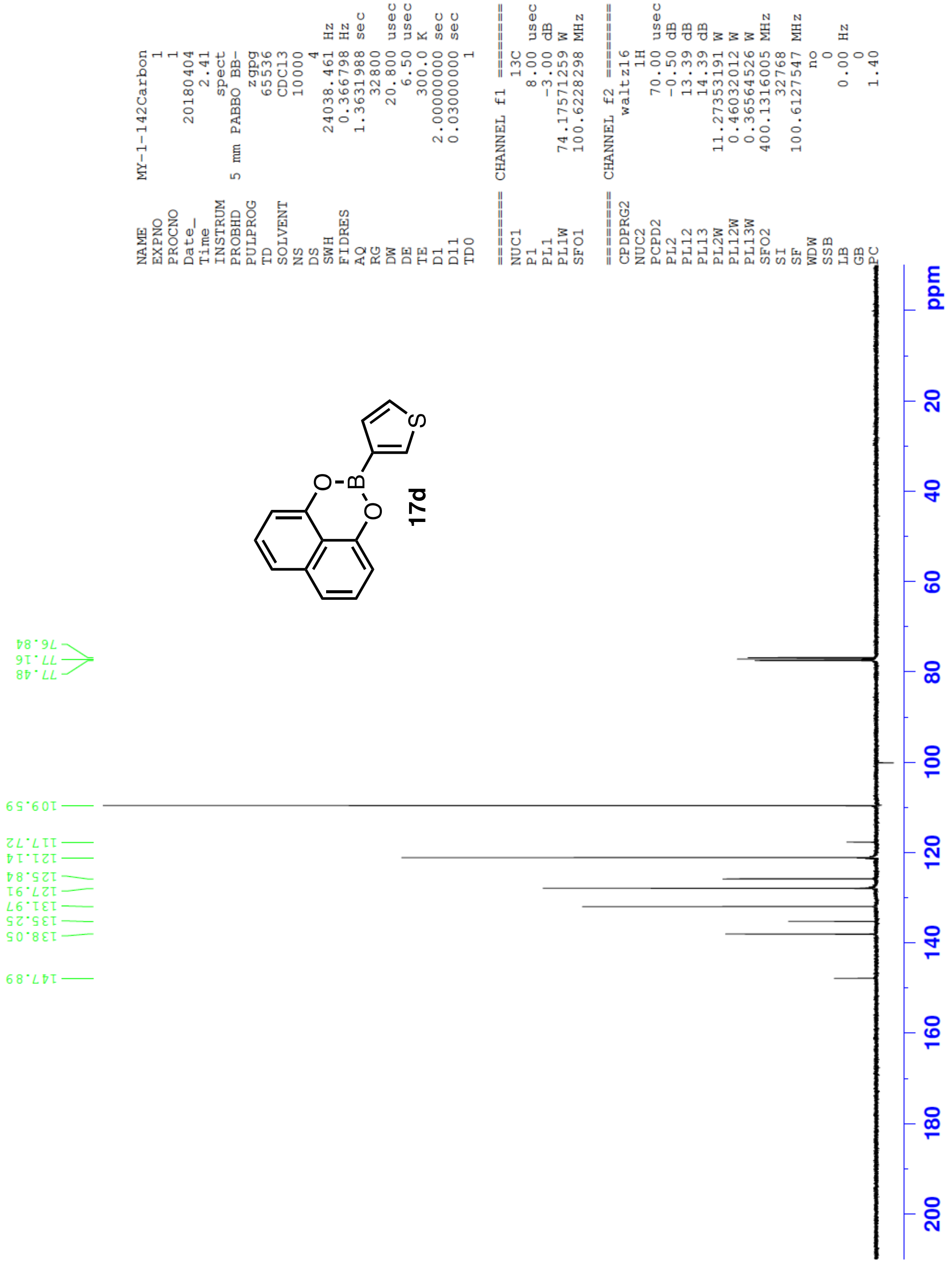

Figure S4. $^{13}C\{^{1}H\}$ NMR spectrum of **17d** in $CDCl_3$.

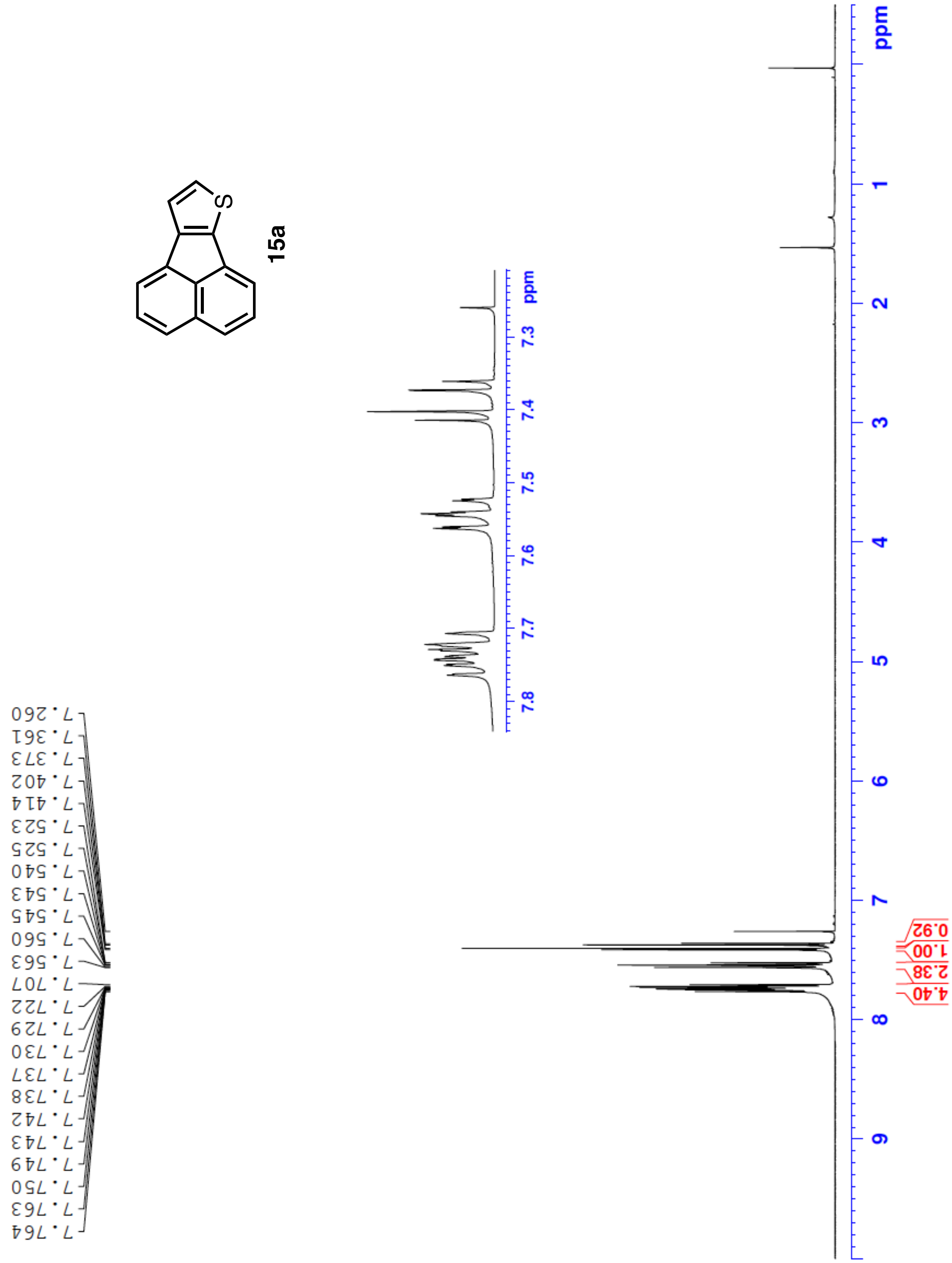

Figure S5. ^{1}H NMR spectrum of **15a** in $CDCl_3$.

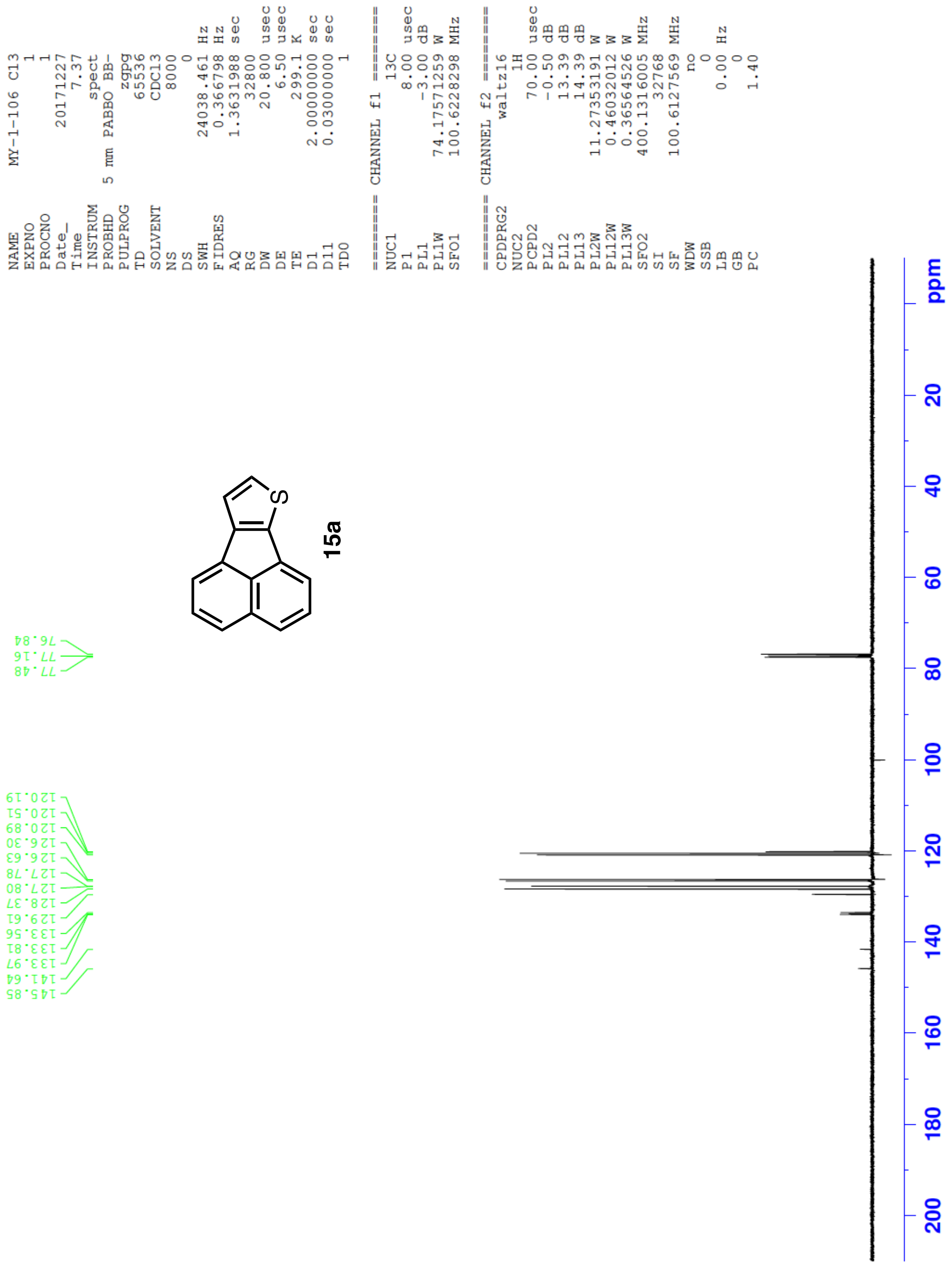

Figure S6. $^{13}C\{^{1}H\}$ NMR spectrum of **15a** in $CDCl_3$.

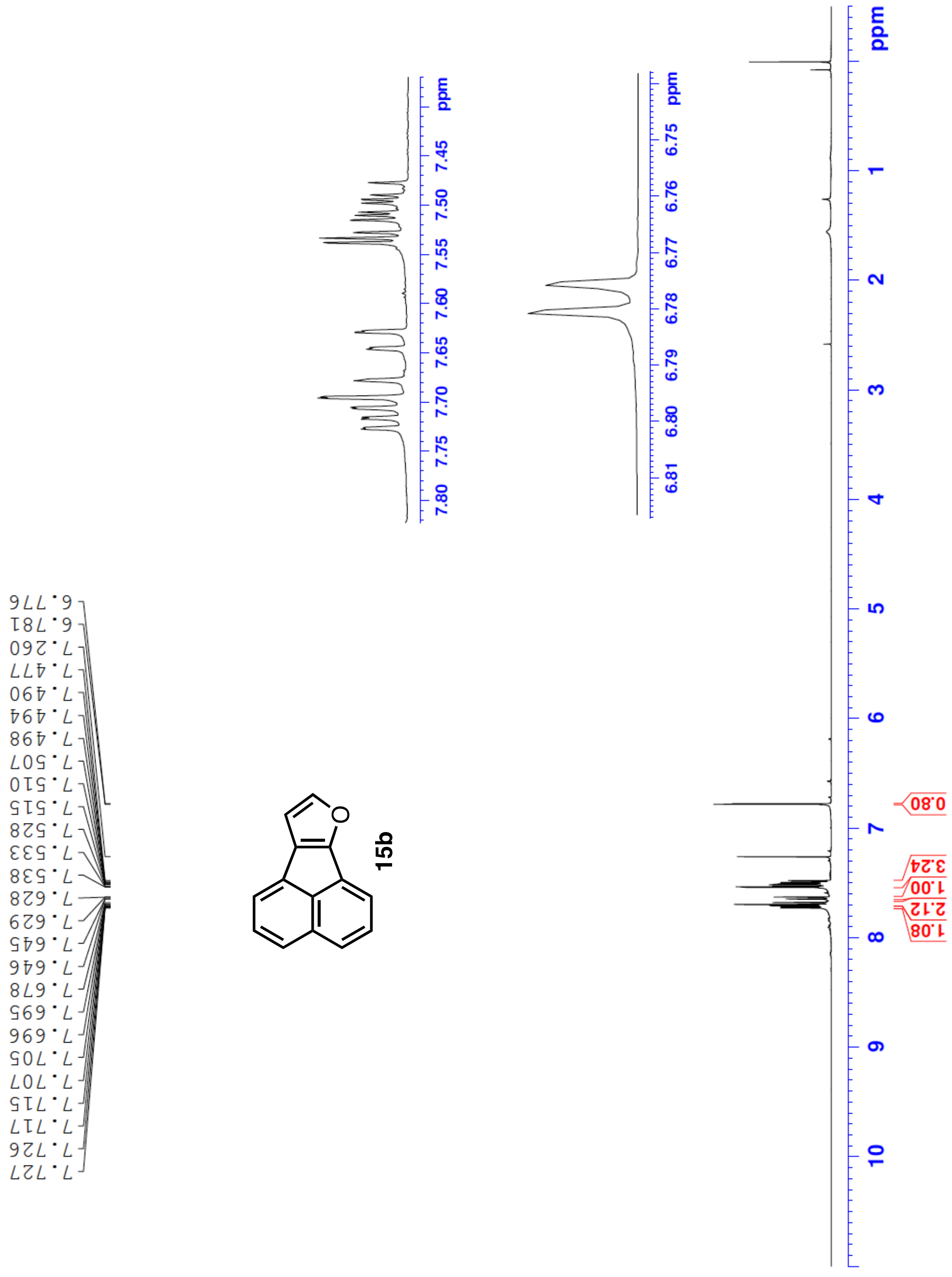

Figure S7. ^{1}H NMR spectrum of **15b** in $CDCl_3$.

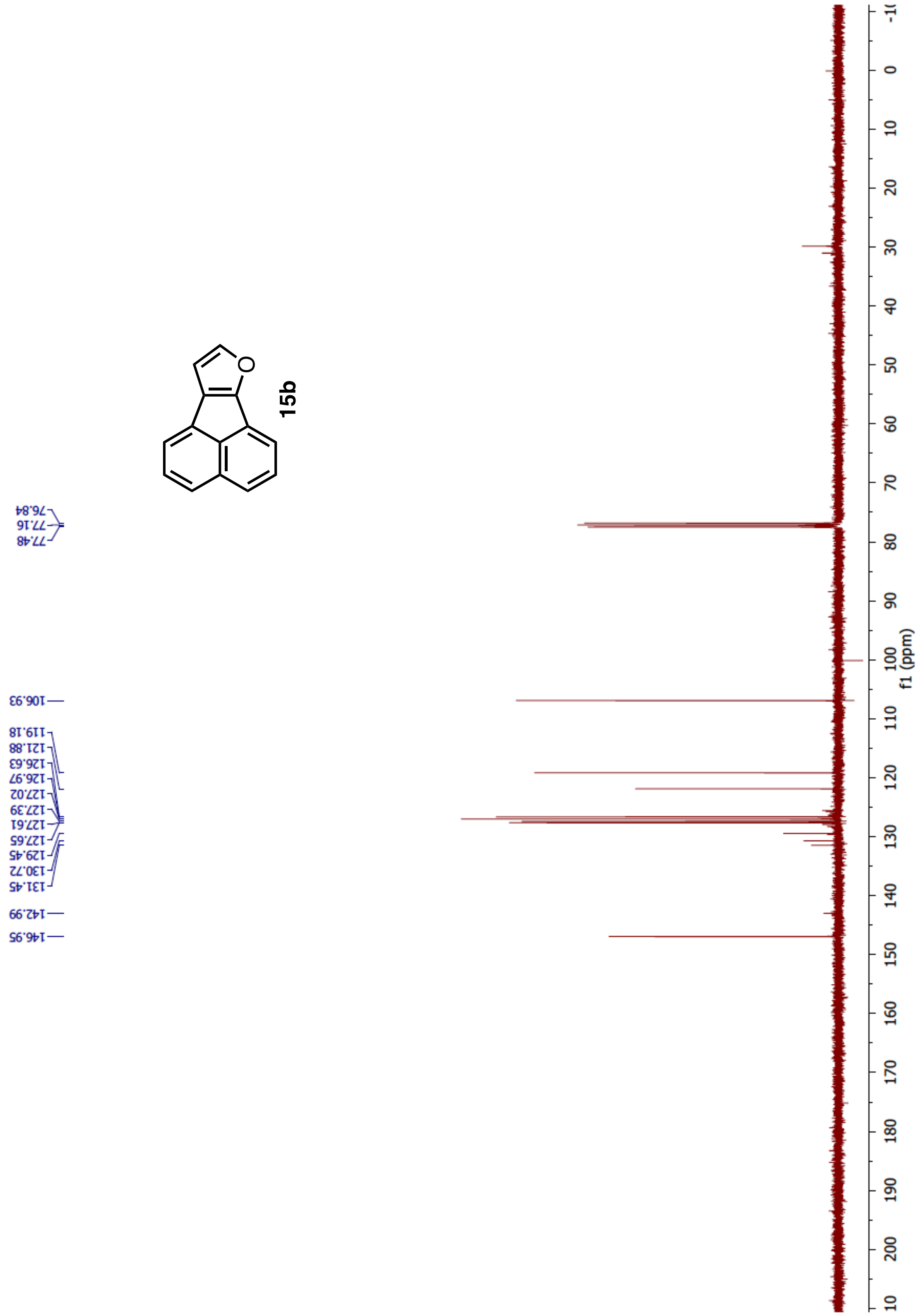

Figure S8. $^{13}C\{^{1}H\}$ NMR spectrum of **15b** in $CDCl_3$.

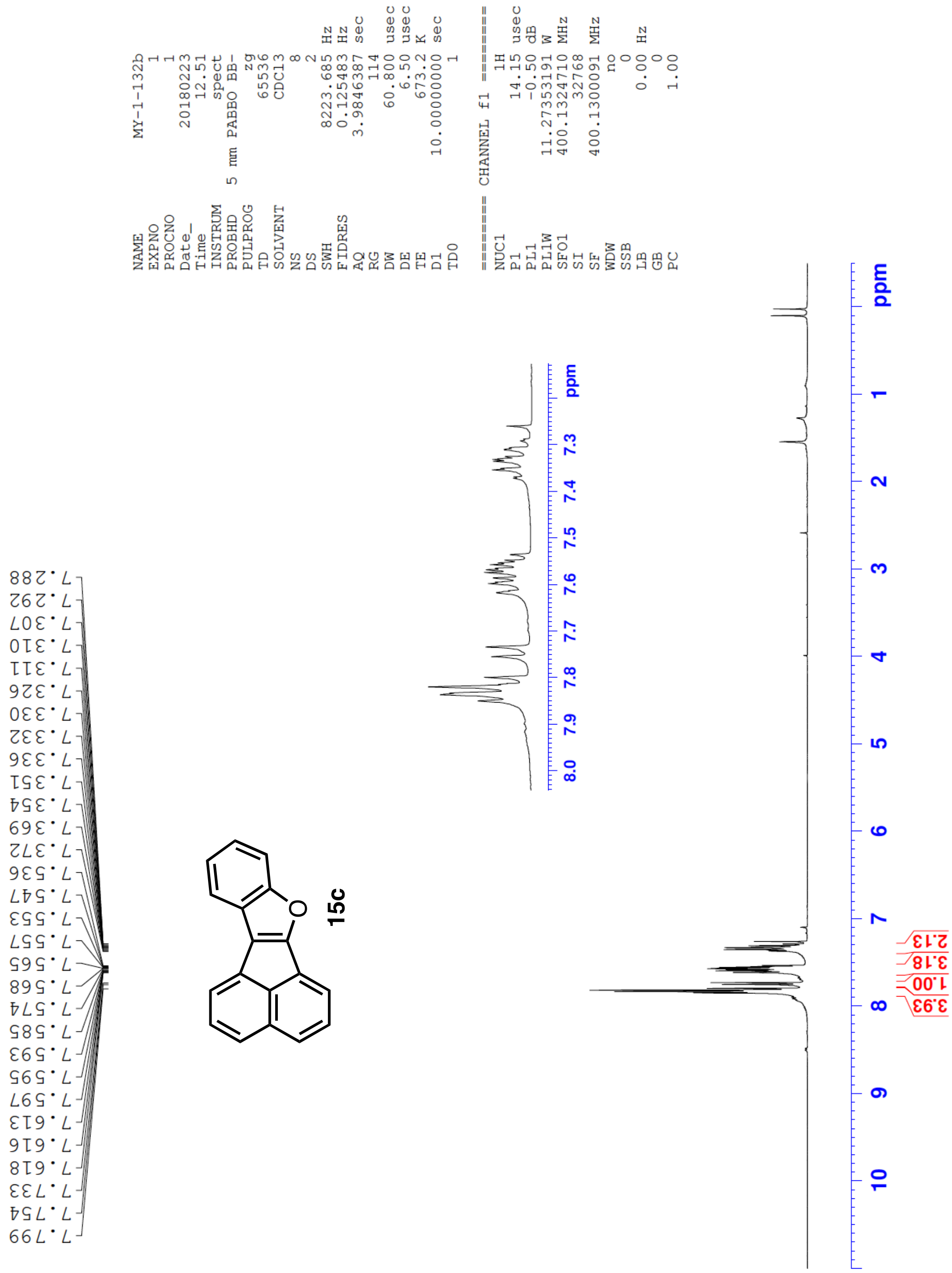

Figure S9. ^{1}H NMR spectrum of **15c** in $CDCl_3$.

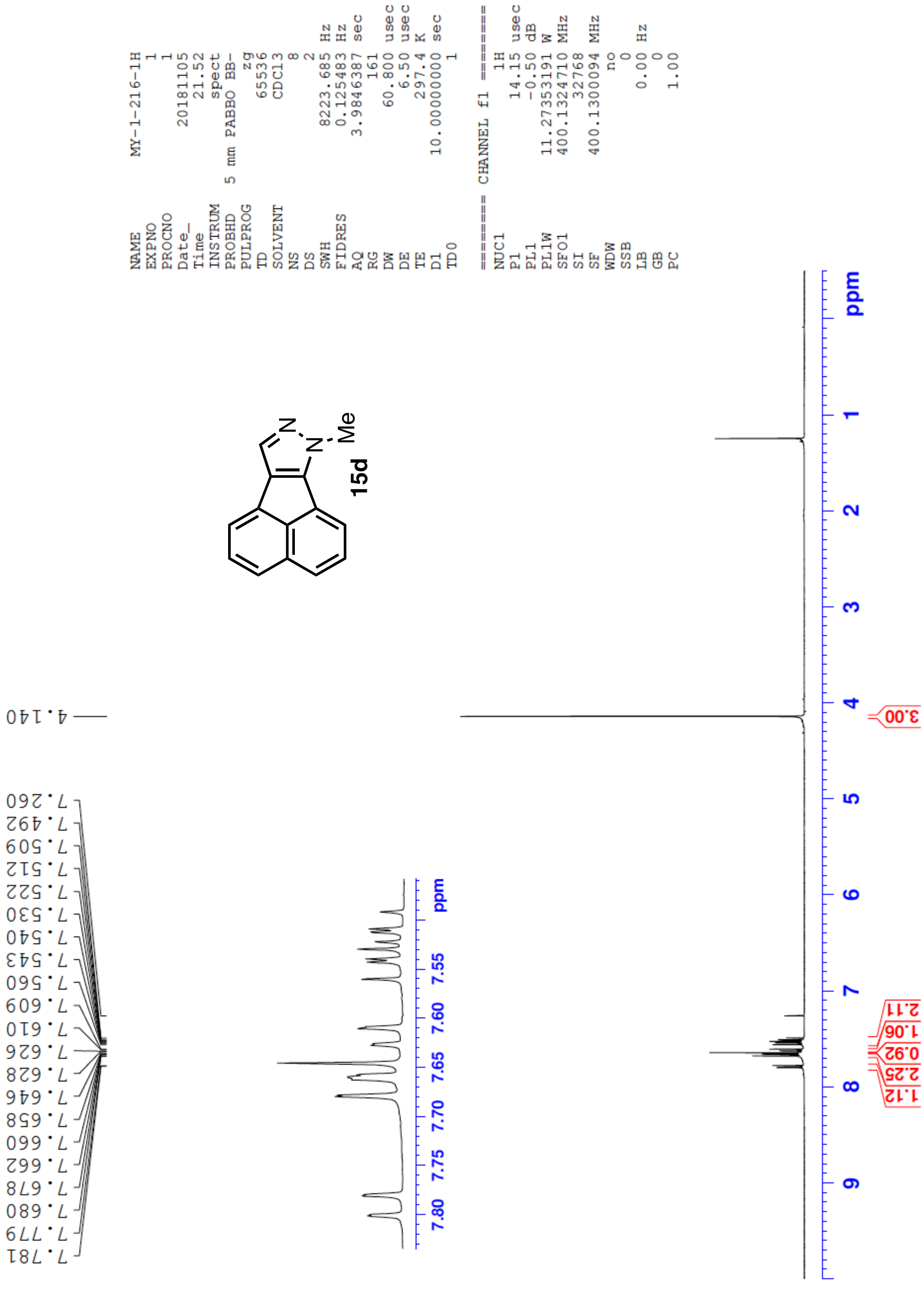

Figure S10. ^{1}H NMR spectrum of **15d** in $CDCl_3$.

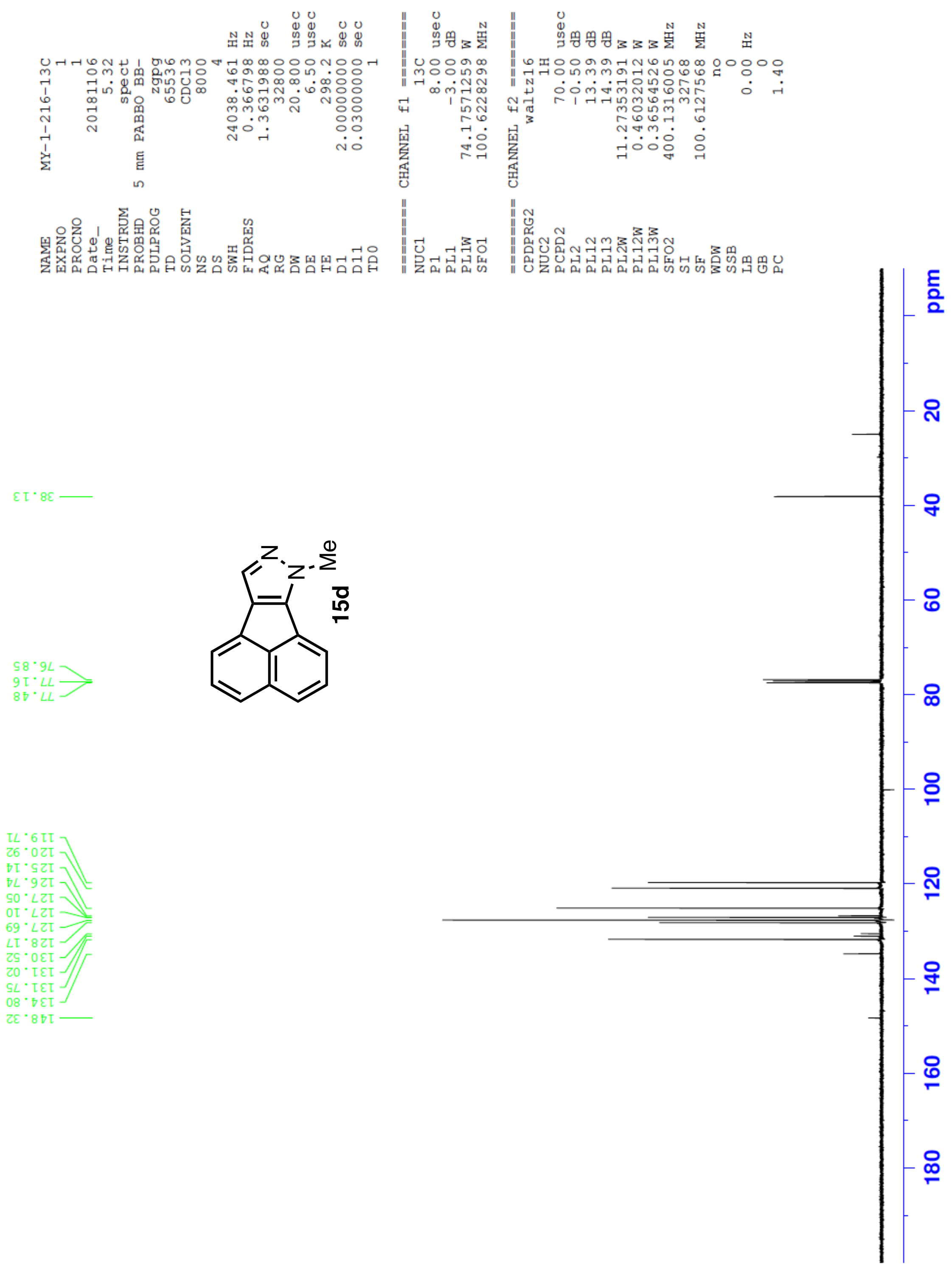

Figure S11. $^{13}C\{^1H\}$ NMR spectrum of **15d** in $CDCl_3$.

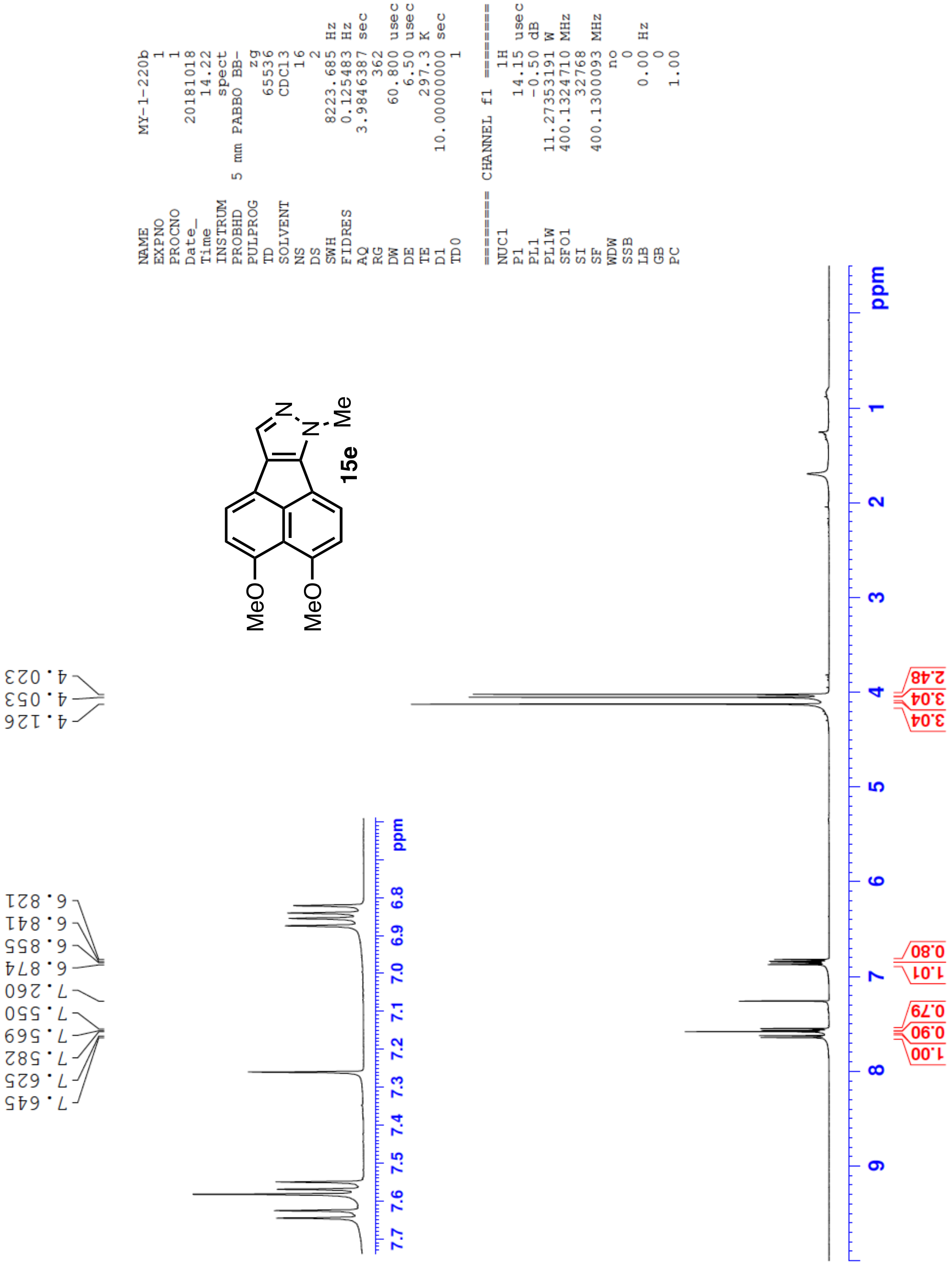

Figure S12. ^{1}H NMR spectrum of **15e** in $CDCl_3$.

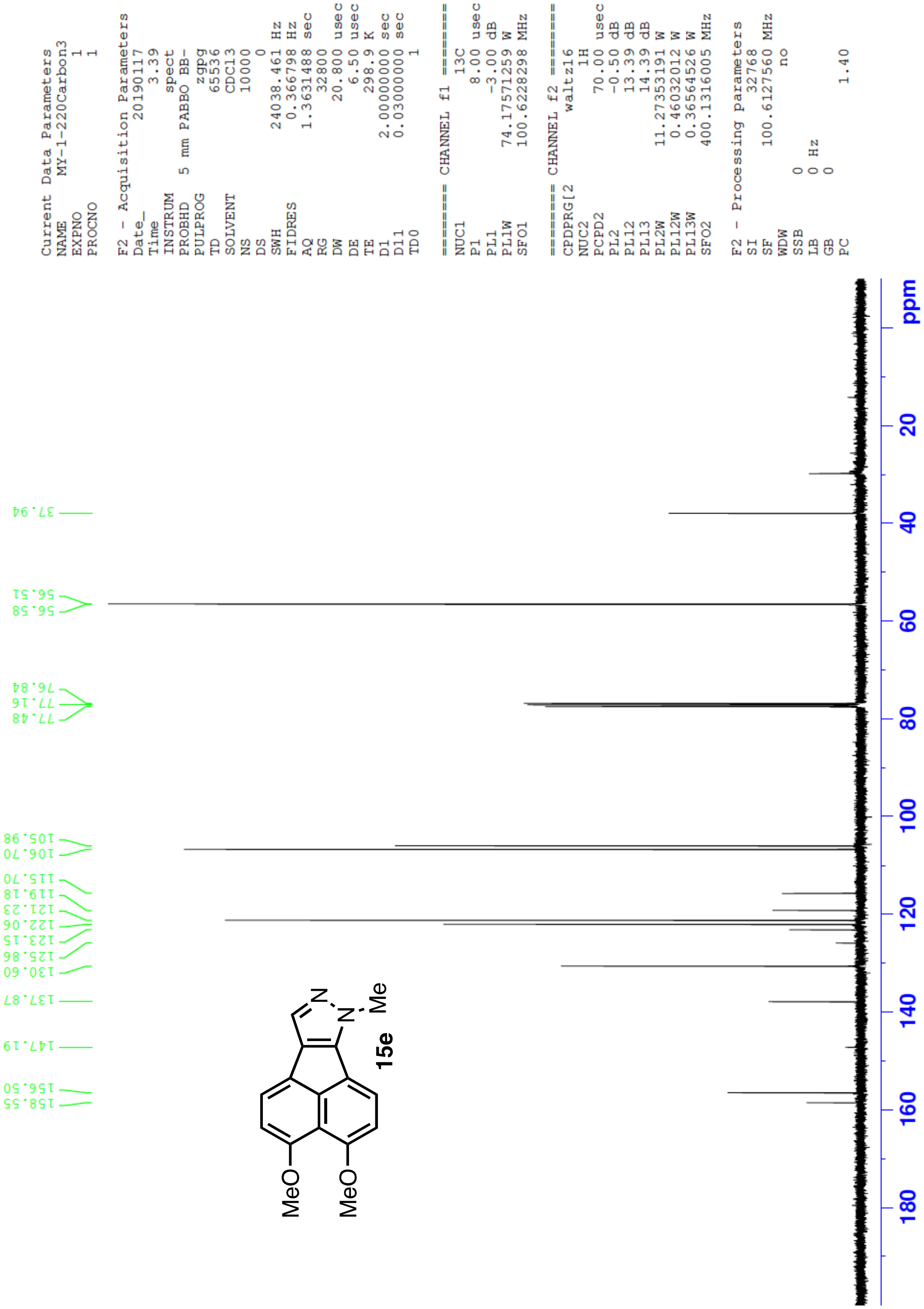

Figure S13. $^{13}C\{^1H\}$ NMR spectrum of **15e** in $CDCl_3$.

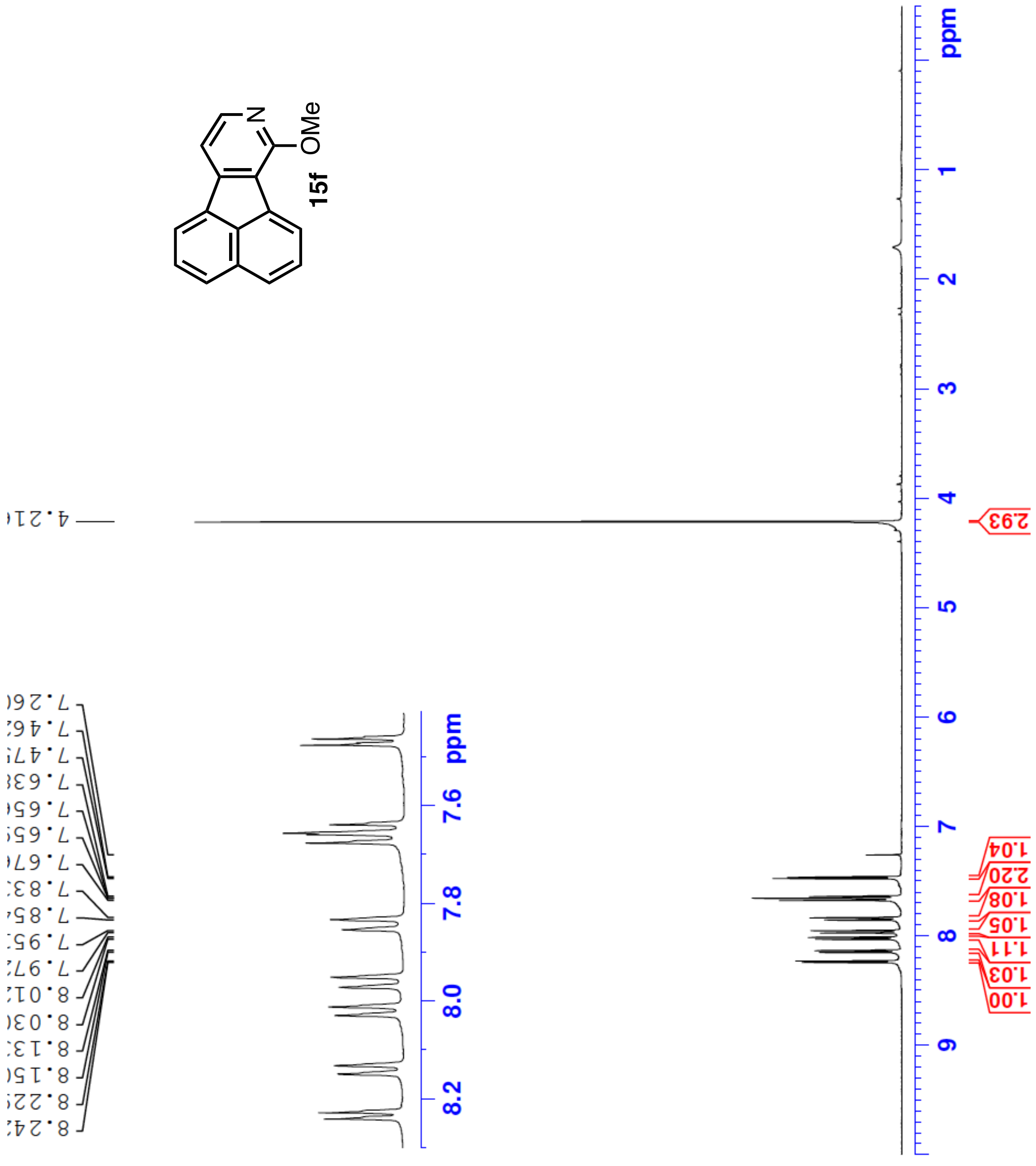

Figure S14. ^{1}H NMR spectrum of **15f** in $CDCl_3$.

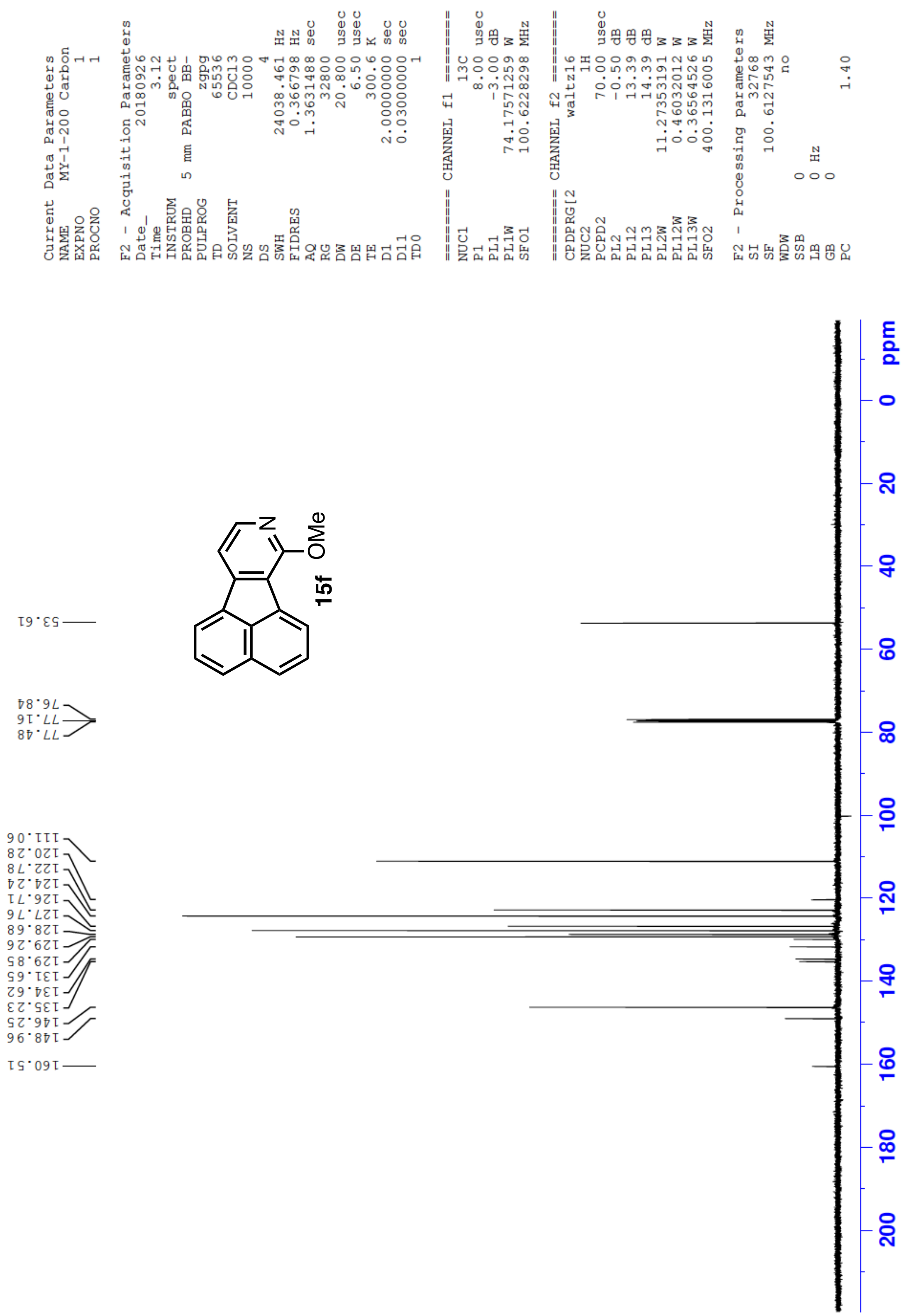

Figure S15. $^{13}C\{^{1}H\}$ NMR spectrum of **15f** in $CDCl_3$.

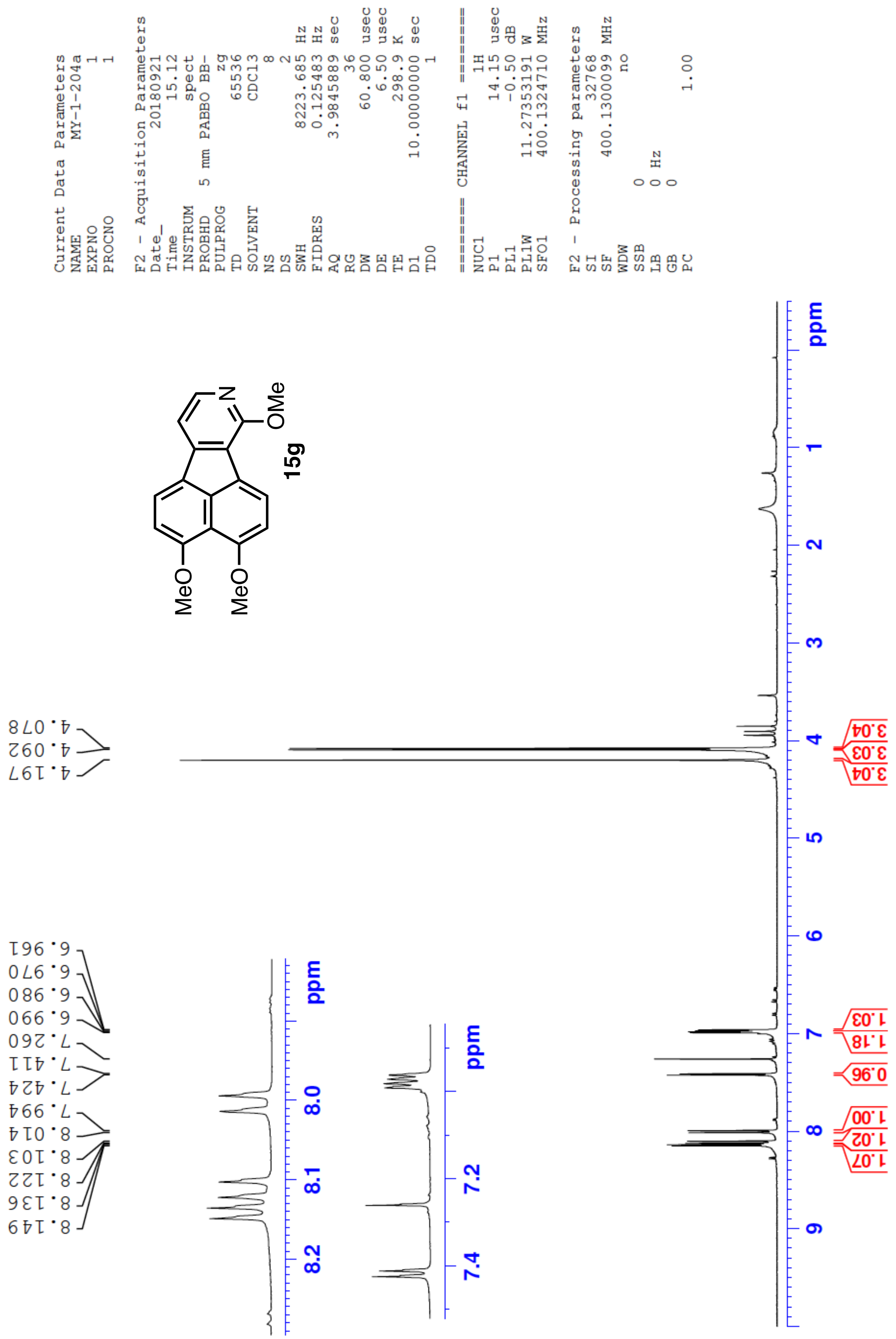

Figure S16. ^{1}H NMR spectrum of **15g** in $CDCl_3$.

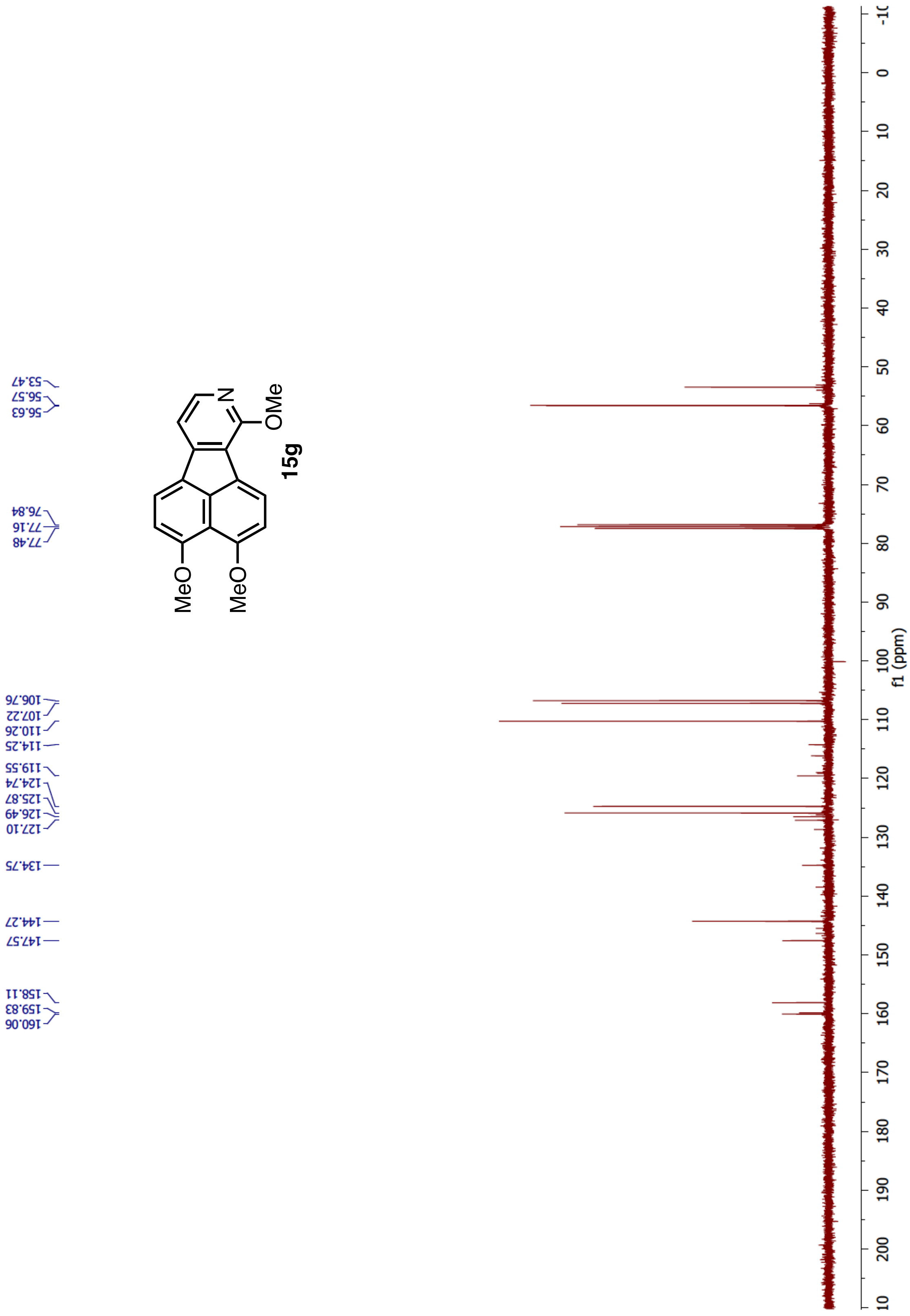

Figure S17. $^{13}C\{^{1}H\}$ NMR spectrum of **15g** in $CDCl_3$.

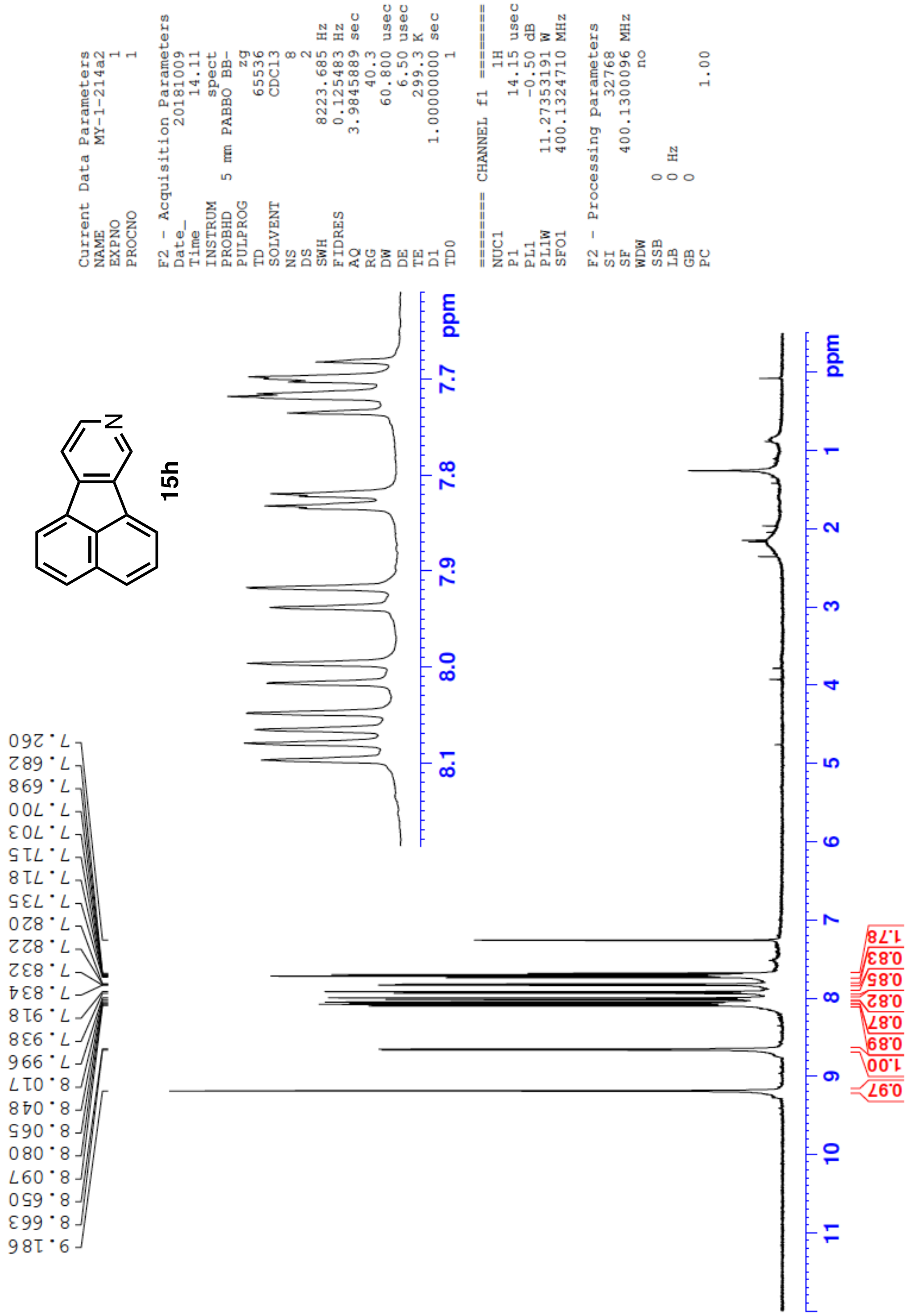

Figure S18. ^{1}H NMR spectrum of **15h** in $CDCl_3$.

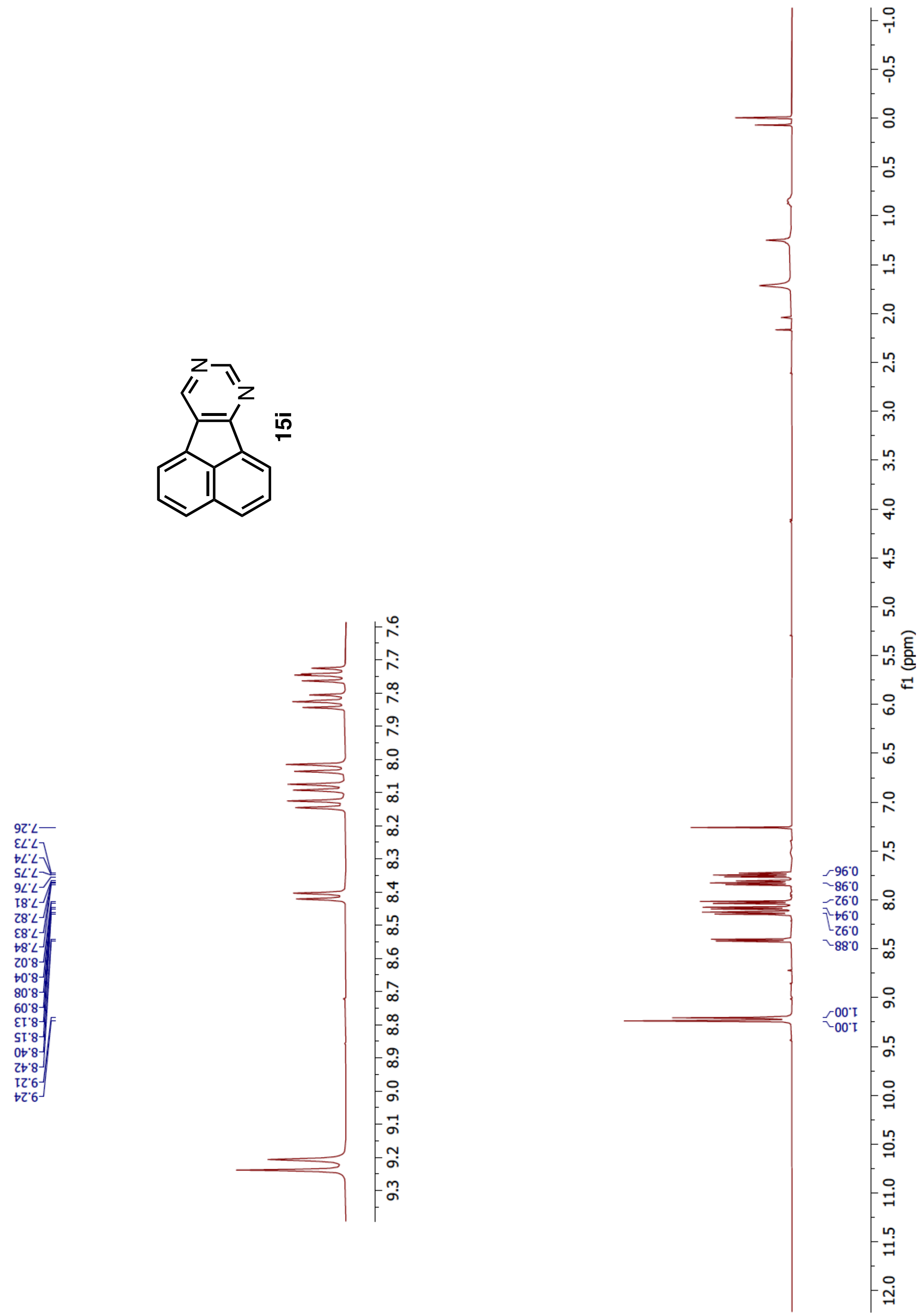

Figure S19. ^{1}H NMR spectrum of **15i** in $CDCl_3$.

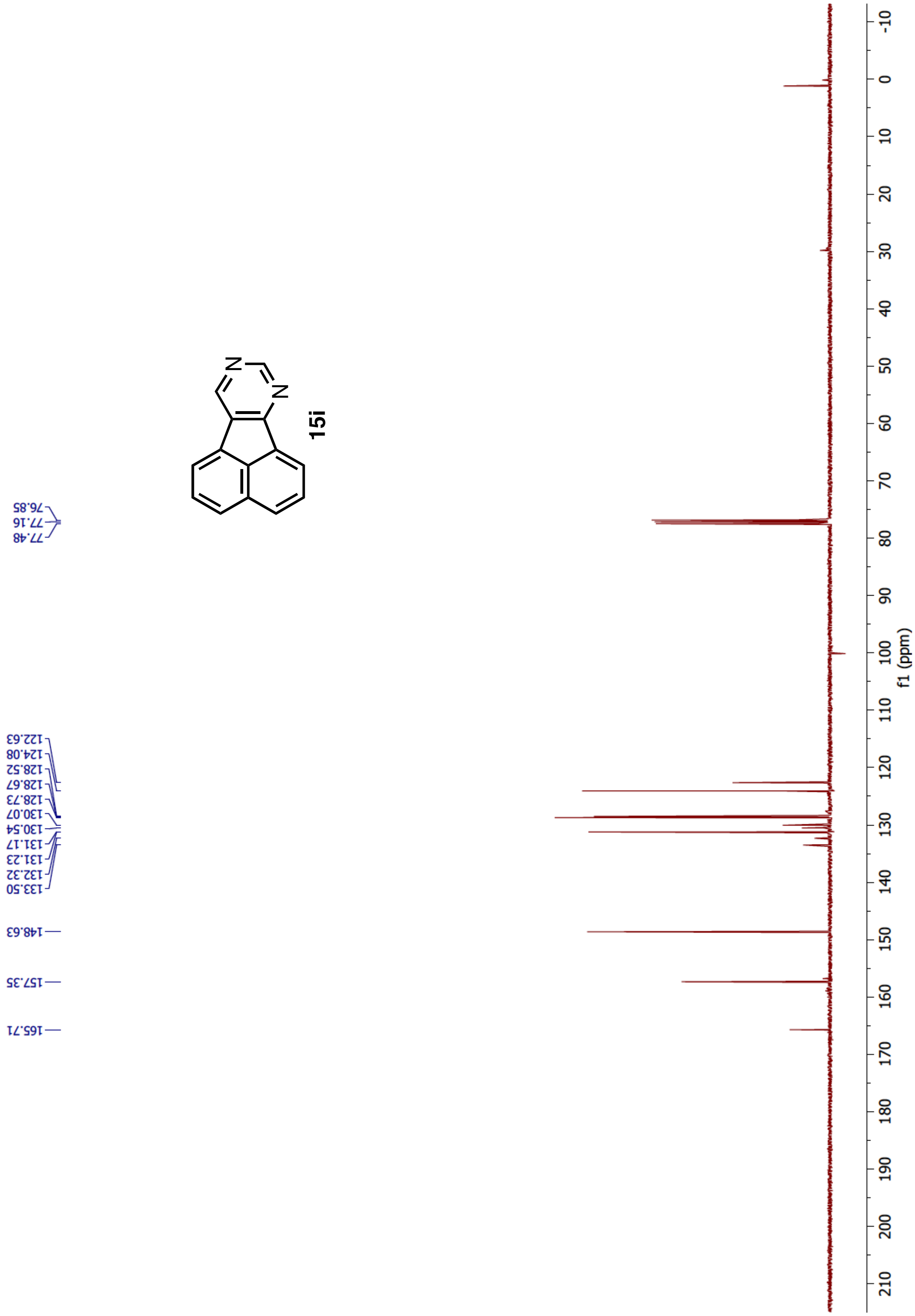

Figure S20. $^{13}C\{^{1}H\}$ NMR spectrum of **15i** in $CDCl_3$.

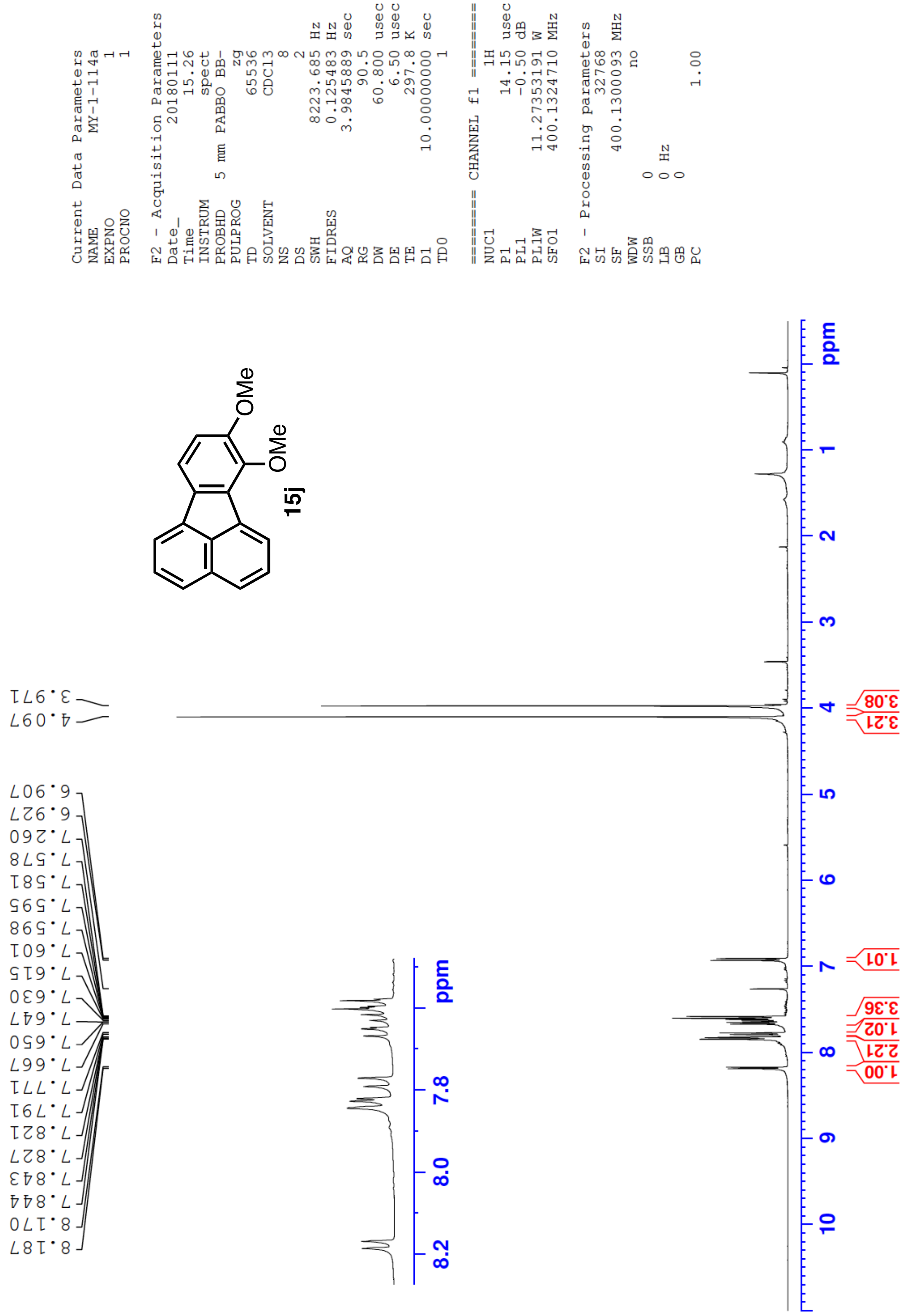

Figure S21. ^{1}H NMR spectrum of **15j** in $CDCl_3$.

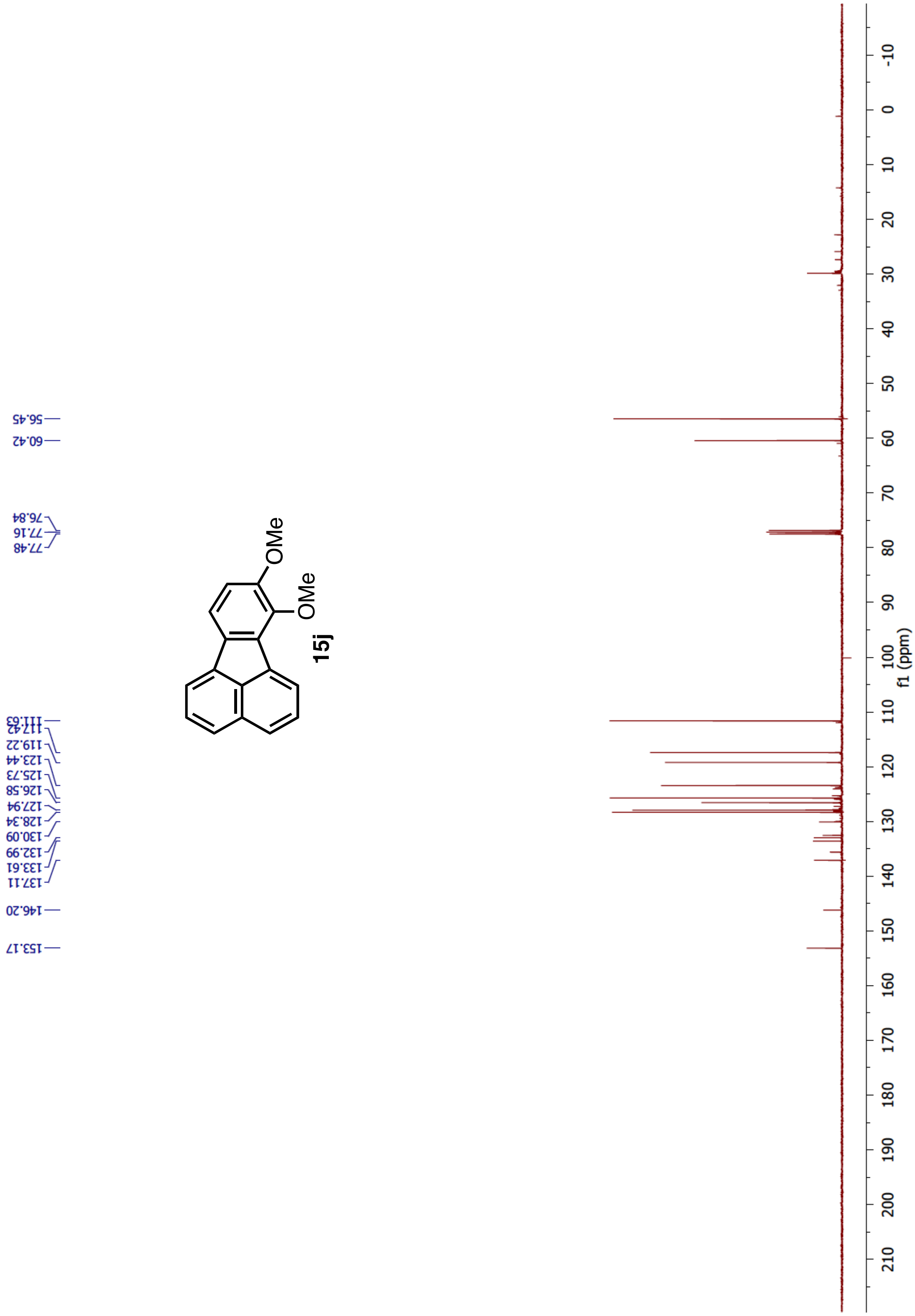

Figure S22. $^{13}C\{^{1}H\}$ NMR spectrum of **15j** in $CDCl_3$.

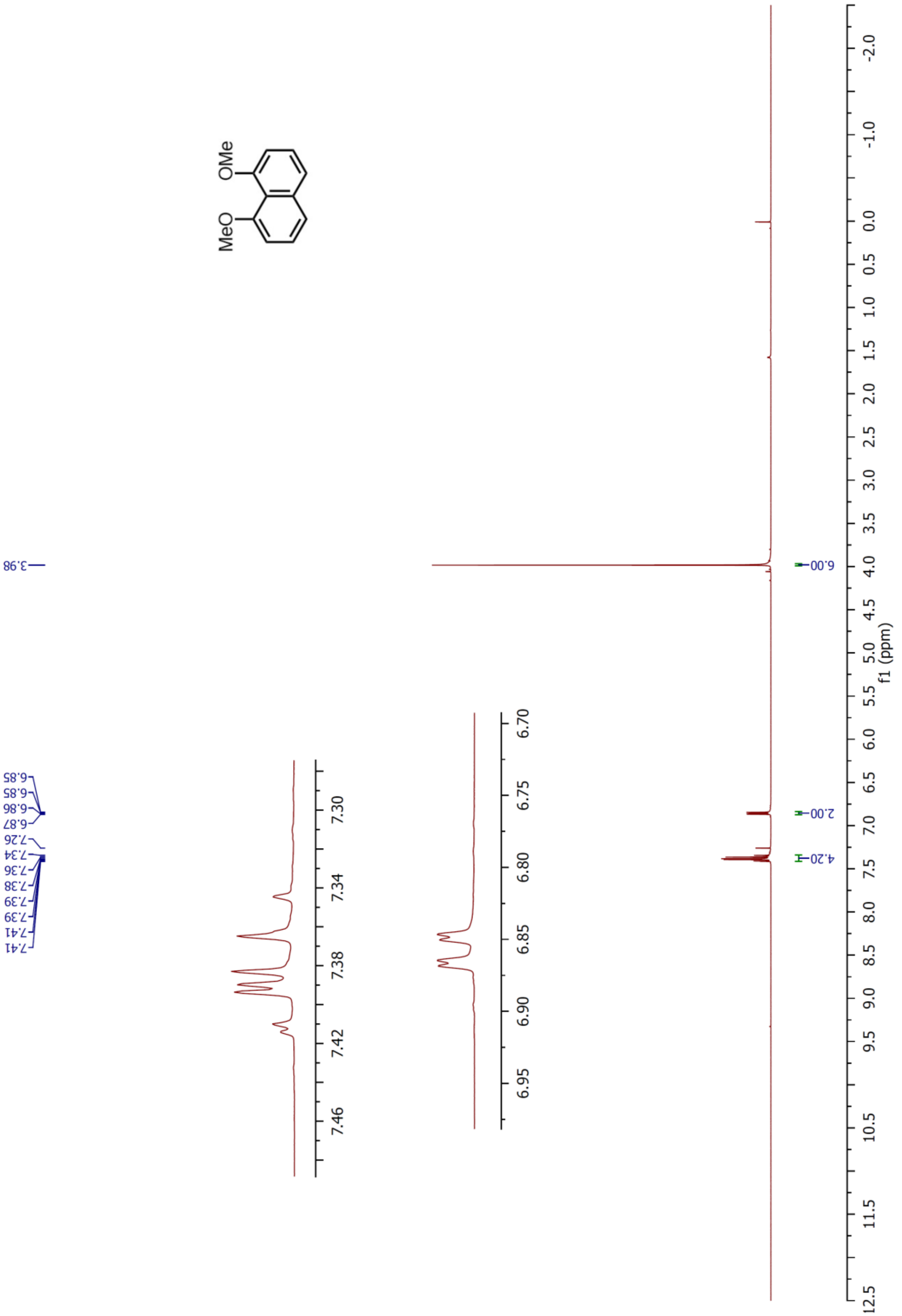

Figure S23. ^{1}H NMR spectrum of **20** in $CDCl_3$.

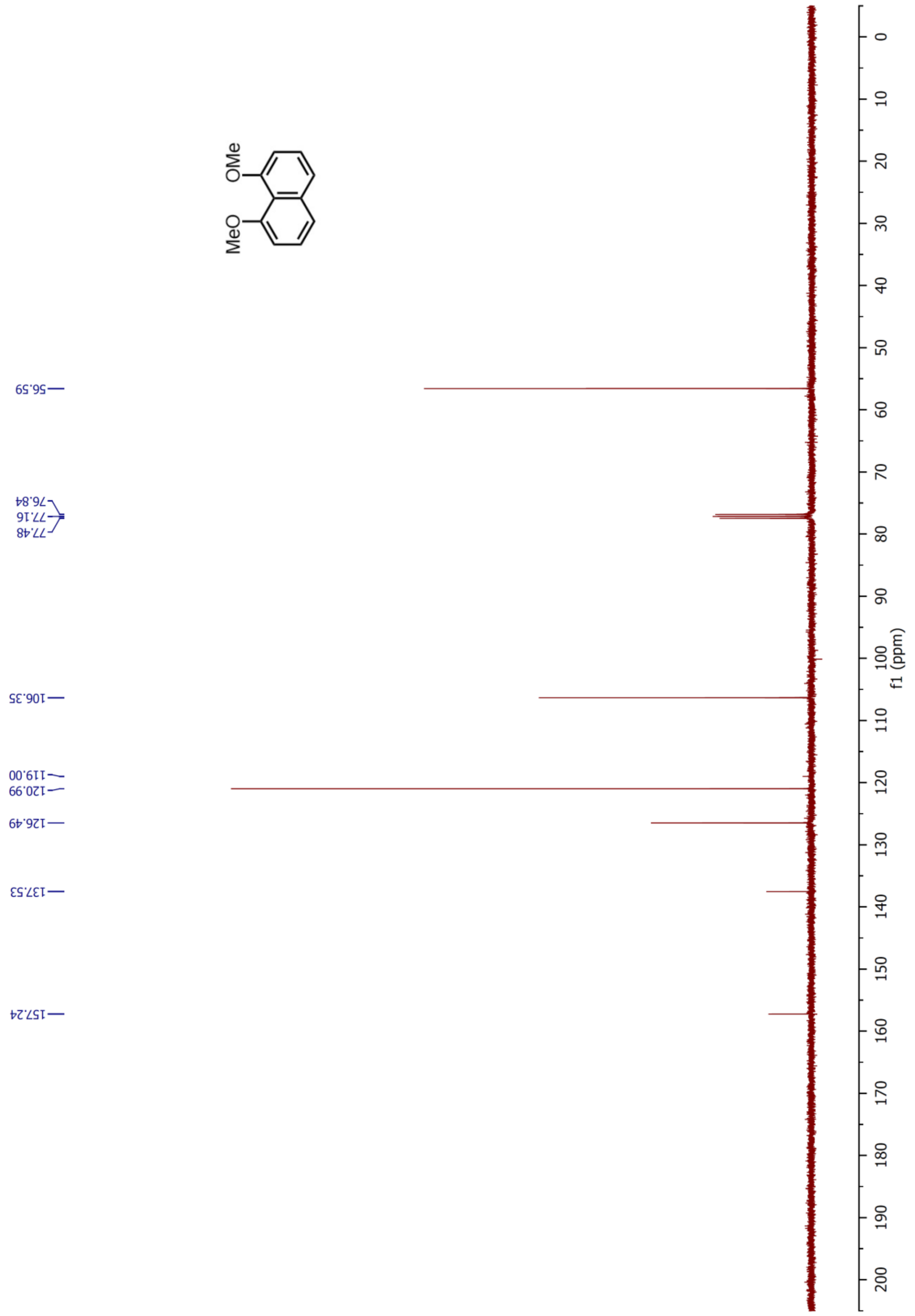

Figure S24. $^{13}C\{^{1}H\}$ NMR spectrum of **20** in $CDCl_3$.

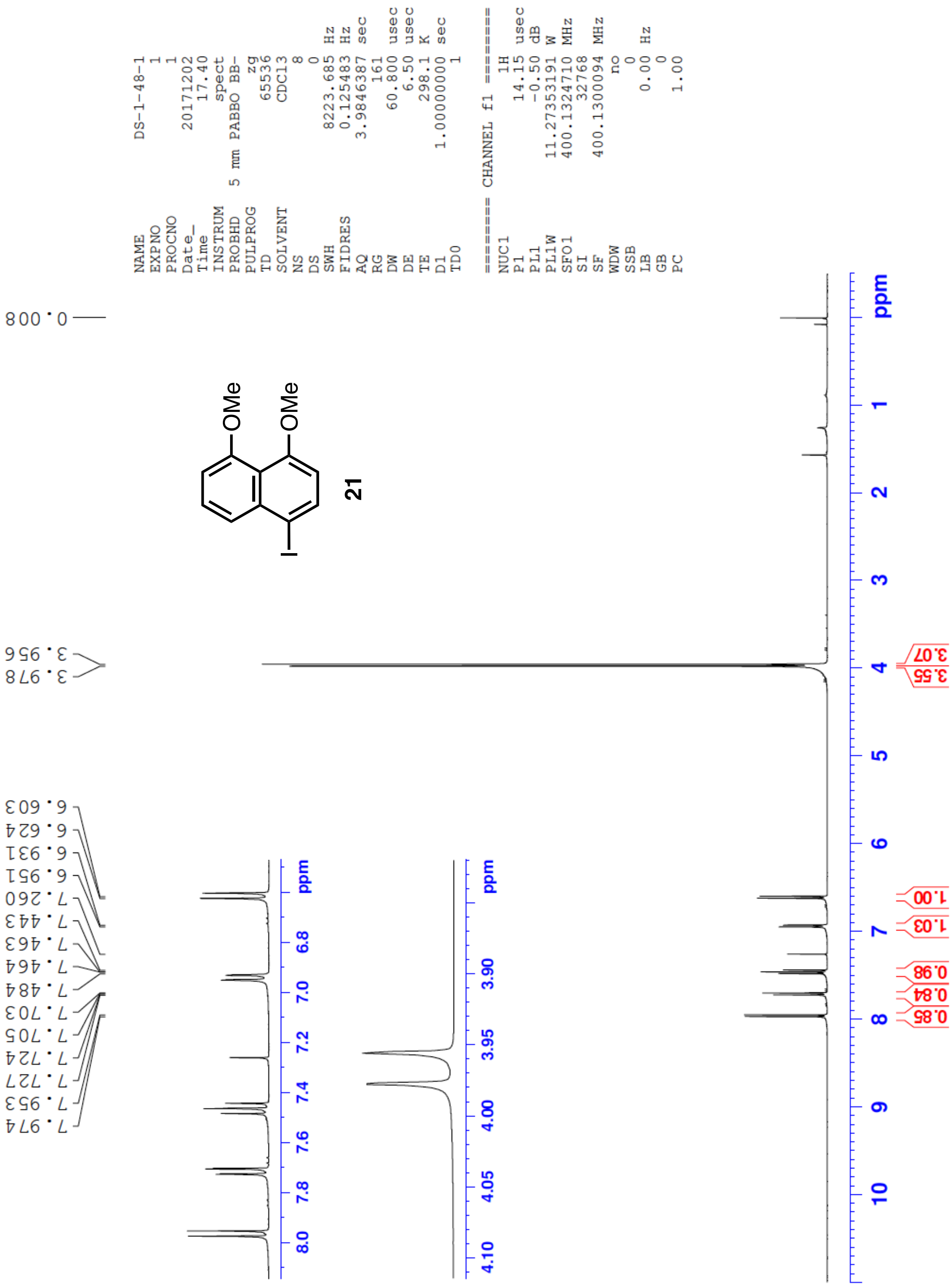

Figure S25. ^{1}H NMR spectrum of **21** in $CDCl_3$.

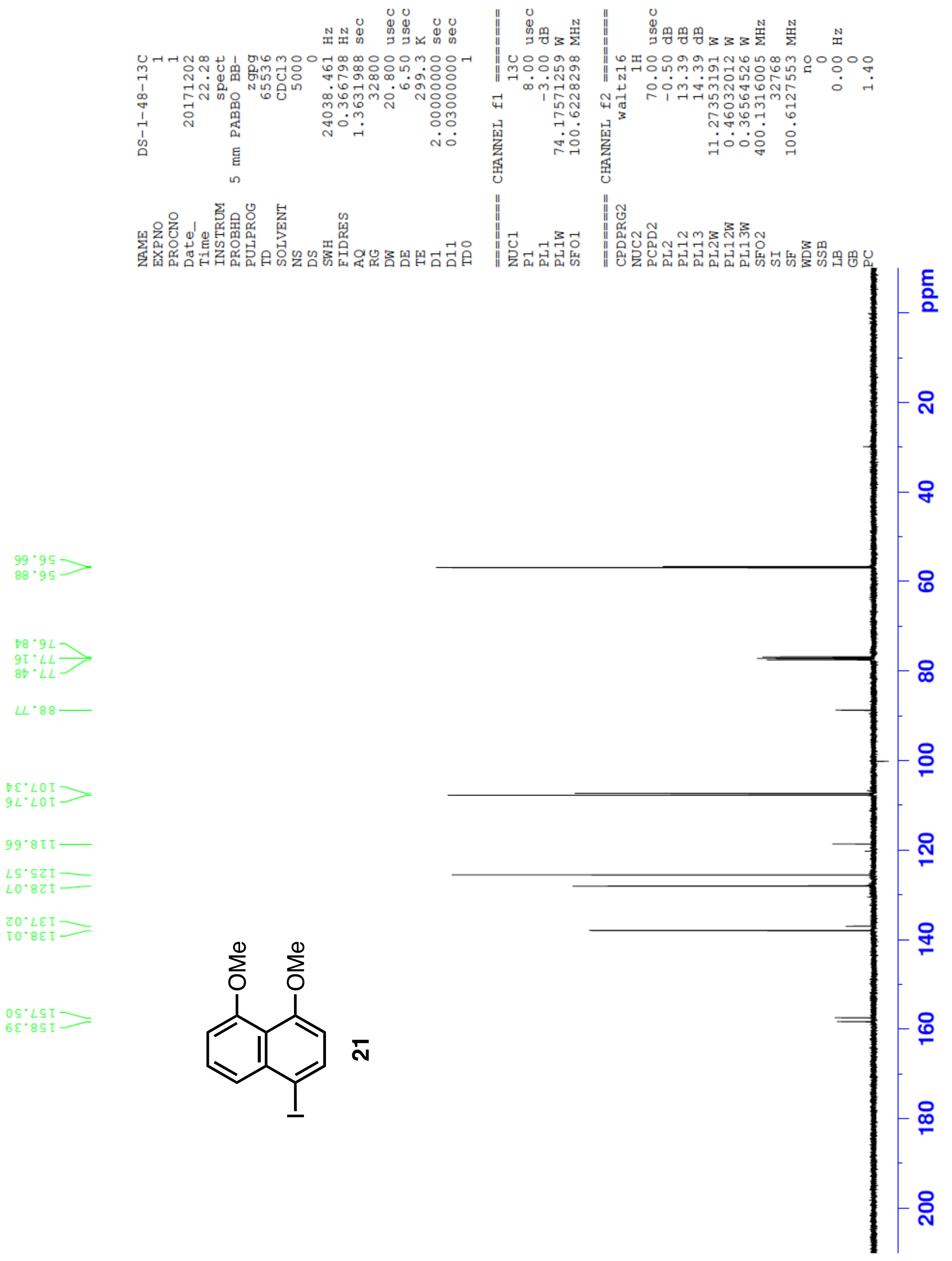

Figure S26. $^{13}C\{^{1}H\}$ NMR spectrum of **21** in $CDCl_3$.

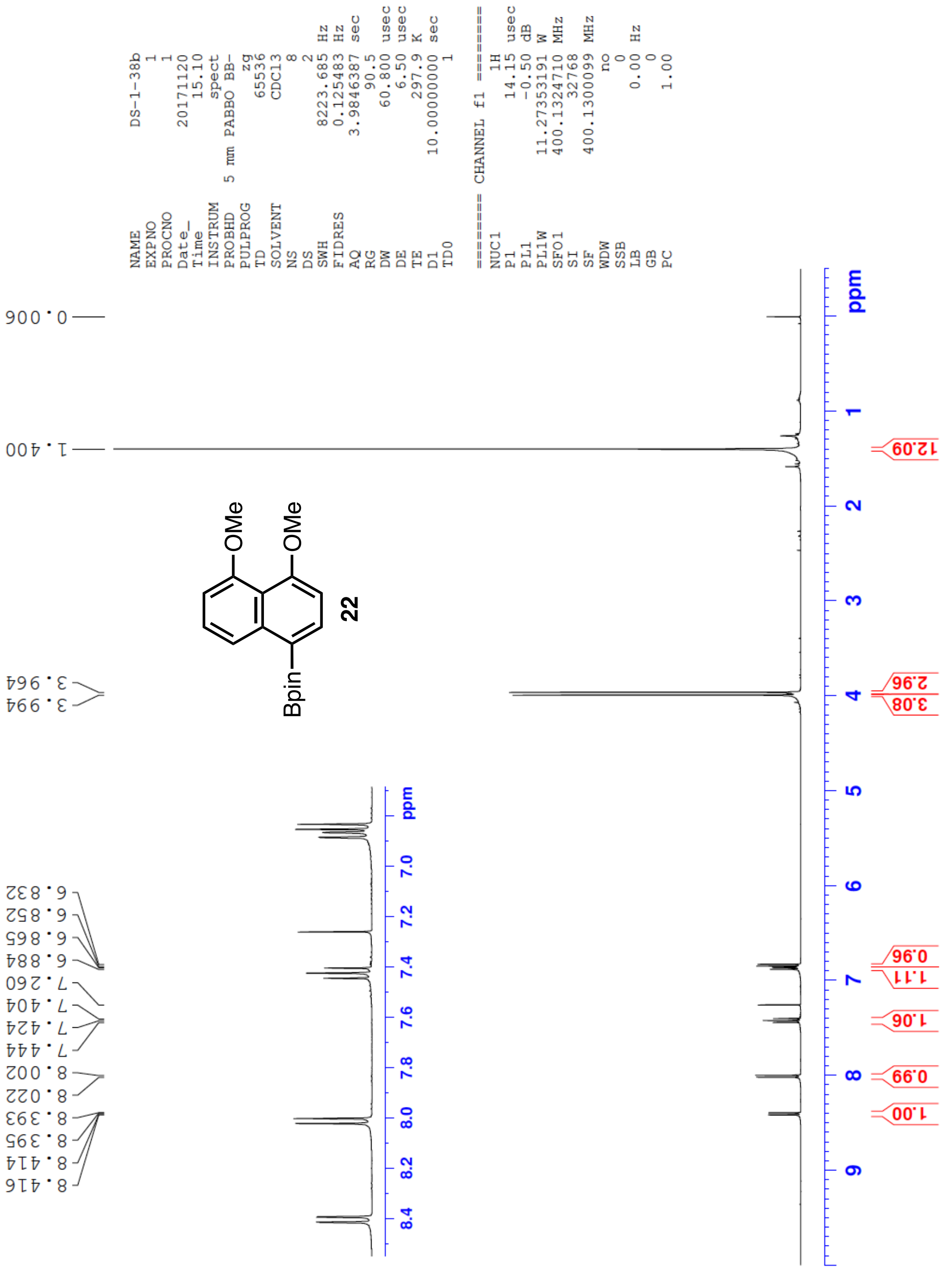

Figure S27. ^1H NMR spectrum of **22** in $CDCl_3$.

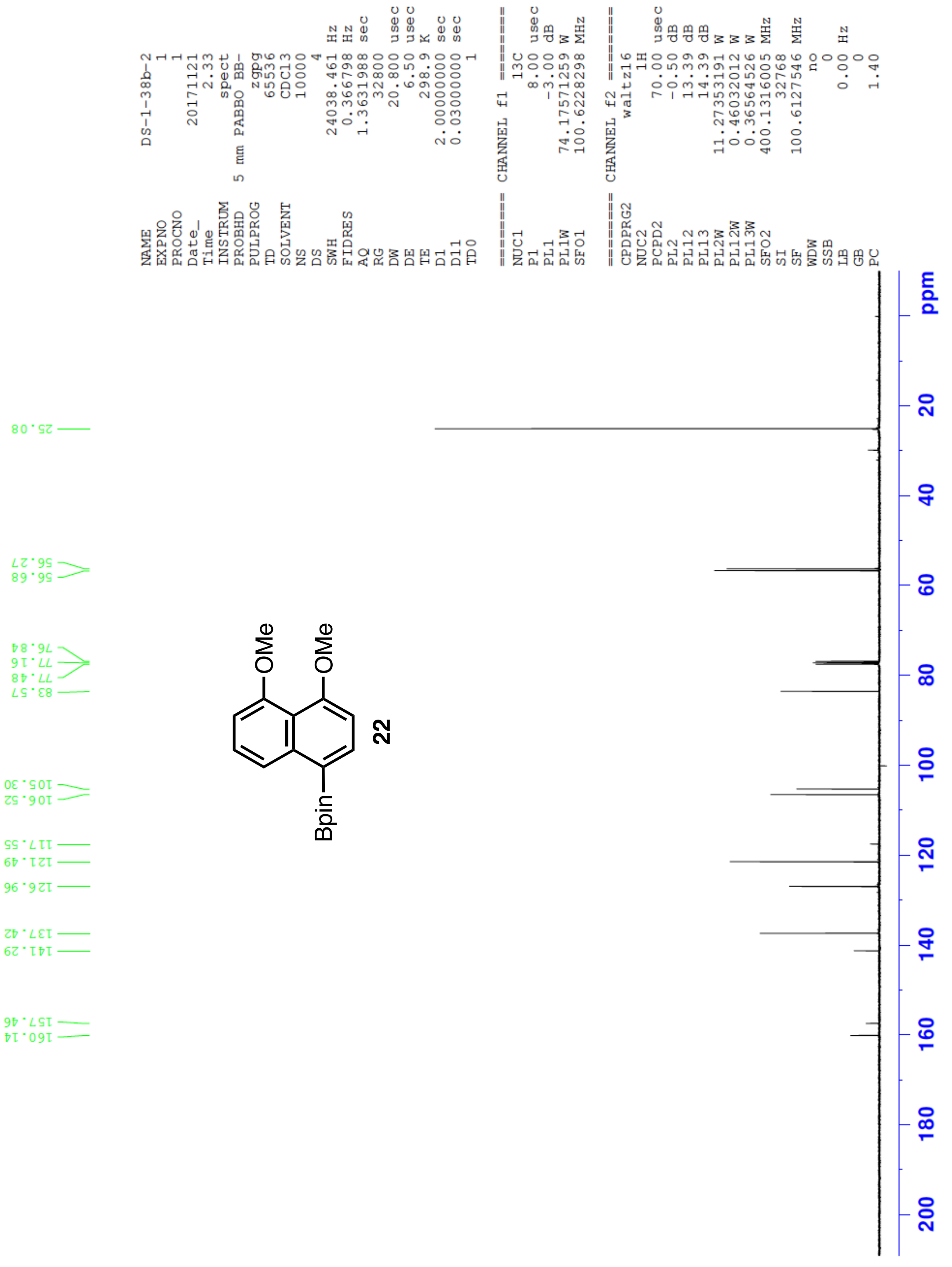

Figure S28. $^{13}C\{^{1}H\}$ NMR spectrum of **22** in $CDCl_3$.

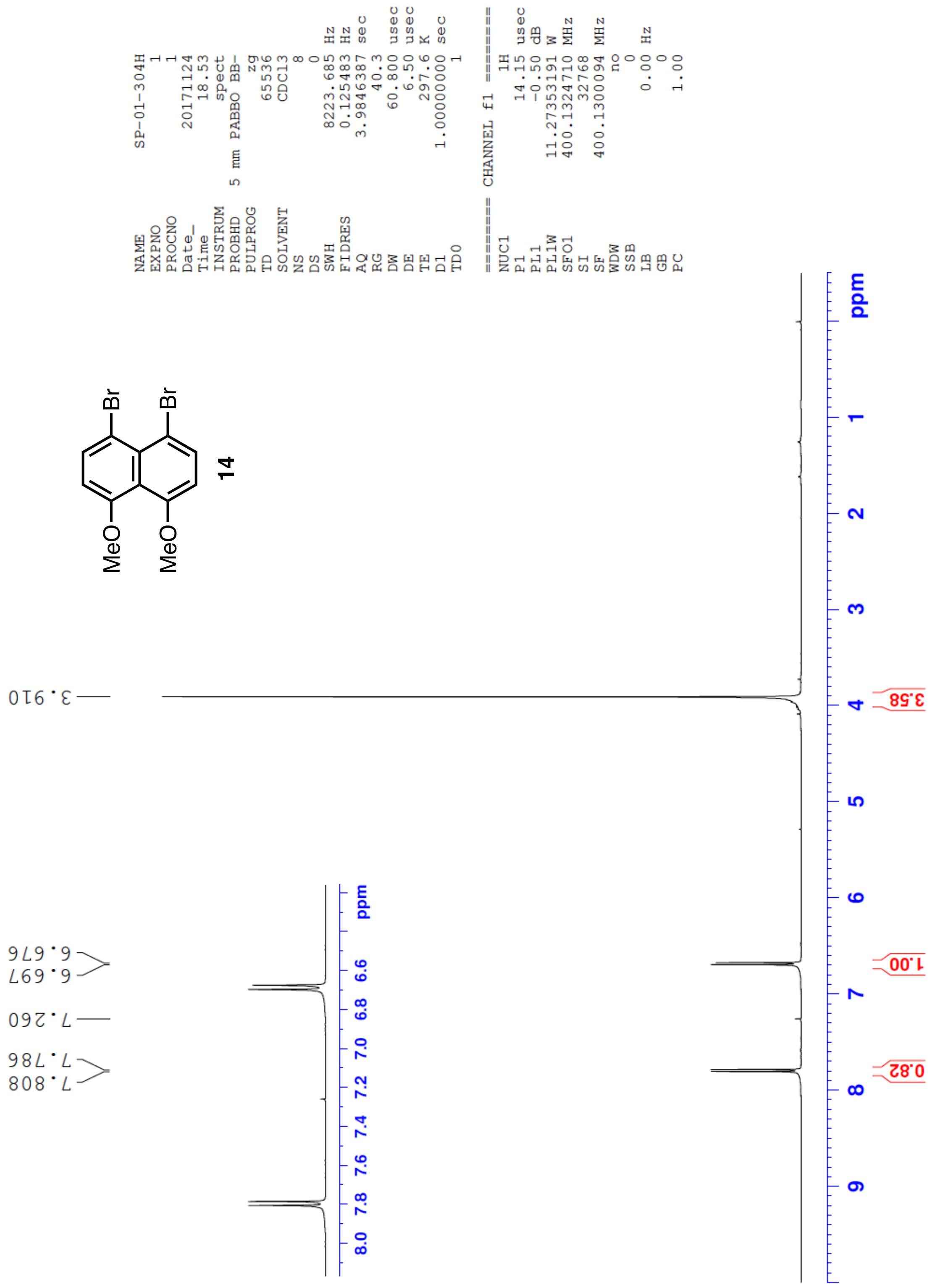

Figure S29. ^{1}H NMR spectrum of **14** in $CDCl_3$.

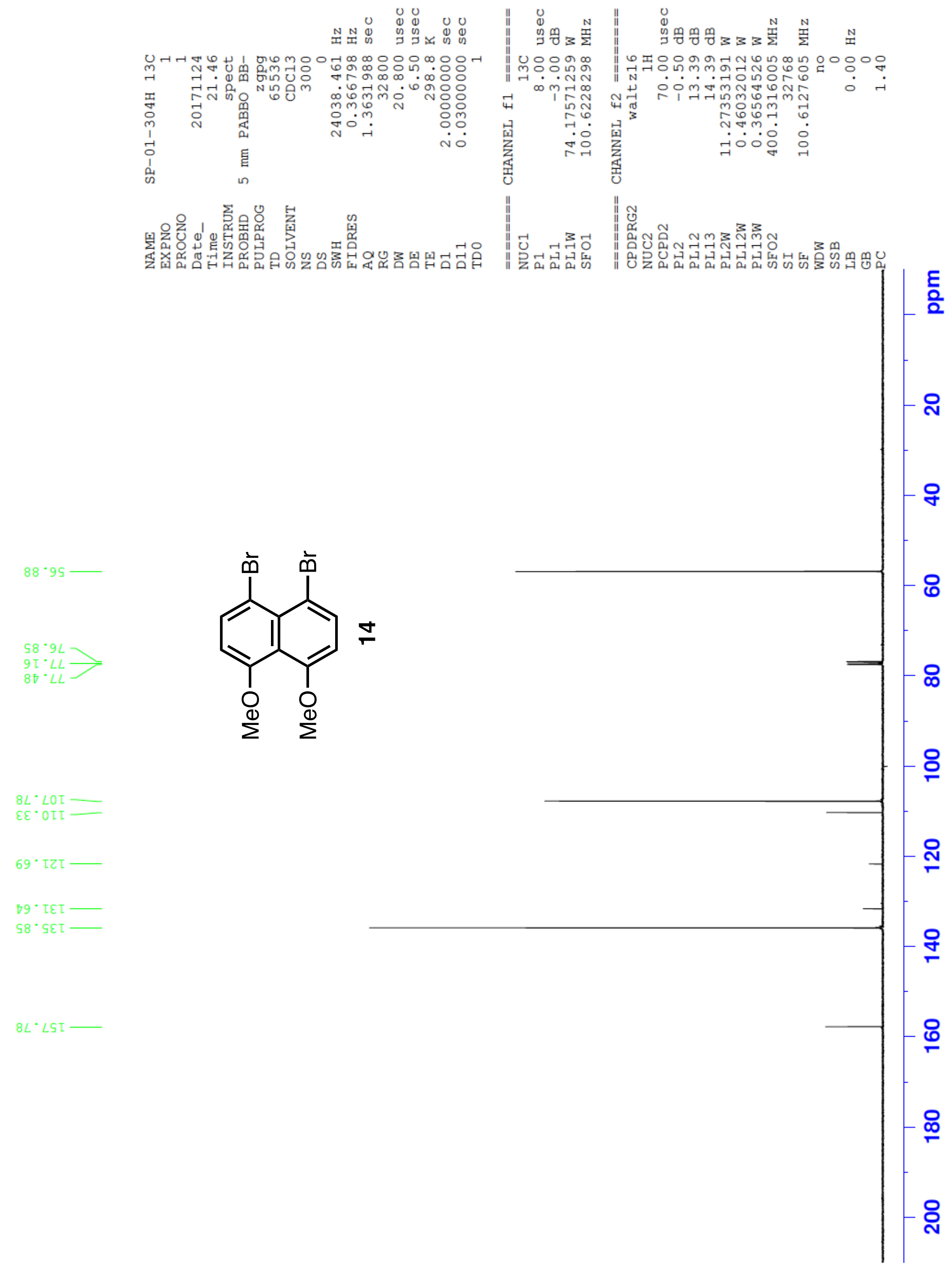

Figure S30. $^{13}C\{^1H\}$ NMR spectrum of **14** in $CDCl_3$.

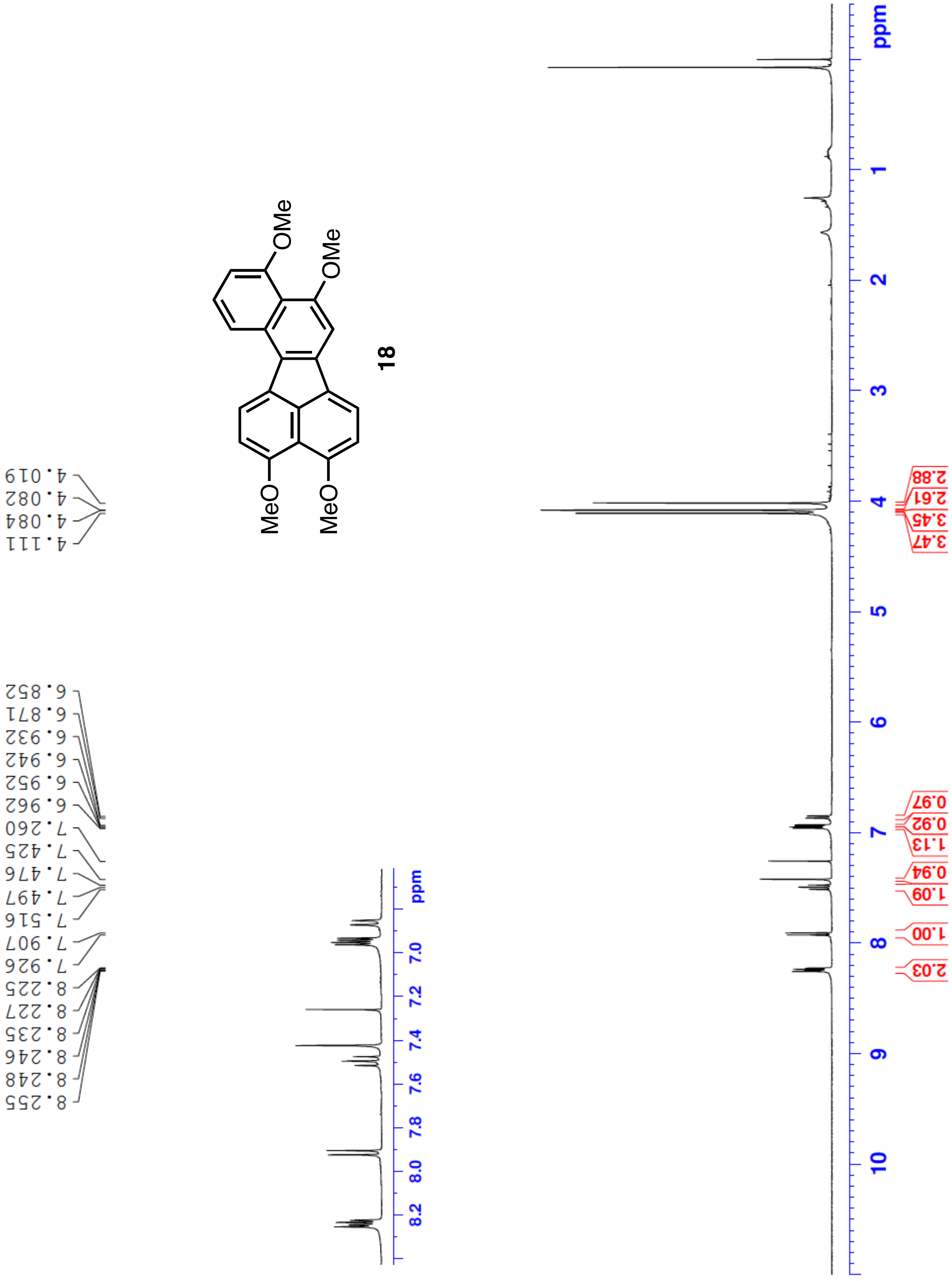

Figure S31. ^{1}H NMR spectrum of **18** in $CDCl_3$.

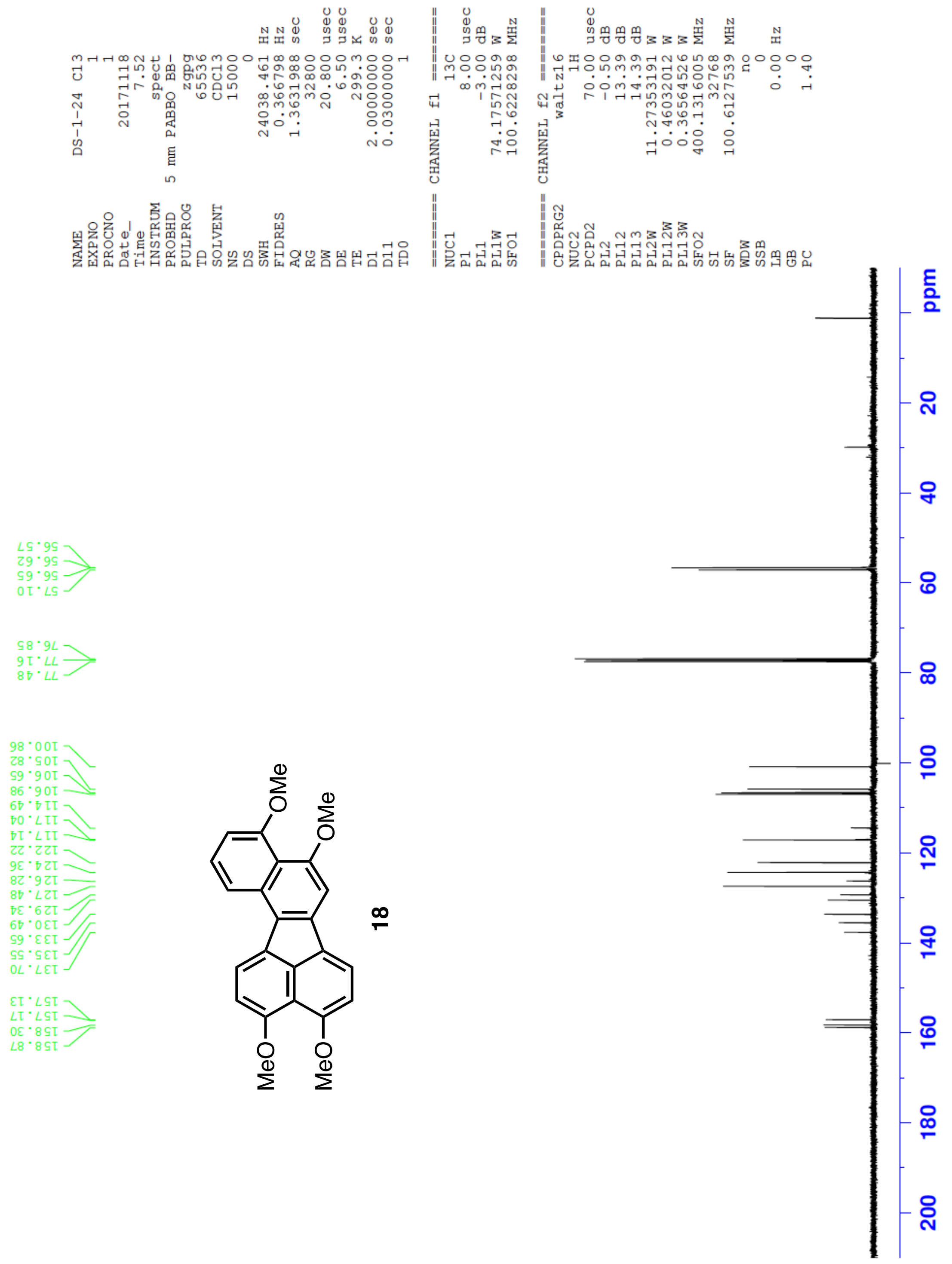

Figure S32. $^{13}C\{^1H\}$ NMR spectrum of **18** in $CDCl_3$.

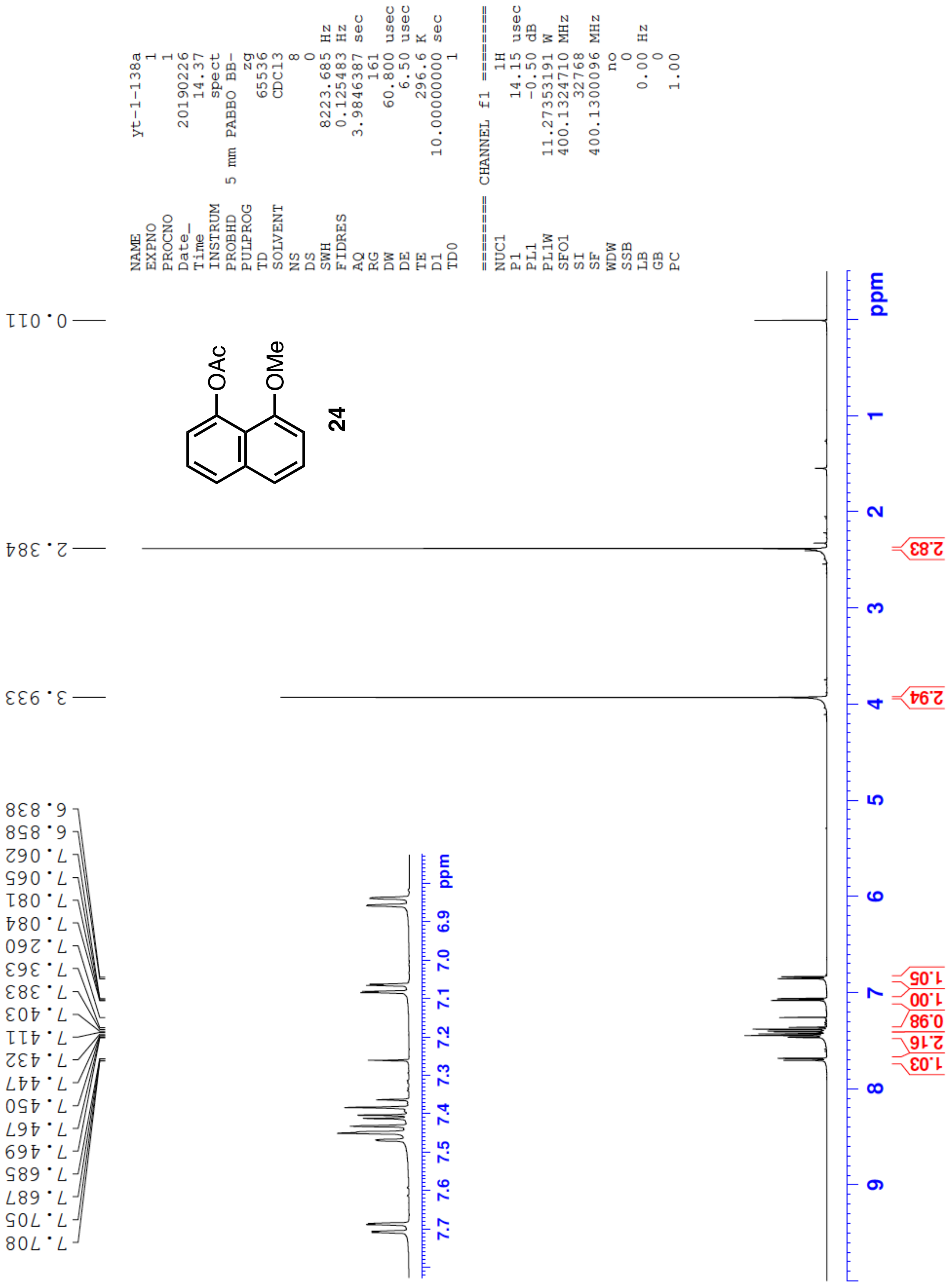

Figure S33. ^{1}H NMR spectrum of **24** in $CDCl_3$.

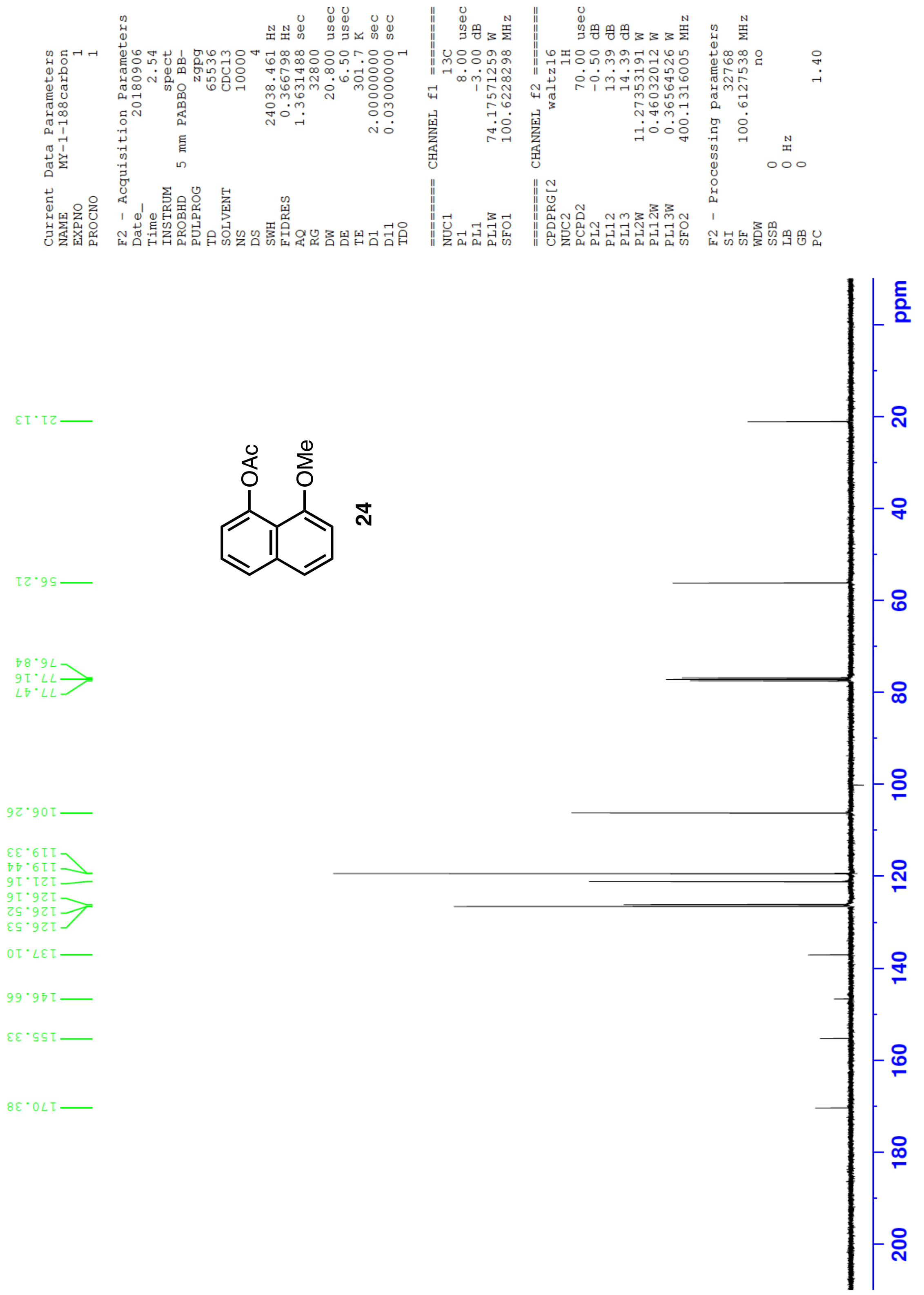

Figure S34. $^{13}C\{^{1}H\}$ NMR spectrum of **24** in $CDCl_3$.

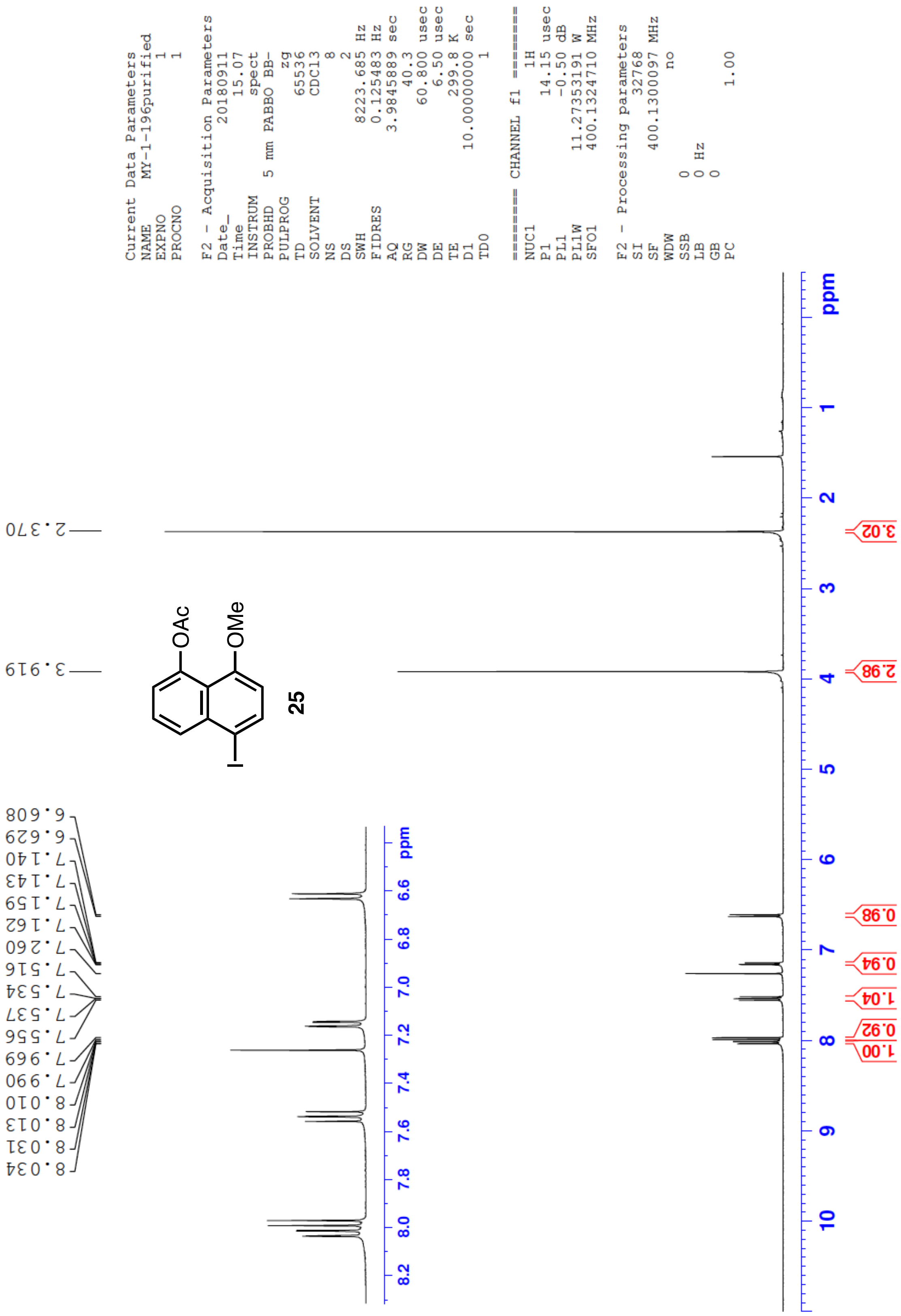

Figure S35. ^{1}H NMR spectrum of **25** in $CDCl_3$.

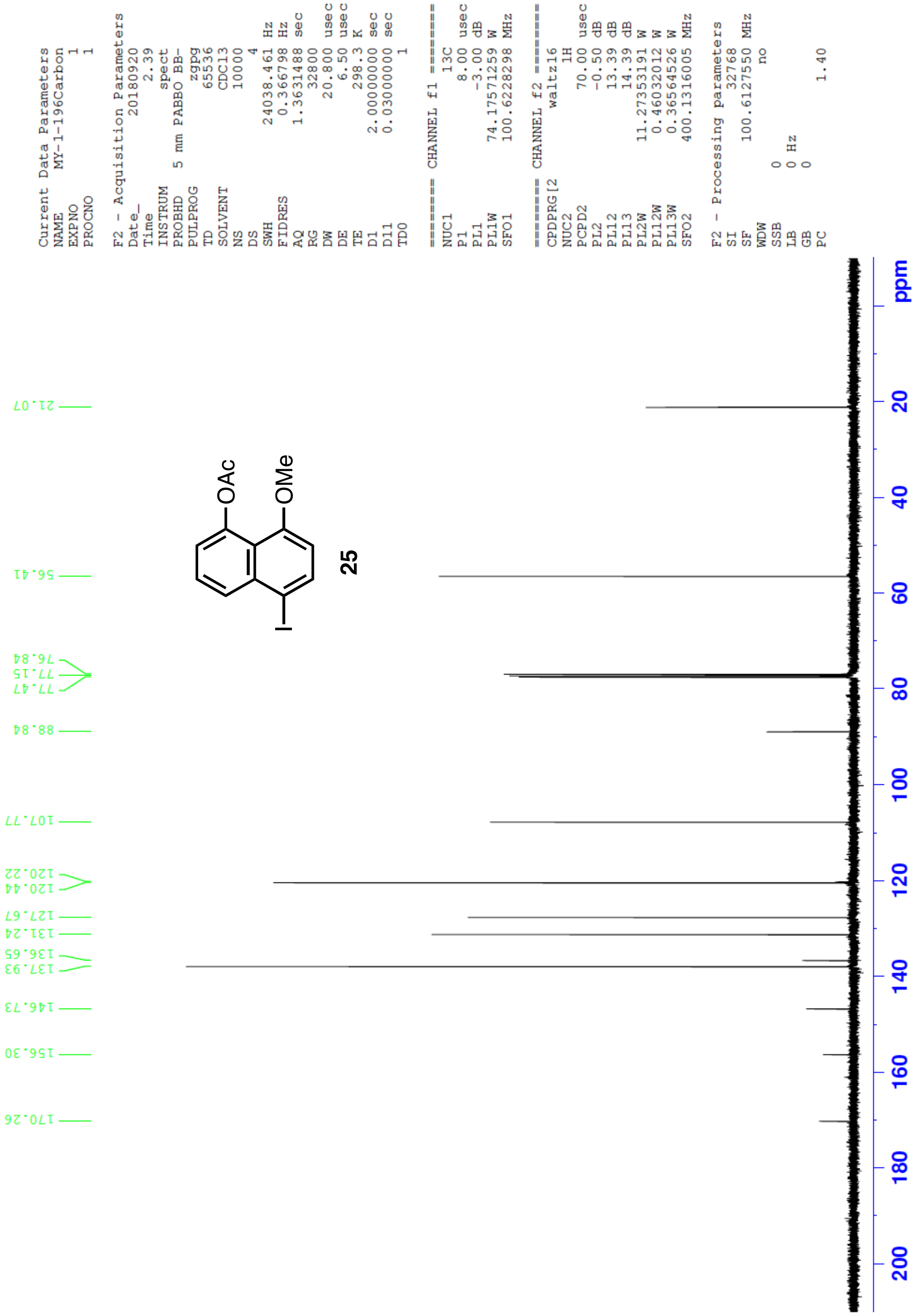

Figure S36. $^{13}C\{^{1}H\}$ NMR spectrum of **25** in $CDCl_3$.

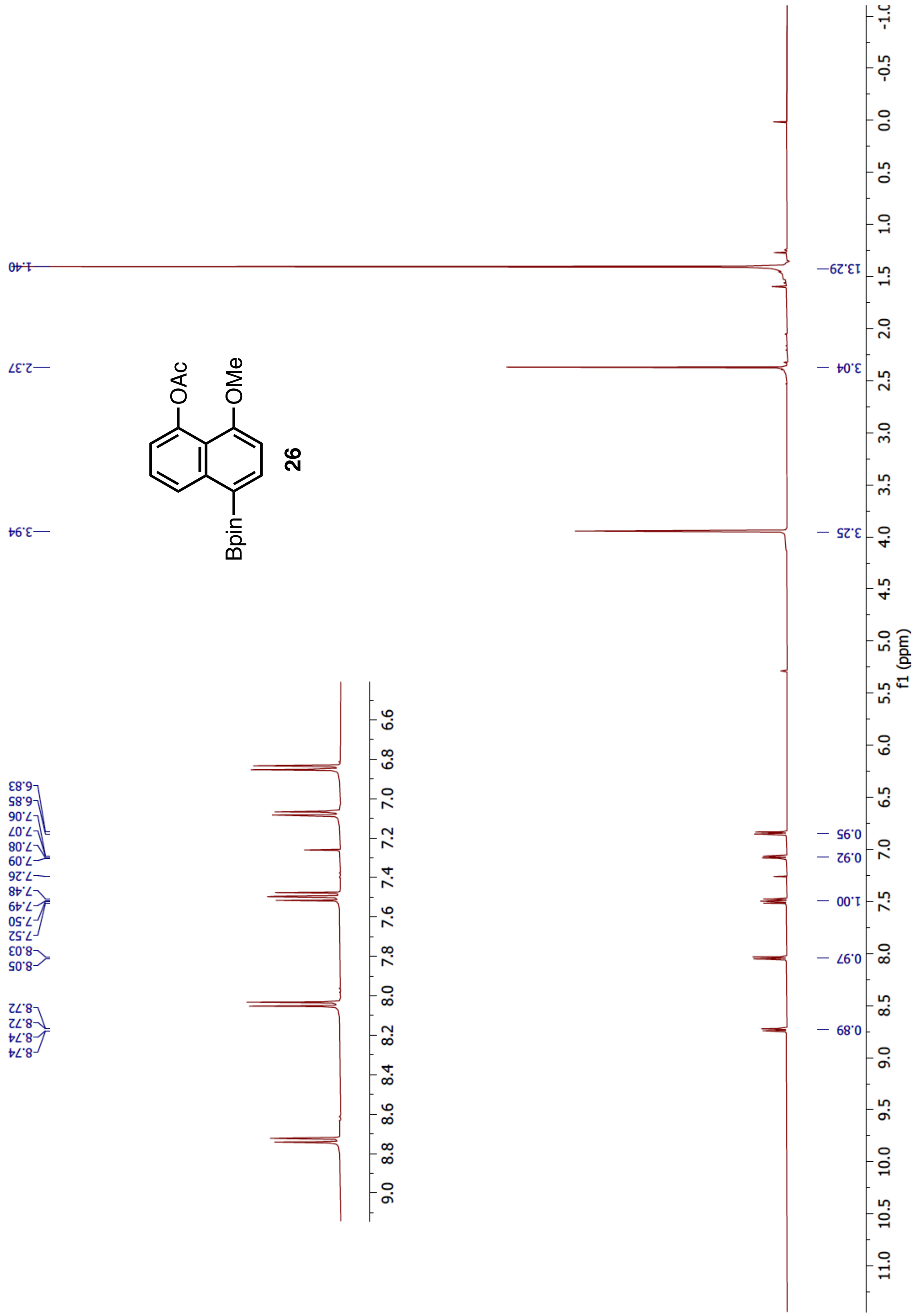

Figure S37. ¹H NMR spectrum of **26** in $CDCl_3$.

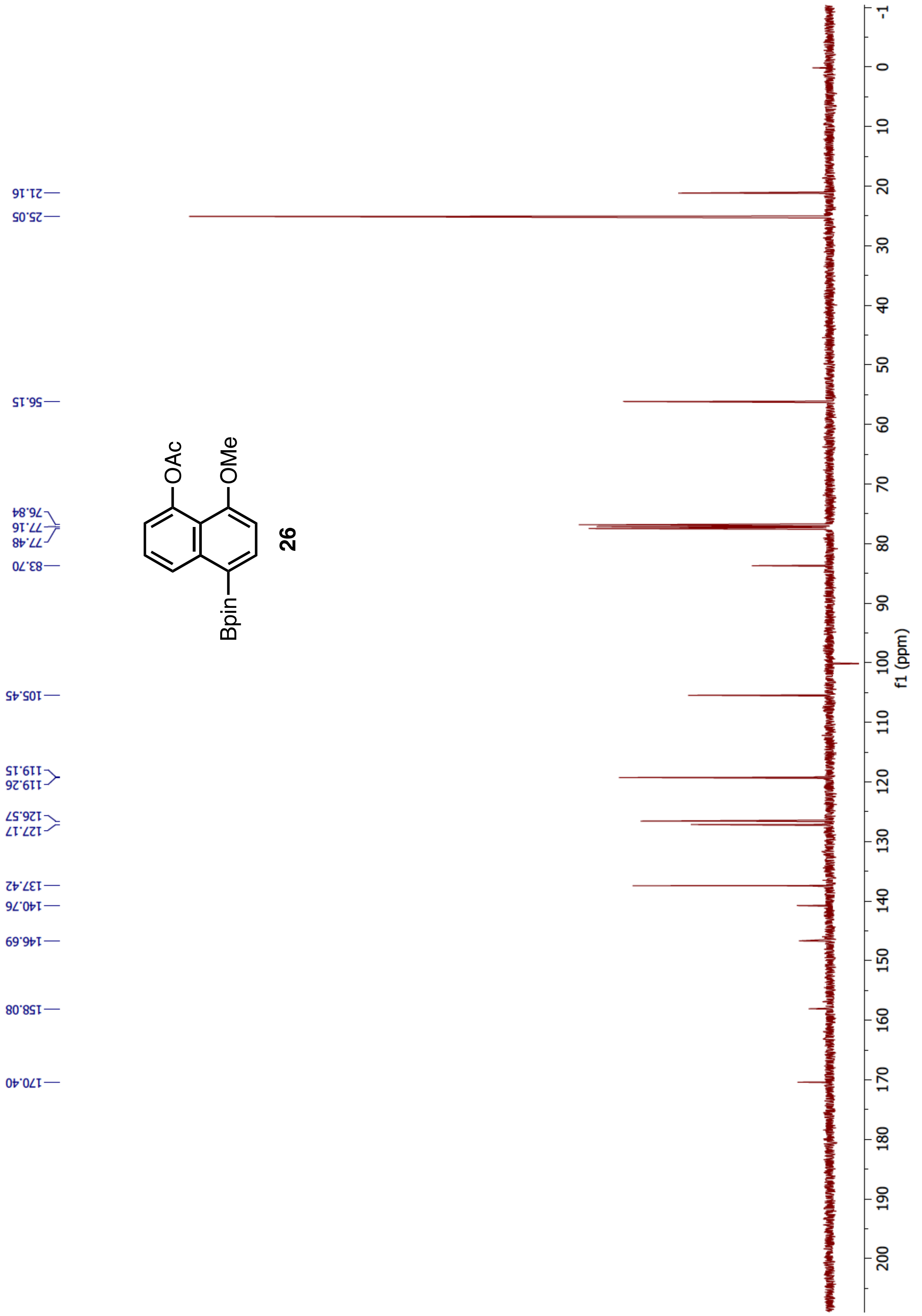

Figure S38. $^{13}C\{^1H\}$ NMR spectrum of **26** in $CDCl_3$.

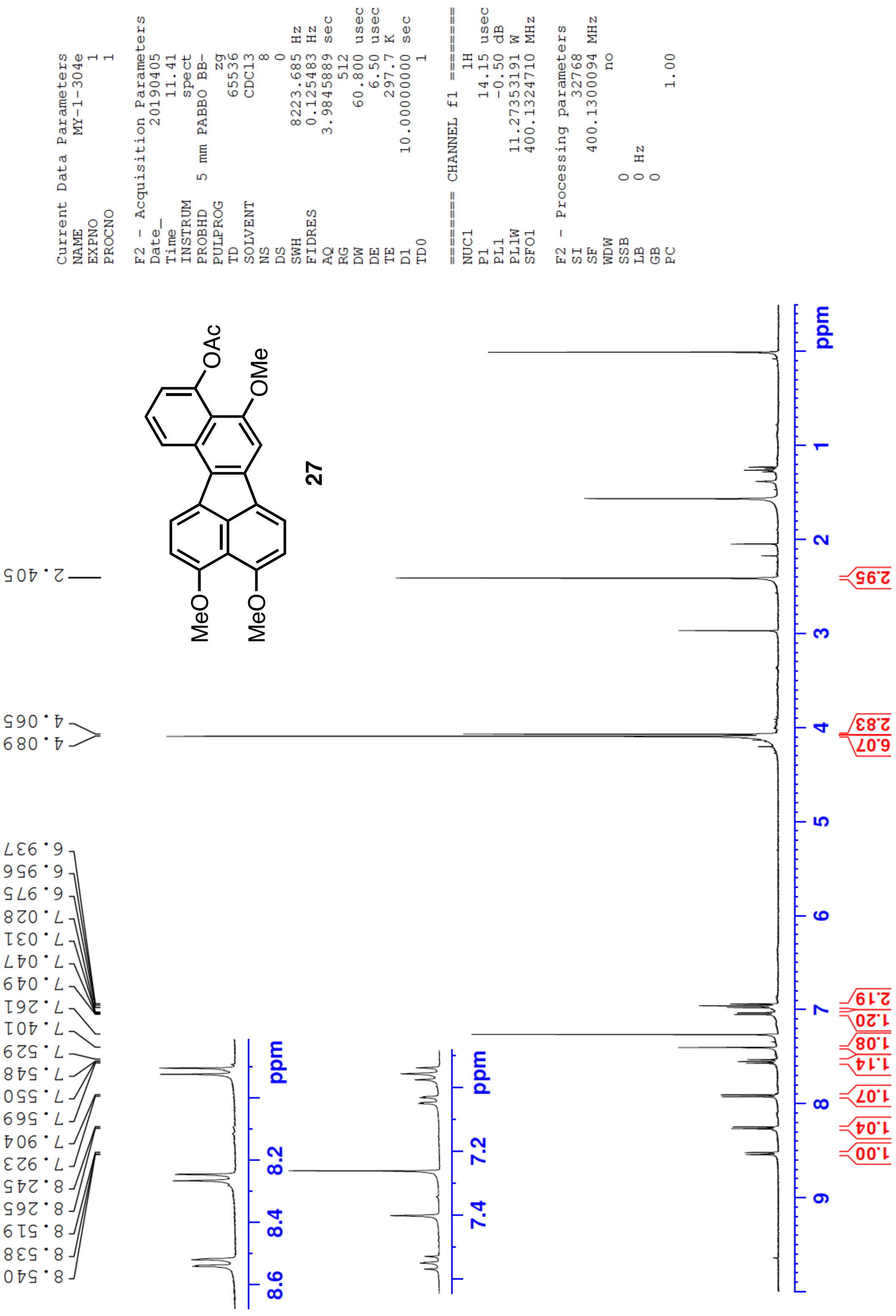

Figure S39. ^{1}H NMR spectrum of **27** in $CDCl_3$.

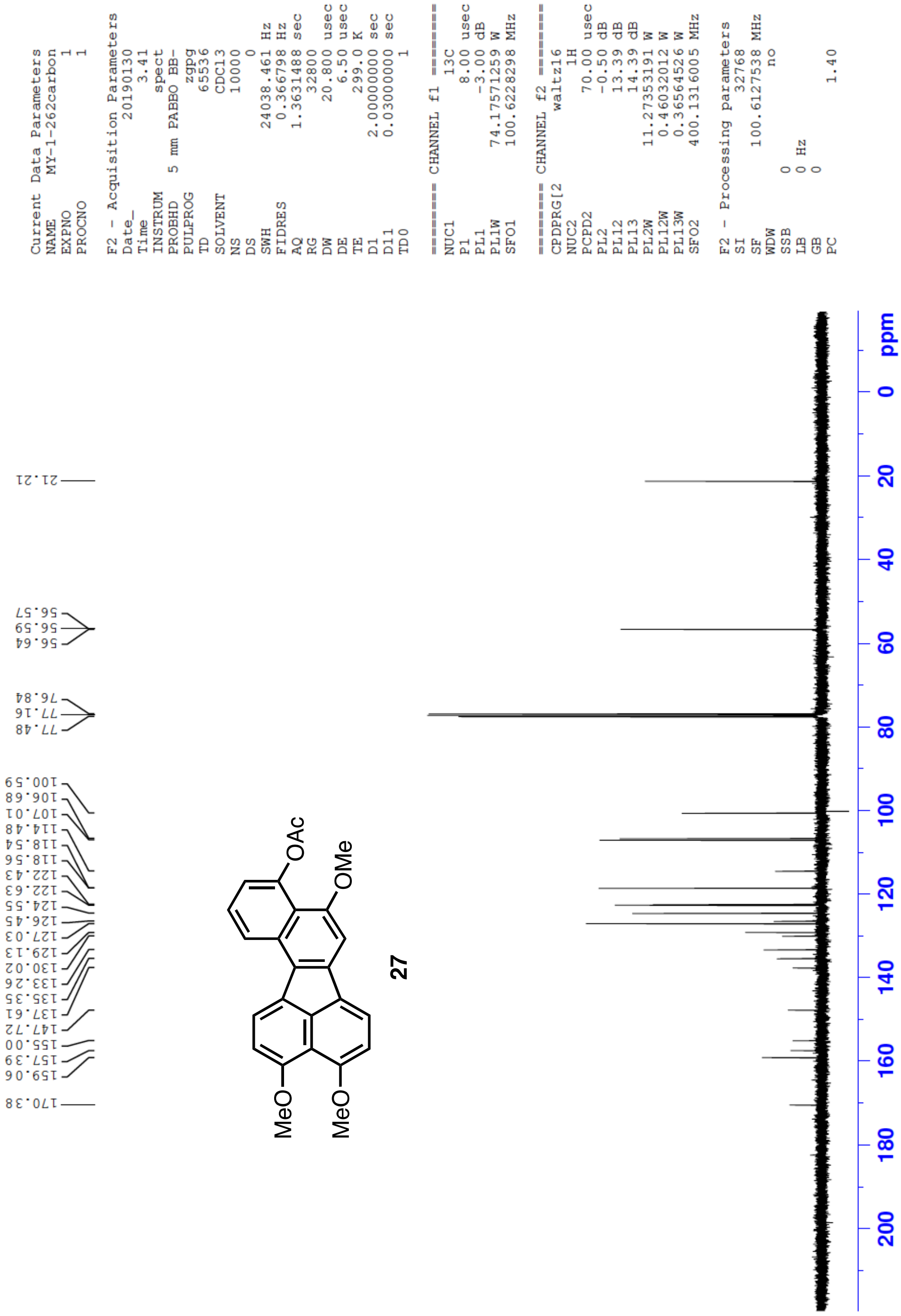

Figure S40. $^{13}C\{^{1}H\}$ NMR spectrum of **27** in $CDCl_3$.

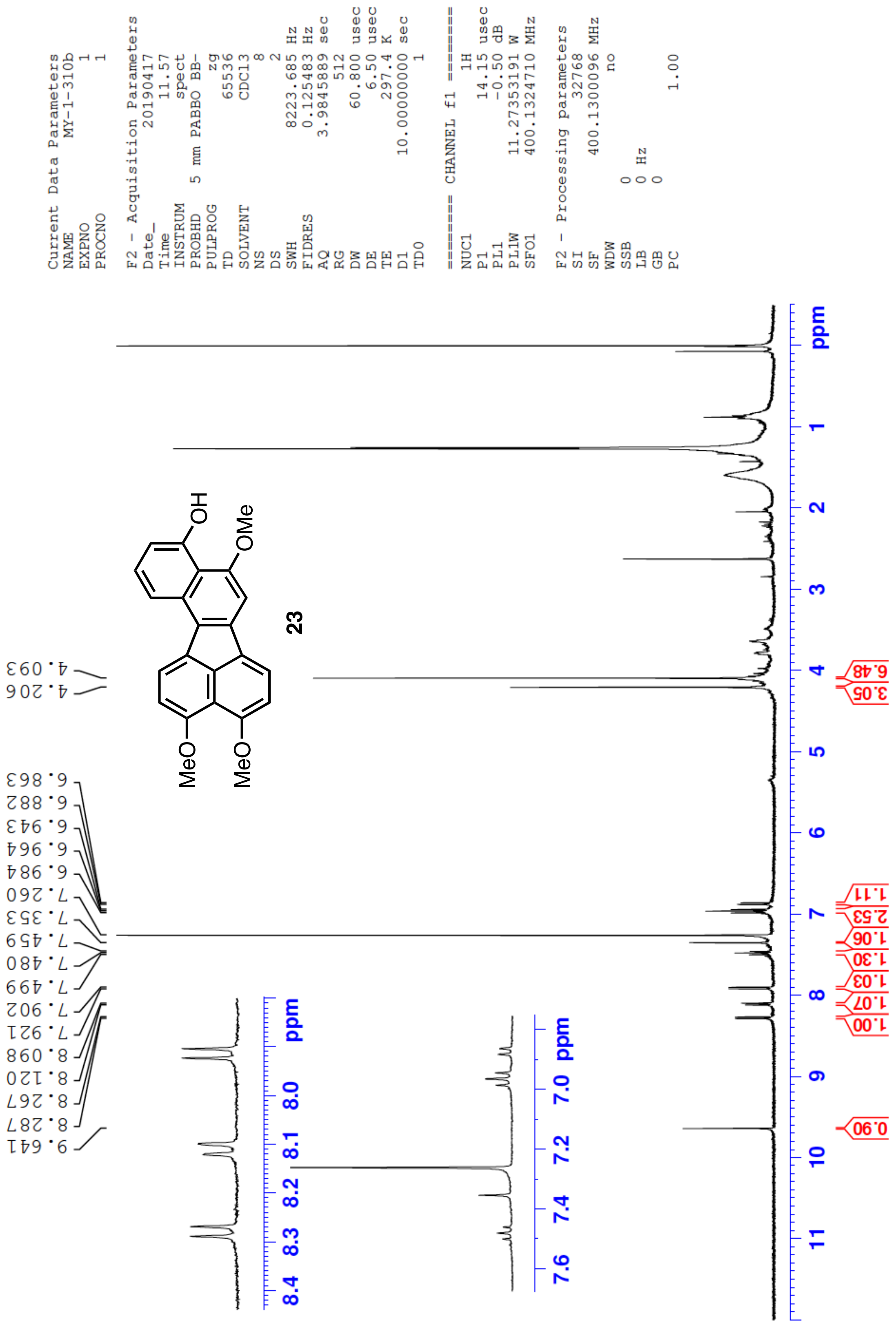

Figure S41. ^{1}H NMR spectrum of **23** in $CDCl_3$.

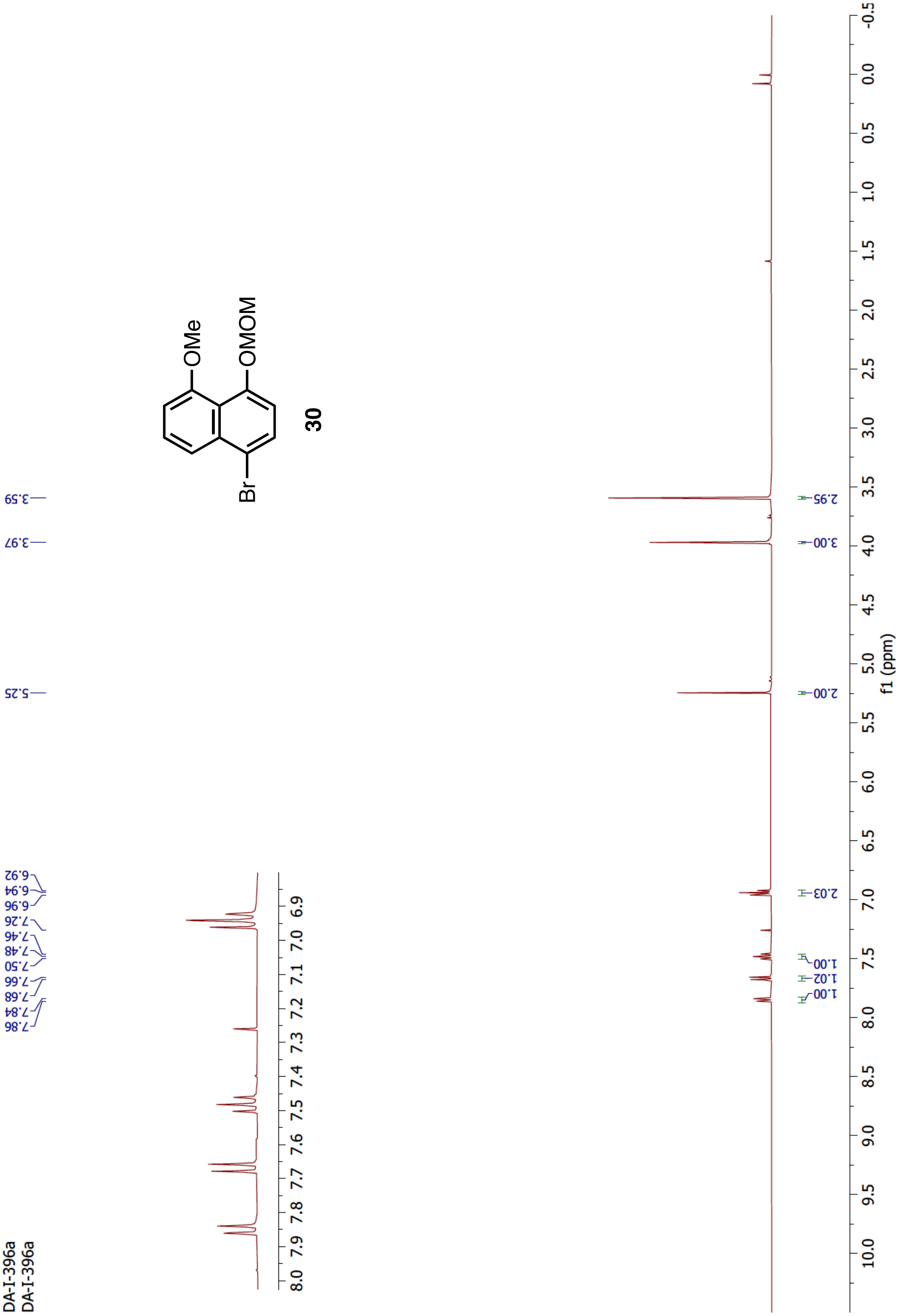

Figure S42. ^{1}H NMR spectrum of **30** in $CDCl_3$.

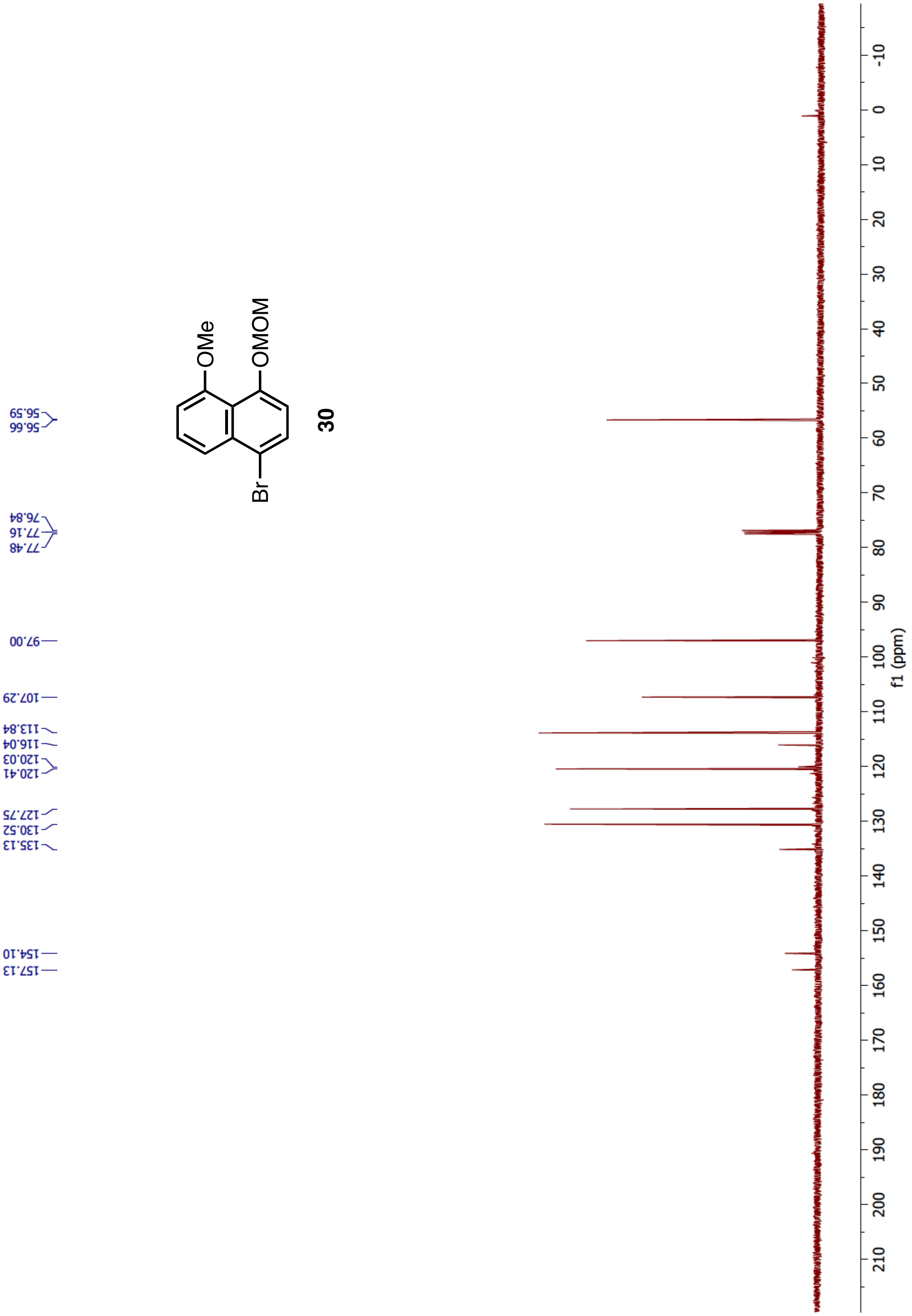

Figure S43. $^{13}C\{^{1}H\}$ NMR spectrum of **30** in $CDCl_3$.

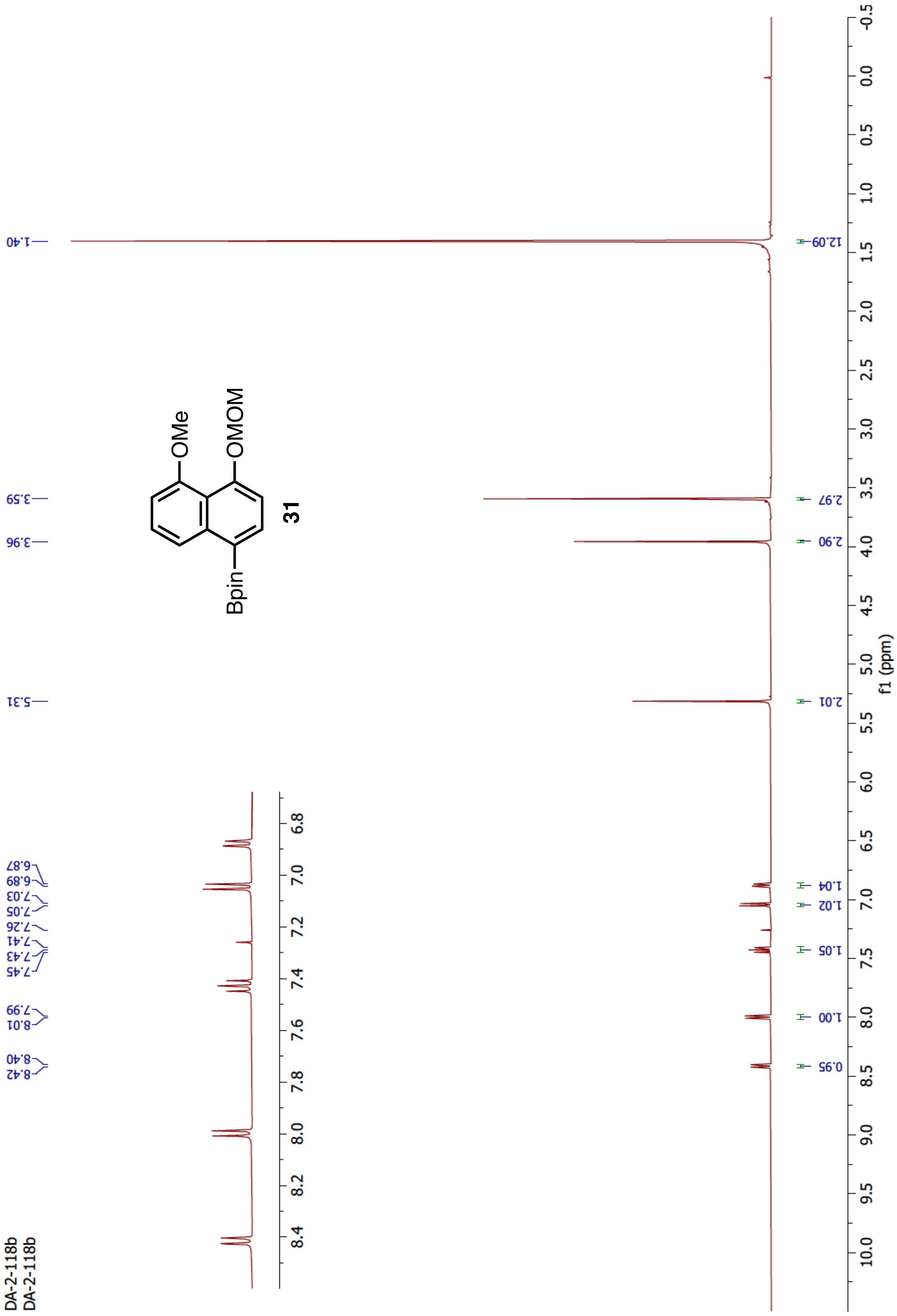

Figure S44. ^{1}H NMR spectrum of **31** in $CDCl_3$.

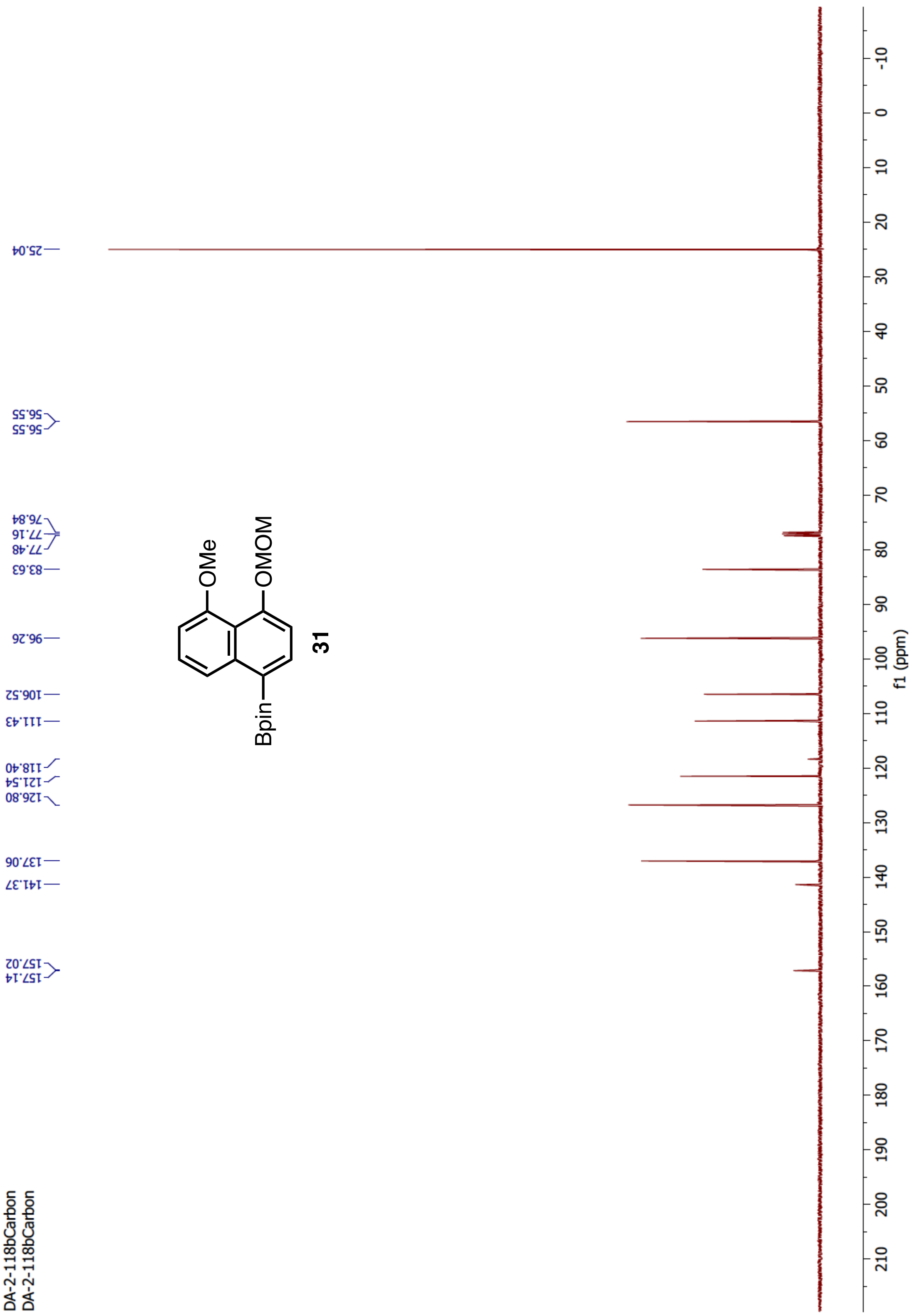

Figure S45. $^{13}C\{^{1}H\}$ NMR spectrum of **31** in $CDCl_3$.

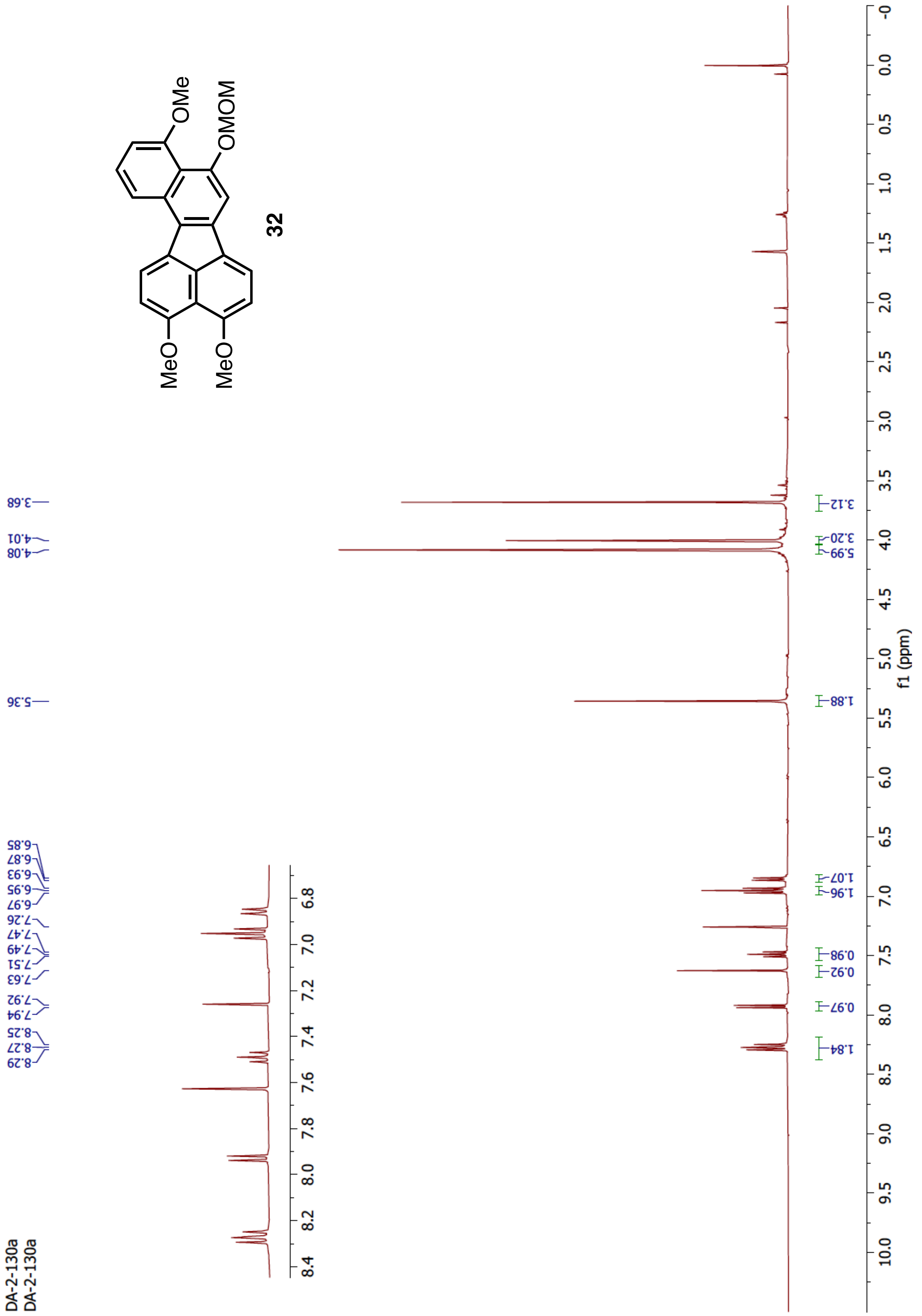

Figure S46. ^{1}H NMR spectrum of **32** in $CDCl_3$.

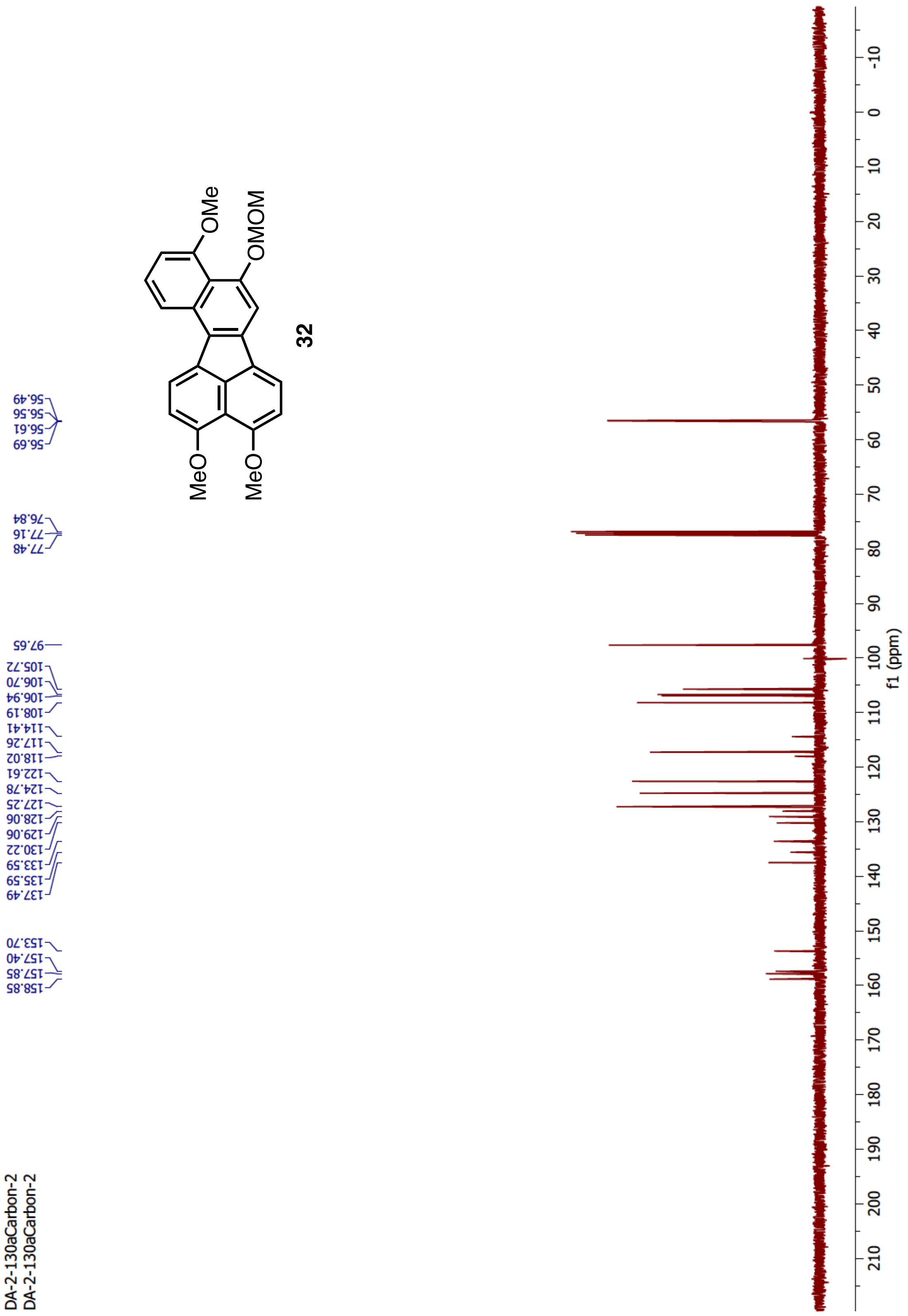

Figure S47. $^{13}C\{^{1}H\}$ NMR spectrum of **32** in $CDCl_3$.

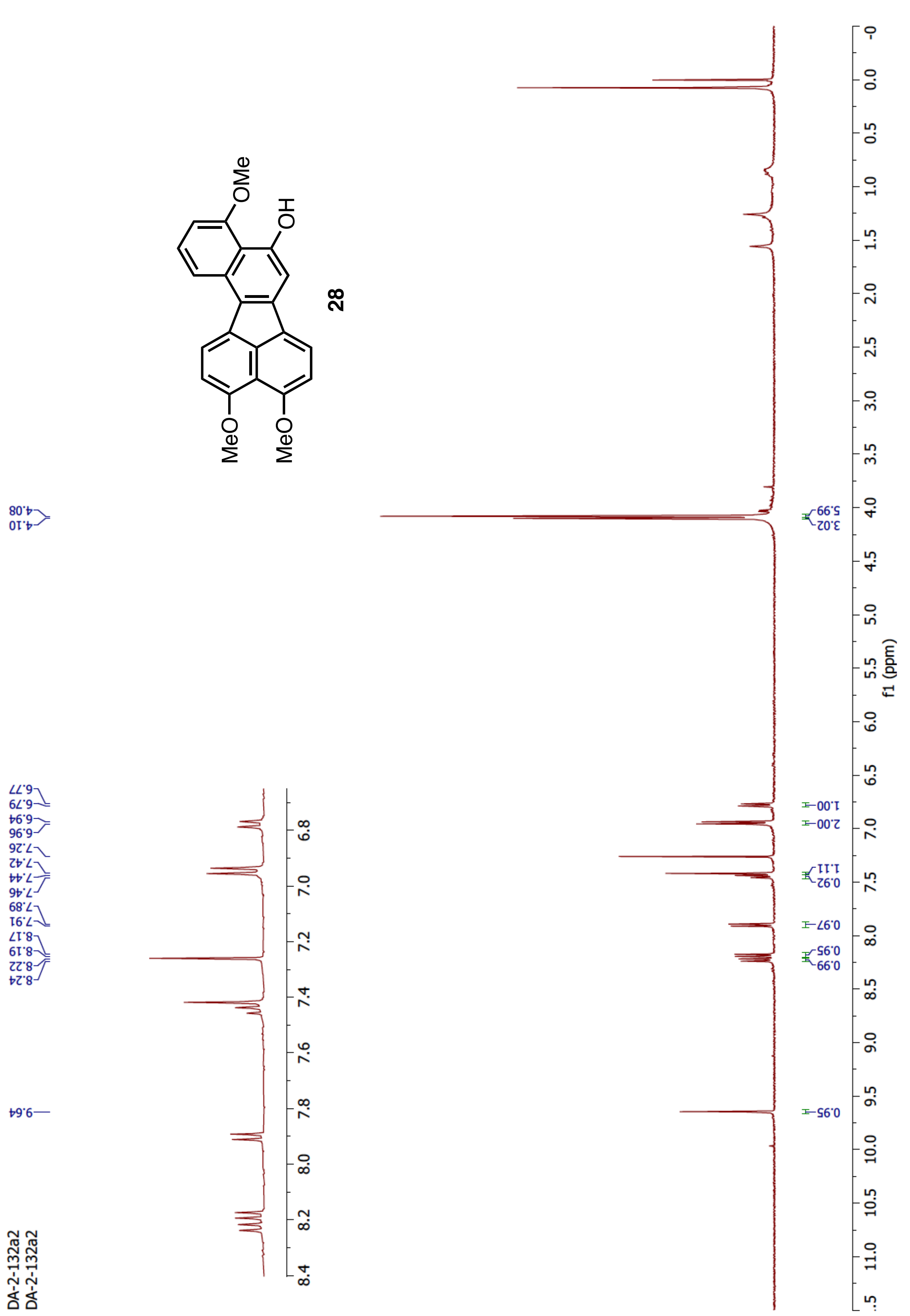

Figure S48. ^{1}H NMR spectrum of **28** in $CDCl_3$.

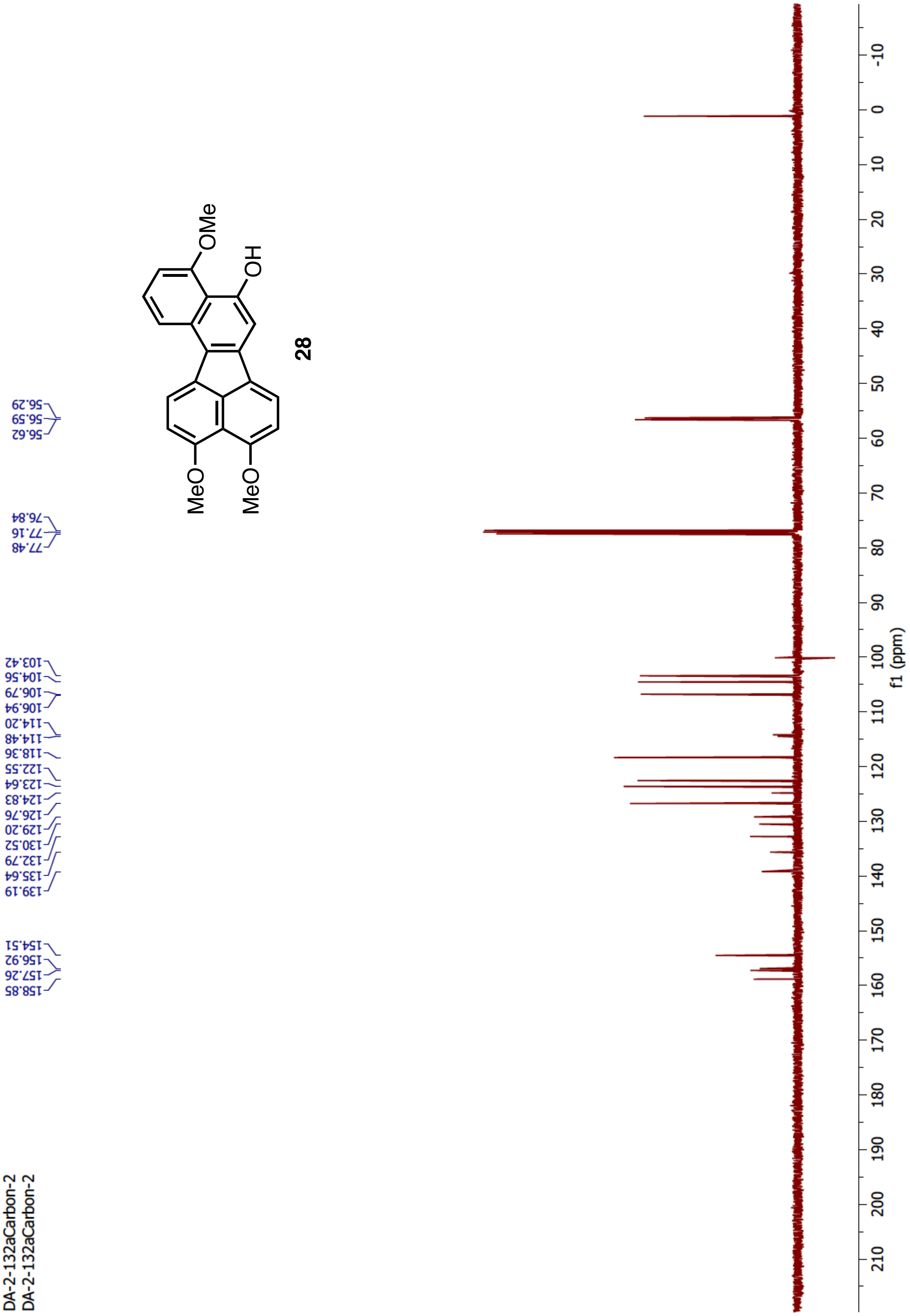

Figure S49. $^{13}C\{^{1}H\}$ NMR spectrum of **28** in $CDCl_3$.

References:

[1] Ahmadli, D.; Sahin, Y.; Calikyilmaz, E.; Şahin, O.; Türkmen, Y. E. *J. Org. Chem.* **2022**, *87*, 6336-6346.

[2] Ahmadli, D.; Türkmen, Y. E. *Tetrahedron Lett.* **2022**, *100*, 153877.

[3] Dyker, G. *J. Org. Chem.* **1993**, *58*, 234.

[4] Pal, S.; Metin, Ö.; Türkmen, Y. E. *ACS Omega* **2017**, *2*, 8689.

[5] (a) Yang, Y.; Lowry, M.; Xu, X.; Escobedo, J. O.; Sibrian-Vazquez, M.; Wong, L.; Schowalter, C. M.; Jensen, T. J.; Fronczek, F. R.; Warner, I. M.; Strongin, R. M. *Proc. Natl. Acad. Sci. USA*, **2008**, *105*, 8829; (b) Türkmen, Y. E. *Turk. J. Chem.* **2018**, *42*, 1398.

[6] Zhao, G.; Xu, G.; Qian, C.; Tang, W. *J. Am. Chem. Soc.* **2017**, *139*, 3360.

[7] Swieca, P.; Spiteller, P. *Eur. J. Org. Chem.* **2022**, e202101576.

[8] Matsuzaka, H.; Hiroe, Y.; Iwasaki, M.; Ishii, Y.; Koyasu, Y.; Hidai, M. *J. Org. Chem.* **1988**, *53*, 3832-3838.

[9] Fan, Y.; Feng, P.; Liu, M.; Pan, H.; Shi, Y. *Org. Lett.* **2011**, *13*, 4494.
